# Prevalence and risk factors for colorectal neoplasia in a self-selected Vietnamese screening cohort undergoing self-funded colonoscopy

**DOI:** 10.1371/journal.pone.0352998

**Published:** 2026-07-13

**Authors:** Huong Thi Thuy To, Nhu Thi Hanh Vu, Hong Nguyen Diem Tong, Dao Thi Anh Nguyen, Thu Dang Anh Phan, Dat Quoc Ngo, Toru Hiyama, Duc Trong Quach

**Affiliations:** 1 Dai Phuoc Polyclinic, Ho Chi Minh City, Vietnam; 2 Department of Internal Medicine and Gastro-Hepato Integrated Research Team (GHIRT-002.TCM2025), School of Medicine, University of Medicine and Pharmacy at Ho Chi Minh City, Vietnam; 3 Department of Histology-Embryology and Pathology, University of Medicine and Pharmacy at Ho Chi Minh City, Ho Chi Minh, Vietnam; 4 Health Service Center, Hiroshima University, Higashihiroshima, Japan; 5 Department of Gastroenterology, Nhan Dan Gia Dinh Hospital, Ho Chi Minh City, Vietnam; Dalin Tzu Chi Hospital, Buddhist Tzu Chi Medical Foundation, TAIWAN

## Abstract

**Objective:**

Several studies have investigated colorectal neoplasia (CRN) in Vietnamese patients who present with lower gastrointestinal symptoms. However, data on subjects without symptoms is limited. This study aimed to determine the prevalence and risk factors for CRN in asymptomatic Vietnamese adults.

**Methods:**

This was a prospective, cross-sectional, single-center study. Participants were consecutively recruited from asymptomatic individuals who were self-selected to undergo self-funded screening colonoscopy. CRN was defined as the presence of adenoma, sessile serrated lesions, or colorectal cancer. Advanced CRN included adenoma ≥ 1 cm, with villous features or high-grade dysplasia; sessile serrated lesion ≥ 1 cm or with dysplasia; traditional serrated adenoma; or colorectal cancer. Multivariable logistic regression was performed to identify independent risk factors for CRN, adjusting for age, sex, BMI, family history of colorectal cancer, smoking status, and alcohol consumption.

**Results:**

There were 714 patients, with a median age of 51 (18–79 years) and a female-to-male ratio of 1:1.46. In this screening-attending cohort, the prevalence of overall CRN and advanced CRN were 26.2% and 9.0%, respectively. In the multivariate analysis, factors significantly associated with CRN included increasing age per 10-year increment (odds ratio [OR]: 1.76; 95% confidence interval [CI]: 1.47–2.11; p < 0.001), body mass index ≥ 23 kg/m^2^ (OR: 1.70; 95% CI: 1.16–2.50; p = 0.006), alcohol consumption (OR: 1.83, 95% CI: 1.10–3.04, p = 0.020), and family history of colorectal cancer (OR: 2.43; 95% CI: 1.36–4.37; *p* = 0.003).

**Conclusions:**

CRN was prevalent in this self-selected screening-attending cohort in a private clinical setting. Increasing age, overweight, and family history of colorectal cancer were independent factors associated with CRN.

## Introduction

Colorectal cancer (CRC) remains a substantial global health problem, ranking as the third most common cancer and the second leading cause of cancer-related mortality worldwide. In Vietnam, CRC ranks as the fourth most common malignancy among men, and the third among women, with an increasing trend observed over recent years [1]. Early detection and removal of colorectal neoplasia (CRN) via colonoscopy are essential for reducing CRC incidence and mortality [2]. However, one of the most significant challenges is the trend for CRC to be diagnosed in patients younger than the age of 50 years, particularly in the Asia-Pacific region [3]. A hospital-based study of symptomatic CRC patients in Vietnam revealed that the proportion of early-onset CRC in the Vietnamese population was 28%. In this group, nearly half of the patients reported their symptoms as intermittent patterns, and approximately 22.3% did not present with alarming symptoms [4]. Moreover, evidence has indicated that CRC largely remains asymptomatic until it has progressed to an advanced stage [5]. These clinical characteristics may complicate the differentiation of CRC from benign or functional bowel disorders, resulting in delayed diagnosis. Therefore, CRC screening for early detection plays a critical role, including among asymptomatic individuals, defined as those without lower gastrointestinal symptoms or alarm features.

Currently, CRC screening programs are implemented in most European countries, Canada, specific regions in North and South America, other Asian countries and Oceania [6]. However, in Vietnam, the lack of infrastructure, funding constraints, and limited clinical capacity and manpower have prevented the establishment of a population-based CRC screening program. Consequently, identifying the prevalence and associated risk factors for CRN may contribute preliminary evidence relevant to future CRC screening strategies in Vietnam.

Previous studies have identified several risk factors associated with CRN, including age, male sex, family history, obesity, smoking, alcohol consumption, physical inactivity, and dietary factors such as high red meat intake and low fiber consumption [7,8]. Older age is considered one of the most important risk factors; nonetheless, the age at which CRC screening should be started differs worldwide. Due to the rising global prevalence of early-onset CRC, several countries have lowered the recommended age for CRC screening. The US guideline has proposed lowering the screening age to 45 years for all adults at average risk [9]. In the Asia-Pacific Region, Japan offers screening with annual fecal immunochemical tests at the age of 40 years [10], whereas Taiwan and Korea offer biennial fecal immunochemical tests starting at 50 years [11,12]. In Vietnam, previous research has shown that the age of onset of advanced CRN and CRC is younger than that in other countries in the Asia-Pacific region; therefore, the screening age threshold would be lower accordingly [4,13]. Nevertheless, there is a lack of existing data to support appropriate recommendations. Previous research on CRN in Vietnam has predominantly focused on symptomatic patients, leaving a substantial gap in evidence regarding the age-stratified prevalence—particularly among adults under 40 years—and the risk factors of CRN in asymptomatic Vietnamese individuals. To date, no nationally endorsed CRC screening guideline has been formally implemented in Vietnam. Screening is largely opportunistic and self-funded, with colonoscopy performed across a broad adult population in routine practice. Robust data from such settings are needed to better inform evidence-based screening policies. Consequently, this study aimed to determine the prevalence of CRN in asymptomatic Vietnamese adults undergoing self-funded screening colonoscopy and to identify significant risk factors associated with these lesions.

## Materials and methods

### Study participants

This was a prospective, cross-sectional, single-center study. Participants were consecutively recruited from asymptomatic adults who elected to undergo self-funded screening colonoscopy as part of routine health examinations at Dai Phuoc Polyclinic, Ho Chi Minh City, Vietnam, from 10/04/2022 to 31/03/2023. The eligibility criteria for this study were: age ≥ 18 years and asymptomatic status, defined as the absence of lower intestinal symptoms (including recent changes in bowel habits, abdominal pain, or hematochezia) and the absence of alarm features, such as anemia or unintentional weight loss exceeding 5% of body weight within 6–12 months. Asymptomatic status was determined prior to colonoscopy through standardized pre-procedural clinical evaluation conducted by experienced gastroenterologists. Lower gastrointestinal symptoms and alarm features were assessed using structured clinical interviews and physical examination. All participants underwent routine pre-procedural laboratory testing, including complete blood count, and hemoglobin levels were reviewed to identify anemia. The exclusion criteria included a history of endoscopic polypectomy, CRC or colorectal surgery, inherited cancer syndromes, coagulation disorders, inflammatory bowel disease, unqualified bowel preparation (i.e., a total score of < 6 or a segment score of < 2 according to the Boston Bowel Preparation Scale) [14]; incomplete colonoscopies; and withdrawal time of less than 6 minutes.

Overweight was defined as a body mass index (BMI) of 23.0 kg/m^2^ or greater for the Asian population [15]. Smoking conditions were defined as having smoked when the participant was currently smoking or had ever smoked. Alcohol consumption was defined as drinking more than once per month [16]. The definition of a family history of CRC was the presence of at least one affected first-degree relative at any age.

All eligible participants signed a written informed consent form. This study was carried out in accordance with the Helsinki Declaration and was approved by the Board of Ethics in Biomedical Research of Pham Ngoc Thach University of Medicine (ID number: 663/TDHYKPNT-HDDD, signed on April 08, 2022).

### Colonoscopy procedure

All subjects were provided with dietary instructions prior to colonoscopy, and bowel preparation was conducted with 3 liters of polyethylene glycol-based material (Fortrans®, Ipsen Pharma, Paris, France). Colonoscopies were performed by five experienced endoscopists with the Olympus Evis Exera III High Definition CV-190 (Olympus Co., Ltd., Tokyo, Japan) under conscious or deep sedation with propofol (Propofol®Lipuro 1%, B. Braun, Melsungen, Germany). All participating endoscopists had adenoma detection rates of more than 30% and had performed at least 3,000 colonoscopic procedures over the last five years, indicating adherence to established quality standards. Cecal intubation was confirmed by identifying the appendiceal orifice, cecal valve, or by intubating the ileum. Stopwatches were used to record withdrawal times, which had to be at least 6 minutes, excluding time for polypectomies.

The morphology, location, and size of all the detected polyps were recorded and analysed by the participating endoscopists using standardized data collection sheets. The polyps were classified according to the Paris classification into polypoid (0-Is, 0-Ip), nonpolypoid (0-IIa, 0-IIb, 0-IIc), and excavated (0-III) types [17]. The location of the polyps was classified as the cecum, ascending, transverse, descending, sigmoid colon, or rectum. The size of the lesions was determined by comparison with open biopsy forceps (7 mm width), closed biopsy forceps (3 mm width), or polypectomy snares of known diameters. The Narrow-Band Imaging International Colorectal Endoscopic (NICE) classification was used to categorize lesions based on color, vascular pattern, and surface structure, with type 1 indicating hyperplastic polyps, type 2 representing adenomas, and type 3 suggesting invasive cancer [18]. All the detected polyps were removed using standard procedures determined by the endoscopists, including biopsy forceps, cold snare, hot polypectomy, endoscopic mucosal resection, and endoscopic submucosal dissection.

### Histopathological analysis

All resected lesions were collected in distinct jars and preserved in 10% formalin. Sections were cut at a thickness of 4 µm and stained with hematoxylin-eosin. Histopathological evaluation of all resected specimens was performed by expert gastrointestinal pathologists at the Department of Histology-Embryology and Pathology, University of Medicine and Pharmacy at Ho Chi Minh City. Pathologists were blind to the patients’ risk factor profiles. In cases of diagnostic uncertainty, slides were independently reviewed by a second senior pathologist, and discrepancies were resolved by consensus to minimize inter-observer variability. Colorectal polyps were pathologically classified into CRN and colorectal non-neoplasia. CRN was defined as adenoma (tubular adenoma, villous adenoma, tubulovillous adenoma), sessile serrated lesions, or CRC. CRN was further classified as advanced CRN and non-advanced CRN. The former was defined as (i) tubular adenoma ≥1 cm or any adenoma with villous features or high-grade dysplasia regardless of size, (ii) sessile serrated lesion ≥ 1 cm or with cytologic dysplasia, (iii) traditional serrated adenoma of any size, or (iv) CRC [19]. If participants had two or more lesions, the most advanced lesion was used for statistical analysis.

### Statistical analysis

Statistical analyses were performed with IBM SPSS Statistics for Windows, version 23.0 and MedCalc® Statistical Software version 19.6.1 (MedCalc Software Ltd., Ostend, Belgium). The Kolmogorov-Smirnov test was used to determine the normality of continuous variables. Variables conforming to a normal distribution were presented as the means and standard deviation (SD) and were analysed using the t-test. Variables that were not normally distributed were presented as medians (with upper and lower quartiles) and analyzed using the Mann-Whitney U test. Pearson’s chi-square test was used for the proportion analysis of categorical data. The prevalence and risk factors for CRN were determined on a per-patient basis. The 95% confidence intervals (CIs) for prevalence rates were calculated using the Clopper-Pearson exact method. Univariate and multivariate logistic regression analyses revealed the factors correlated with CRN. All 2-tailed p values less than 0.05 were considered statistically significant.

In the primary multivariable logistic regression analysis, age was modeled as a continuous variable expressed per 10-year increment. An exploratory secondary analysis using an age cutoff of 40 years was also performed because previous Vietnamese and Asia-Pacific studies have suggested a relatively younger age distribution of CRN in this region [4,10].

## Results

### Characteristics of participants

During the study period, 746 asymptomatic subjects were referred for colonoscopy. Among 735 eligible cases, 3 were excluded due to incomplete colonoscopy, 6 due to inadequate bowel preparation with a total score of < 6 or a segment score of < 2 according to the Boston Bowel Preparation Scale, and 3 due to withdrawal time < 6 minutes, resulting in a cecal intubation rate of 99.6%.

**Fig 1:**
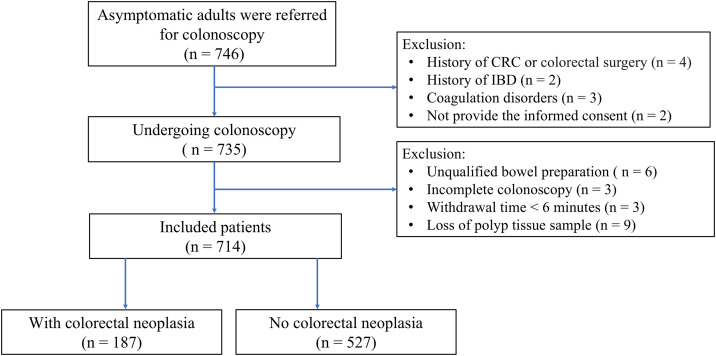
Flow chart of participant enrollment.

All procedures had adequate bowel preparation and a mean withdrawal time of 7.3 ± 0.6 minutes, consistent with established quality standards. A total of 714 participants were included in the analysis (Fig 1).

The median age of the individuals was 51 years, ranging from 18–79 years, and 88.4% were 40 years or older. The female-to-male ratio was 1:1.46. The mean BMI was 23.49 ± 2.98 kg/m^2^. Overweight was found in 405 subjects (56.7%). The proportions of participants who had a history of smoking and alcohol consumption were 14.7% and 29.1%, respectively. A total of 60 subjects (8.4%) had a family history of CRC in their first-degree relatives. The demographic and clinical features of the participants are described in Table 1.

**Table 1:** Characteristics of participants with and without colorectal neoplasia.

Characteristics	Total (n, %) N = 714	No colorectal neoplasia (n, %) N = 527	Colorectal neoplasia (n, %) N = 187	p value
Sex				
Male	290 (40.6)	194 (36.8)	96 (51.3)	0.001
Female	424 (59.4)	333 (63.2)	91 (48.7)	
Age				
< 40	83 (11.6)	72 (13.7)	11 (5.9)	
40-49	221 (31.0)	175 (33.2)	46 (24.6)	0.157 ^a^
50-59	238 (33.3)	177 (33.6)	61 (32.6)	0.134 ^b^
60-69	146 (20.4)	88 (16.7)	58 (31.0)	0.004 ^c^
≥ 70	26 (3.6)	15 (2.8)	11 (5.9)	0.026 ^d^
BMI (kg/m^2^)				
≥ 23	405 (56.7)	274 (52.0)	131 (70.1)	< 0.001
< 23	309 (43.3)	253 (48.0)	56 (29.9)	
Smoking				
Yes	105 (14.7)	64 (12.1)	41 (21.9)	0.001
No	609 (85.3)	463 (87.9)	146 (78.1)	
Alcohol consumption				
Yes	208 (29.1)	132 (25.0)	76 (40.6)	< 0.001
No	506 (70.9)	395 (75.0)	111 (59.4)	
Family history of CRC				
Yes	60 (8.4)	35 (6.6)	25 (13.4)	0.04
No	654 (91.6)	492 (93.4)	162 (86.6)	

^a, b, c, d^ p-values represent comparisons of the 40–49, 50–59, 60–69, and ≥ 70 years age groups, respectively, with the reference group (< 40 years)

OR: Odds ratio, CI: Confidence interval, BMI: Body mass index, CRC: Colorectal cancer

#### Prevalence and characteristics of colorectal neoplasia.

Among the 714 asymptomatic subjects, 187 were diagnosed with CRN, yielding a crude prevalence of 26.2% (95% CI: 23.0%−29.6%) and an age-standardized prevalence of 21.3% based on the 2024 Vietnam Intercensal Population and Housing Survey [20]. Advanced CRN was detected in 64 patients, corresponding to a crude prevalence of 9.0% (95% CI: 7.0%−11.3%) and an age-standardized prevalence of 6.4%, of whom 2 patients (0.3%, 95% CI: 0.03%−1.01%) had adenocarcinoma.

**Fig 2:**
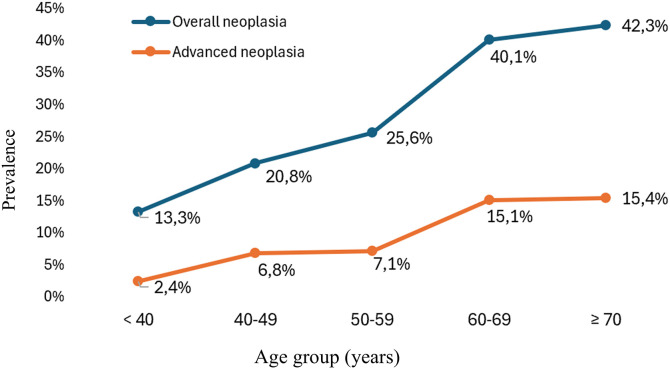
Prevalence of overall neoplasia and advanced neoplasia according to age.

Our results indicated that the prevalence of overall CRN and advanced CRN increased with age (Fig 2). CRN was found in 13.3% of the < 40 years age group, which increased to 20.8% of the 40–49 years age group, 25.6% of the 50–59 years age group, 40.1% of the 60–69 years age group, and 42.3% of the ≥ 70 years age group.

A total of 321 lesions from 187 patients were diagnosed with CRN. The most common locations were the sigmoid colon (34.3%) and transverse colon (27.5%). The mean size of the CRN was 7.0 ± 5.1 mm. Pathological analysis revealed that the majority of lesions were tubular adenomas (89.1%). The endoscopic and pathological features of CRN are described in Table 2.

**Table 2:** Endoscopic and pathological characteristics of colorectal neoplasia.

Characteristics	Colorectal neoplasms (n, %) N = 312
Location	
Cecum	11 (3.5)
Ascending colon	40 (12.8)
Transverse colon	86 (27.5)
Descending colon	44 (14.1)
Sigmoid colon	107 (34.3)
Rectum	24 (7.7)
Size (mm)	
≤ 5	168 (53.8)
6-10	97 (31.1)
11-20	39 (12.5)
> 20	8 (2.6)
Shape (Paris classification)	
0-Ip	22 (7.1)
0-Isp	45 (14.4)
0-Is	204 (65.4)
0-IIa	37 (11.9)
0-IIb	0 (0)
0-IIc	2 (0.6)
NICE type	
NICE 1	49 (15.7)
NICE 2	258 (82.7)
NICE 3	5 (1.6)
Pathology	
Tubular adenoma	278 (89.1)
Tubulovillous adenoma	14 (4.5)
Sessile serrated lesion	18 (5.8)
Adenocarcinoma	2 (0.6)

### Risk factors for colorectal neoplasia

In the primary multivariable analysis, increasing age per 10-year increment (odds ratio [OR]: 1.76; 95% CI: 1.47–2.11; p < 0.001), BMI ≥ 23 kg/m² (OR: 1.70; 95% CI: 1.16–2.50; p = 0.006), alcohol consumption (OR: 1.83; 95% CI: 1.10–3.04; p = 0.020), and family history of CRC (OR: 2.43; 95% CI: 1.36–4.37; p = 0.003) were independently associated with overall CRN (Table 3). For advanced CRN, increasing age may be associated with the outcome in the exploratory multivariable analysis; however, the estimates were imprecise, with relatively wide confidence intervals because of the limited number of advanced CRN cases (Supplementary Table 1 S1 Table).

**Table 3:** Multivariable logistic regression analysis of factors associated with colorectal neoplasia.

Risk factors	Overall colorectal neoplasia		
	OR	95% CI	p value
Male	1.16	0.71-1.91	0.551
Age (per 10-year increase)	1.76	1.47- 2.11	< 0.001
BMI ≥ 23 kg/m^2^	1.70	1.16-2.50	0.006
Smoking	1.23	0.72-2.11	0.440
Alcohol consumption	1.83	1.10-3.04	0.020
Family history of CRC	2.43	1.36-4.37	0.003

*OR: odds ratio, CI: confidence interval, BMI: Body mass index, CRC: Colorectal cancer*

In the exploratory secondary analysis using an age threshold of ≥ 40 years, participants aged ≥ 40 years showed higher odds of overall CRN (OR: 2.79; 95% CI: 1.40–5.56; p = 0.004). BMI ≥ 23 kg/m2 (OR: 1.92; 95% CI: 1.32–2.79; p = 0.001) and family history of CRC (OR: 2.29; 95% CI: 1.29–4.05; p = 0.005) were also associated with overall CRN. Similarly, participants aged ≥ 40 years showed a possible increase in the odds of advanced CRN in the exploratory analysis, although the estimates were imprecise because of the limited number of events (OR: 5.20; 95% CI: 1.22–22.11; *p* = 0.025) (Supplementary Table 2 S2 Table).

## Discussion

Our results in asymptomatic individuals ≥ 18 years who underwent self-funded screening colonoscopy in a private clinical setting indicated that the prevalence of CRN and advanced CRN were 26.2% and 9%, respectively. The risk factors significantly associated with CRN were increasing age, overweight status, and family history of CRC. The Asia-Pacific region has the most significant burden of CRC cases (51.8%) and CRC mortality (52.4%) globally [21]. The prevalence of overall CRN and advanced CRN in asymptomatic subjects from Asia Pacific studies ranged from 19.2–36.0%, and from 1.7–15.4%, respectively [22–24]. Nevertheless, the majority of participants in these studies were aged 40 years or older. Another prospective multinational multicenter survey of asymptomatic subjects over 16 years old in 11 Asian cities reported that the prevalence of CRN and advanced CRN were 18.5% and 4.5%, respectively [25]. However, these prevalences were published in 2007; since then, there have been changes in the ADR benchmark and colonoscopy quality standards. This may limit the direct comparability of our findings. Overall, the prevalence of CRN and advanced CRN in our study was higher than that reported in previous studies conducted in the Asia Pacific region; however, differences in study design, population characteristics, and screening context may partly explain these variations.

Currently, no published data are available on CRN prevalence among asymptomatic individuals in Vietnam. The only available local data derived from a cohort of patients with irritable bowel syndrome, with rates of 9.1% and 6.9%, respectively [26]. The high proportion of advanced CRN in this study may be attributed to the inclusion of symptomatic patients undergoing colonoscopy, rather than individuals undergoing voluntary screening colonoscopy without symptoms. Furthermore, the relatively small sample size may have contributed to the disproportionate ratio between CRN and advanced CRN. To the best of our knowledge, this is the first study evaluating the prevalence and risk factors for CRN among asymptomatic Vietnamese adults. Consequently, additional research is needed to provide a more comprehensive understanding of this issue.

In our study, the number of participants excluded for quality-control reasons was small (12/735). Given this very small proportion of exclusions, their impact on overall prevalence estimates and risk factor associations is likely limited. In addition, although 15.3% of lesions were endoscopically classified as NICE type I, all were histologically confirmed as CRN. There may be discrepancies between endoscopic appearance and pathological diagnosis, particularly in small lesions where optical differentiation between diminutive hyperplastic, serrated, and low-grade neoplastic lesions can be challenging [27]. In our study, NICE type I discordance was largely confined to lesions ≤ 5 mm and was not significantly associated with lesion location. These findings highlight the limitations of real-time optical diagnosis in routine clinical practice, particularly for diminutive lesions. However, all colonoscopies were performed by experienced endoscopists with established quality indicators (adenoma detection rate >30%), and no significant inter-endoscopist variation in NICE classification accuracy was observed. Importantly, all lesions were resected and histologically examined, which may mitigate the impact of optical misclassification on study outcomes.

Evidence from Vietnam has demonstrated a substantial proportion of early-onset CRC [4], while screening practices in some Asian countries, such as Japan, begin at age 40 [10]. In addition, a large prospective colonoscopy study in asymptomatic Korean adults—reflecting a screening-attending population—has shown that CRN may occur before the age of 50 [28]. Together, these findings support the rationale for exploring a lower age threshold in this context.

In the primary multivariable analysis, increasing age modeled as a continuous variable per 10-year increment remained independently associated with both overall CRN and advanced CRN. BMI ≥ 23 kg/m², alcohol consumption, and family history of CRC were also independent risk factors for overall CRN. These findings are consistent with those from a large prospective multinational study in asymptomatic Asian populations, which identified advanced age, male sex, and family history of CRC as key risk factors for CRN [25]. Previous studies have also indicated that increasing age is an important risk factor for the development of CRN and CRC [22,24]. In Vietnam, several studies have suggested a relatively younger age distribution of CRN and CRC compared with that reported in some other countries. A retrospective study conducted on 2,525 outpatients with advanced CRN revealed that the rate of advanced CRN in the group under 50 years of age was 23.4%, whereas that in the group < 40 years of age was 8.2% [13]. Another prospective study reported a significantly high proportion of early-onset CRC (i.e., < 50 years of age) of 28%, of which 11% of patients were under 40 years of age [4]. Therefore, these data suggest a potential burden of CRC in younger adults within screening-attending populations. Based on these prior observations, we additionally performed an exploratory secondary analysis using an age threshold of ≥ 40 years. In this exploratory model, individuals aged ≥40 years had significantly increased odds of both overall CRN and advanced CRN. These observations highlight the need for further investigation into age-specific risk stratification in this context. However, this observation should be interpreted as hypothesis-generating, and confirmation from large-scale, population-based studies is required before informing national policy.

It has been proposed that adherence to screening recommendations may be enhanced if individuals recognize that these recommendations are tailored to their personal risk profile rather than being determined by an arbitrary age criterion [29]. In addition to traditional CRC risk factors, overweight and obesity have been identified as environmental factors for CRN and CRC [30,31]. The proposed mechanism involves insulin resistance and elevated levels of insulin-like growth factor, leptin, and vascular endothelial growth factor, leading to chronic inflammation and cancer [32]. In Asia, obesity is less prevalent, and the mean BMI is thus lower than that in Western countries [33]. Hence, in this study, we defined overweight as BMI ≥ 23 kg/m^2^, which is the recommended threshold for the Asian population [15]. Our results revealed that being overweight was an independent risk factor for CRN. Previous studies in China, Thailand, and Korea also used a cut-off BMI ≥ 23 kg/m^2^ to define overweight and concluded that this cut-off was an independent risk factor for detecting CRN and advanced CRN [23,34,35]. Nonetheless, a recent case-control study in Vietnam demonstrated no statistically significant correlation between overweight and CRN [36]. In this study, the investigators did not specify whether the participants were asymptomatic or symptomatic. Moreover, the control group consisted of individuals who had negative immunochemical fecal occult blood test results but did not undergo colonoscopy. Consequently, it remains uncertain whether adenomas were truly absent among participants of the control group. Hence, further research is required to ascertain this relationship, particularly in the Vietnamese population.

A family history of CRC is also a known risk factor for CRC, likely due to a combination of shared polygenic and shared environmental risks [37,38]. Our results indicated that first-degree relatives with CRC were related to CRN (OR: 2.29, 95% CI: 1.29–4.05), which is consistent with prior research.[25,39,40] A meta-analysis of 13 colonoscopy studies concluded that individuals with a family history of CRC had a significantly higher adenoma prevalence than those without family history (OR: 1.7, 95% CI: 1.4–3.5) [39]. Many international and national guidelines included recommendations for screening individuals with a family history of CRC [3,41,42]. Furthermore, prior study has demonstrated that the two most effective incentives for individuals to participate in a screening program are their awareness of the significance of a family history of CRC and the recommendation of physicians to undergo CRC screening [43]. Therefore, our findings describe risk patterns among asymptomatic Vietnamese individuals undergoing self-funded screening colonoscopy in a private clinical setting and may contribute to understanding risk distribution within similar screening populations.

While BMI ≥ 23 kg/m² and a first-degree relative with CRC were identified as independent risk factors for overall CRN, these associations did not reach statistical significance for advanced CRN. This lack of significance is likely attributable to the limited number of advanced CRN cases, which could potentially affect statistical power. Accordingly, the absence of statistical significance for certain variables should not be interpreted as evidence of no association, but rather as a reflection of limited statistical power. Moreover, biological differences in the progression from non-advanced to advanced neoplasia may contribute to this finding, particularly in the context of early detection through screening colonoscopy.

This study had several limitations. First, it was a single-center study involving individuals who voluntarily underwent self-funded screening colonoscopy in a private clinical setting, which may have introduced selection bias. Participants were likely more health-conscious and had better healthcare access than the general Vietnamese population, potentially resulting in higher prevalence estimates than those in the broader average-risk population. Participants were likely more health-conscious and had better healthcare access than the general Vietnamese population, potentially resulting in higher prevalence estimates than those in the broader average-risk population. Importantly, our study was not designed to provide population-level prevalence estimates, but rather to describe the burden and associated risk factors of CRN within a real-world screening-attending population, which is common in Vietnam in the absence of organized national screening programs. Therefore, the findings should be interpreted cautiously and validated in large-scale, multicenter, population-based studies before being generalized to national CRC screening strategies or used to inform screening policy recommendations. Nonetheless, the single-center setting allowed for standardized colonoscopy procedures and centralized histopathological evaluation, which may enhance internal consistency.

Second, although asymptomatic status was determined through structured clinical assessment by experienced gastroenterologists—as is typical in routine screening practice—it was not based on a formally validated instrument. Therefore, mild or intermittent gastrointestinal symptoms may have been underrecognized, potentially resulting in some degree of symptom misclassification and recall bias, which should be considered when interpreting the observed prevalence estimates. Third, some factors potentially associated with CRN, such as diabetes mellitus, dyslipidemia, physical inactivity, and dietary factors including high red meat intake and low fiber consumption, were not considered in our study. Furthermore, smoking and alcohol consumption were categorized using simplified definitions and were not evaluated quantitatively. These limitations may introduce residual confounding, and the observed associations should therefore be interpreted cautiously. E-value analyses indicated that unmeasured confounding of at least moderate magnitude would be required to fully explain away the observed associations (Supplementary Table 3 S3 Table). Future studies incorporating more detailed exposure assessment and broader metabolic profiling are warranted to better delineate these associations. Fourth, the number of advanced CRN cases was relatively limited, leading to wider confidence intervals and potential instability of multivariable estimates. Therefore, associations involving advanced CRN should be interpreted cautiously. Fifth, owing to the cross-sectional design of this study, temporal relationships cannot be established, and therefore causal inferences cannot be made.

In this screening-attending population, our data suggests a notable burden of CRN within this screening-attending cohort. Taken together with prior Vietnamese reports suggesting a considerable proportion of early-onset CRC, these observations highlight the need for further investigation into age-specific risk stratification in this context. Because Vietnam currently lacks an established national CRC screening program, confirmation from large-scale, population-based studies is essential before these observations could inform the development of future screening strategies.

In conclusion, CRN was prevalent in this self-selected screening-attending cohort undergoing self-funded colonoscopy. Increasing age, overweight status, alcohol consumption, and family history of CRC were independently associated with CRN in this screening-attending population. These findings should be interpreted with caution and may not be directly generalizable to the average-risk Vietnamese population.

## Supporting information

S1 Table Supplementary Table 1. Exploratory multivariable logistic regression analysis of factors associated with advanced colorectal neoplasia. (DOCX)

S2 Table Supplementary Table 2. Exploratory multivariable logistic regression analysis of factors associated with colorectal neoplasia and advanced colorectal neoplasia. (DOCX)

S3 Table Supplementary Table 3. E-values for significant factors associated with colorectal neoplasia in the primary multivariable analysis. (DOCX)

S1 File STROBE checklist. (DOCX)

S2 File Inclusivity in global research. (DOCX)

S3 File Data. (XLS)

## Abbreviations

BMI: body mass index
CRN: colorectal neoplasia
OR: odds ratio
CI: confidence interval.
