# Supplementary material for: Prevalence and risk factors for colorectal neoplasia in a self-selected Vietnamese screening cohort undergoing self-funded colonoscopy
Source: PLoS One. 2026 Jul 13;21(7):e0352998. doi: 10.1371/journal.pone.0352998 (PMC13362143; doi:10.1371/journal.pone.0352998)
Supplement: S1 Table — (DOCX) [file pone.0352998.s001.docx]

**Supplementary Table 1: Exploratory multivariable logistic regression analysis of factors associated with advanced colorectal neoplasia**

| Risk factors | Advanced colorectal neoplasia | | |
| --- | --- | --- | --- |
|  | OR | 95% CI | p value |
| Male | 1.42 | 0.67-2.98 | 0.360 |
| Age (per 10-year increase) | 1.91 | 1.46-2.51 | < 0.001 |
| BMI ≥ 23 kg/m^2^ | 1.43 | 0.79-2.56 | 0.234 |
| Smoking | 0.71 | 0.33-1.55 | 0.394 |
| Alcohol consumption | 2.00 | 0.94-4.26 | 0.071 |
| Family history of CRC | 2.06 | 0.93-4.58 | 0.077 |

*OR: odds ratio, CI: confidence interval, BMI: Body mass index, CRC: Colorectal cancer*
