# Supplementary material for: Prevalence and risk factors for colorectal neoplasia in a self-selected Vietnamese screening cohort undergoing self-funded colonoscopy
Source: PLoS One. 2026 Jul 13;21(7):e0352998. doi: 10.1371/journal.pone.0352998 (PMC13362143; doi:10.1371/journal.pone.0352998)
Supplement: S2 Table — (DOCX) [file pone.0352998.s002.docx]

**Supplementary Table 2: Exploratory multivariable logistic regression analysis of factors associated with colorectal neoplasia and advanced colorectal neoplasia**

| Risk factors | Overall colorectal neoplasia | | | Advanced colorectal neoplasia | | |
| --- | --- | --- | --- | --- | --- | --- |
|  | OR | 95% CI | p value | OR | 95% CI | p value |
| Male | 1.15 | 0.71-1.86 | 0.575 | 1.44 | 0.70-2.97 | 0.322 |
| Age ≥ 40 | 2.79 | 1.40- 5.56 | 0.004 | 5.20 | 1.22-22.11 | 0.025 |
| BMI ≥ 23 | 1.92 | 1.32-2.79 | 0.001 | 1.62 | 0.91-2.88 | 0.102 |
| Smoking | 1.32 | 0.78-2.23 | 0.307 | 0.75 | 0.35-1.61 | 0.461 |
| Alcohol consumption | 1.47 | 0.91-2.39 | 0.117 | 1.58 | 0.77-3.23 | 0.212 |
| Family history of CRC | 2.29 | 1.29-4.05 | 0.005 | 2.01 | 0.92-4.40 | 0.082 |

*OR: odds ratio, CI: confidence interval, BMI: Body mass index, CRC: Colorectal cancer*
