# Supplementary material for: Prevalence and risk factors for colorectal neoplasia in a self-selected Vietnamese screening cohort undergoing self-funded colonoscopy
Source: PLoS One. 2026 Jul 13;21(7):e0352998. doi: 10.1371/journal.pone.0352998 (PMC13362143; doi:10.1371/journal.pone.0352998)
Supplement: S3 Table — (DOCX) [file pone.0352998.s003.docx]

**Supplementary Table 3. E-values for significant factors associated with colorectal neoplasia in the primary multivariable analysis**

| Risk factors | Adjusted Odds Ratio (95% CI)* | E-value for point estimate | E-value for lower bound of 95% CI |
| --- | --- | --- | --- |
| Age (per 10-year increment) | 1.76 (1.47 – 2.11) | 1.98 | 1.72 |
| BMI ≥ 23 kg/m² | 1.70 (1.16 – 2.50) | 1.93 | 1.37 |
| Alcohol consumption | 1.83 (1.10 – 3.04) | 2.04 | 1.28 |
| Family history of CRC | 2.43 (1.36 – 4.37) | 2.49 | 1.61 |

*OR: odds ratio, CI: confidence interval, BMI: Body mass index, CRC: Colorectal cancer*.


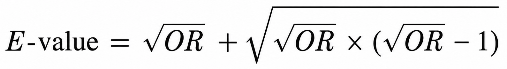
E-value was calculated with the following formula for colorectal neoplasia prevalence > 15%:
