# Supplementary material for: Prevalence and risk factors for colorectal neoplasia in a self-selected Vietnamese screening cohort undergoing self-funded colonoscopy
Source: PLoS One. 2026 Jul 13;21(7):e0352998. doi: 10.1371/journal.pone.0352998 (PMC13362143; doi:10.1371/journal.pone.0352998)
Supplement: Attachment — Submitted filename: Response - Plos One - R3 FINAL.docx [file pone.0352998.s009.docx]

May 03, 2026

Dear Editor,

We would like to thank you very much for your kindness in allowing us to revise our manuscript. We also thank the reviewers for their constructive comments. The manuscript has been revised accordingly, and the revised sections in the resubmitted manuscript have been highlighted in blue. We would like to respond to your comments as follows.

**Response to Reviewer 1**

**Comment 1. Severe selection bias undermines generalizability (unchanged from prior review).**

*The study recruits from a single private polyclinic in Ho Chi Minh City, where participants pay out-of-pocket for colonoscopy. These individuals are likely wealthier, more health-conscious, and have different risk profiles than the general Vietnamese population. The authors have added cautionary language to the conclusion, but this does not fix the fundamental design limitation. For a high-impact journal, a single-center, self-selected cohort cannot support meaningful prevalence estimates for the broader population – even as a “hypothesis-generating” study, the bias is too large.*

**Reply:**

Thank you very much for your comment. We fully agree that our study population represents a self-selected, screening-attending cohort from a private clinical setting, and therefore differs from the general Vietnamese population in terms of socioeconomic and health-seeking characteristics. However, we respectfully note that our primary objective was not to estimate population-level prevalence, but rather to describe the burden and associated risk factors of colorectal neoplasia within this increasingly common real-world screening context in Vietnam, where organized population-based screening programs are not yet established.

We have further revised the Discussion to explicitly emphasize that our findings are specific to this screening-attending population and should not be generalized to the average-risk population (page 18, lines 343-346).

“Importantly, our study was not designed to provide population-level prevalence estimates, but rather to describe the burden and associated risk factors of CRN within a real-world screening-attending population, which is common in Vietnam in the absence of organized national screening programs.”

**Comment 2. Post-hoc age dichotomization is a serious methodological flaw**

*The authors dichotomized age at ≥40 years for multivariable analysis without pre-specification, then used the data to justify this cutoff. This is a classic example of data-driven dichotomization that inflates Type I error and yields unstable estimates. The correct approach would have been to model age flexibly (splines, fractional polynomials) or use clinically justified cutoffs from external literature. The authors’ stated reason – avoiding collinearity and preserving power – does not excuse the post-hoc decision.*

**Reply:**

We thank the reviewer for this important methodological comment. We agree that dichotomization of continuous variables may introduce limitations, particularly if interpreted as data-driven. We would like to clarify, however, that the selection of an age threshold of ≥40 years in our analysis was informed by prior evidence and regional clinical context, rather than being derived solely from our dataset. Specifically, previous studies in Vietnam have reported a substantial proportion of early-onset colorectal cancer, suggesting that colorectal neoplasia may occur at a younger age compared with Western populations [4]. In addition, screening practices in some Asian countries, such as Japan, begin at age 40 [10]. Furthermore, a large prospective colonoscopy study in asymptomatic Korean adults—representing a screening-attending population similar to our study setting—demonstrated that colorectal neoplasia occurs across a broad age range, including individuals younger than 50 years [28]. Taken together, these findings provide a clinical and epidemiological rationale for exploring a lower age threshold in this context, rather than relying exclusively on conventional cutoffs derived from Western populations.

To address the reviewer’s concern, we have revised the Methods and Discussion sections to clarify this rationale and have incorporated supporting evidence from studies in Vietnam, Japan, and Korea (pages 12-13, lines 217-221; page 15, lines 270-275; and page 16, lines 289-293).

“We categorized age using a cutoff of ≥ 40 years for multivariable analysis. This threshold was selected based on prior evidence suggesting an earlier onset of CRN in Asian populations, including data from Vietnam [4], as well as regional screening practices and guidelines [10]. This approach also facilitated a simplified and interpretable model.”

“Evidence from Vietnam has demonstrated a substantial proportion of early-onset RC [4], while screening practices in some Asian countries, such as Japan, begin at age 40 [10]. In addition, a large prospective colonoscopy study in asymptomatic Korean adults—reflecting a screening-attending population—has shown that CRN may occur before the age of 50 [28]. Together, these findings support the rationale for exploring a lower age threshold in this context.”

“Therefore, these data suggest a potential burden of CRC in younger adults within screening-attending populations. These observations highlight the need for further investigation into age-specific risk stratification in this context. However, this observation should be interpreted as hypothesis-generating, and confirmation from large-scale, population-based studies is required before informing national policy.”

References:

4. Quach DT, Nguyen OT. Clinical, endoscopic and pathogical characteristics of early-onset colorectal cancer in Vietnamese. Asian Pac J Cancer Prev. 2012;13(5):1767-70. Epub 2012/08/21. doi: 10.7314/apjcp.2012.13.5.1767. PubMed PMID: 22901119.

10. Saito Y, Oka S, Kawamura T, Shimoda R, Sekiguchi M, Tamai N, et al. Colonoscopy screening and surveillance guidelines. Dig Endosc. 2021;33(4):486-519. Epub 2021/03/14. doi: 10.1111/den.13972. PubMed PMID: 33713493.

28. Park HW, Byeon JS, Yang SK, Kim HS, Kim WH, Kim TI, et al. Colorectal Neoplasm in Asymptomatic Average-risk Koreans: The KASID Prospective Multicenter Colonoscopy Survey. Gut Liver. 2009;3(1):35-40. Epub 2009/03/01. doi: 10.5009/gnl.2009.3.1.35. PubMed PMID: 20479899; PubMed Central PMCID: PMCPMC2871563.

**Comment 3. Inadequate control for confounding**

*The multivariable model adjusts for only 6 variables (age, sex, BMI, family history, smoking, alcohol). Numerous known CRN risk factors are omitted: diabetes, physical inactivity, red/processed meat intake, NSAID use, dietary fiber, and socioeconomic status. Given the self-selected nature of the cohort, residual confounding is likely substantial. The authors do not provide any sensitivity analyses (e.g., E-values, propensity score approaches) to assess robustness*.

**Reply:**

We thank the reviewer for this important comment. We agree that residual confounding cannot be completely excluded in our study, given its observational design and the limited availability of certain variables such as dietary factors, physical activity, metabolic comorbidities (e.g., diabetes), and socioeconomic status. However, we included key established risk factors that have been consistently reported in the literature and were feasible to collect in routine clinical practice. Notably, a large prospective multinational study in asymptomatic Asian populations identified advancing age, male sex, and family history of colorectal cancer as major risk factors for colorectal neoplasia, which are consistent with the variables included in our model [25].

To address the reviewer’s concern, we have expanded the Discussion and Limitation section to more explicitly acknowledge the potential impact of unmeasured confounders and to clarify that the observed associations should be interpreted with caution. We agree that future studies incorporating more comprehensive exposure assessment and additional lifestyle and metabolic variables would be valuable to further refine risk estimation (page 15, lines 277-280 and page 19, lines 360-362).

“These findings are consistent with those from a large prospective multinational study in asymptomatic Asian populations, which identified advanced age, male sex, and family history of CRC as key risk factors for CRN [25].”

“Therefore, the observed associations should be interpreted with caution, as unmeasured confounding factors may have influenced the results.”

References:

25. Byeon JS, Yang SK, Kim TI, Kim WH, Lau JY, Leung WK, et al. Colorectal neoplasm in asymptomatic Asians: a prospective multinational multicenter colonoscopy survey. Gastrointest Endosc. 2007;65(7):1015-22. Epub 2007/05/29. doi: 10.1016/j.gie.2006.12.065. PubMed PMID: 17531636.

**Comment 4. Underpowered analysis for advanced CRN**

*Only 64 participants had advanced CRN. The multivariable model for this outcome includes 6 predictors, yielding an events-per-variable ratio of ~10.7 – at the absolute minimum acceptable threshold. The resulting confidence intervals are very wide (e.g., age OR 5.20, 95% CI 1.22–22.11), and the null findings for BMI and family history should not be interpreted as evidence of no association. The authors acknowledge this limitation, but the analysis remains exploratory and should not be presented as definitive.*

**Reply:**

Thank you very much for your comment. We agree that the number of advanced CRN cases was relatively limited, which may affect the precision of multivariable estimates. We have emphasized in the revised manuscript that analyses related to advanced CRN should be considered exploratory and that the absence of statistical significance should not be interpreted as evidence of no association (pages 17-18, lines 334-336).

“Accordingly, the absence of statistical significance for certain variables should not be interpreted as evidence of no association, but rather as a reflection of limited statistical power.”

**Comment 5. Unvalidated definition of “asymptomatic”**

*Asymptomatic status was determined by unstructured clinical interviews, not by validated instruments (e.g., Rome criteria). No information is provided on inter-rater reliability or the specific questions asked. Recall bias is likely, and some participants may have had mild or intermittent symptoms that were not captured. The prevalence estimate could be meaningfully affected by misclassification.*

**Reply:**

Thank you very much for your comment. We acknowledge that asymptomatic status was not defined using a formally validated instrument. This approach reflects routine clinical practice in screening settings, where symptom assessment is typically conducted through physician-led interviews. We have addressed the potential for misclassification in the limitation section of the revised manuscript (page 18, lines 351-355).

“Second, although asymptomatic status was determined through structured clinical assessment by experienced gastroenterologists—as is typical in routine screening practice—it was not based on a formally validated instrument, which may introduce some degree of symptom misclassification and potentially influence prevalence estimates.”

**Comment 6. NICE type I discordance highlights quality issue**

*The authors note that 15.3% of lesions were endoscopically classified as NICE type I (suggesting hyperplastic) but were histologically confirmed as neoplasia. This discordance rate is higher than reported in high-quality optical diagnosis studies. It raises questions about the endoscopists’ real-time diagnostic accuracy and whether all participants received equivalent quality examination. The authors add a sentence acknowledging this as a limitation, but the high discordance should have triggered a more thorough quality control review.*

**Reply:**

We thank you for this important observation. We agree that the observed discordance between NICE classification and histopathological findings may raise concerns regarding real-time optical diagnosis. However, this level of discordance is consistent with known limitations of optical diagnosis, particularly for small or subtle lesions in routine clinical practice. Importantly, all colonoscopies in our study were performed by experienced endoscopists who met established quality indicators, including an adenoma detection rate exceeding 30%, suggesting that overall examination quality was maintained. In addition, lesions suspected to be non-neoplastic were generally resected and subjected to histopathological evaluation, which may reduce the potential impact of optical misclassification on study outcomes.

To address the reviewer’s concern, we have expanded the Discussion to clarify that this discordance likely reflects inherent limitations of optical diagnosis rather than a systematic quality issue, and that the use of histopathological confirmation mitigates its potential impact on study outcomes (page 15, lines 265-269).

“However, all colonoscopies were performed by experienced endoscopists with established quality indicators (adenoma detection rate >30%), suggesting that overall examination quality was maintained. In addition, lesions suspected to be non-neoplastic were generally resected and histologically examined, which may mitigate the impact of optical misclassification on study outcomes.”

Once again, we would like to thank you for your time, and we greatly appreciate your insights that helped us refine the manuscript.

With the warmest regards,

On behalf of the authors

Duc Quach, MD; PhD

Department of Internal Medicine and Gastro-Hepato Integrated Research Team (GHIRT-002.TCM2025), School of Medicine, University of Medicine and Pharmacy at Ho Chi Minh City, Vietnam

217 Hong Bang Street, Cho Lon Ward, Ho Chi Minh City, Vietnam

Tel: +84-91-808-025

Email: [drquachtd@ump.edu.vn](mailto:drquachtd@ump.edu.vn)
