# Supplementary material for: Prevalence and risk factors for colorectal neoplasia in a self-selected Vietnamese screening cohort undergoing self-funded colonoscopy
Source: PLoS One. 2026 Jul 13;21(7):e0352998. doi: 10.1371/journal.pone.0352998 (PMC13362143; doi:10.1371/journal.pone.0352998)
Supplement: Attachment — Submitted filename: point-to-ponit reply.docx [file pone.0352998.s007.docx]

March 03, 2026

Dear Editor,

We would like to thank you very much for your kindness in allowing us to revise our manuscript. We also thank the reviewers for their constructive comments. The manuscript has been revised accordingly, and the revised sections in the resubmitted manuscript have been highlighted in blue. We would like to respond to your comments as follows.

**Response to Reviewer 1**

1. **Introduction**

**Comment.** *Consider adding a sentence explicitly mentioning the lack of data in asymptomatic Vietnamese individuals to further justify the study's focus.*

**Reply:**

We sincerely thank you for your valuable suggestion. We have revised the Introduction section accordingly to explicitly highlight the lack of data in asymptomatic Vietnamese individuals (page 5, lines 85-89).

“Previous research on CRN in Vietnam has predominantly focused on symptomatic patients, leaving a substantial gap in evidence regarding the age-stratified prevalence—particularly among adults under 40 years—and the risk factors of CRN in asymptomatic Vietnamese individuals.”

2. **Materials and Methods**

**Comment 1*.*** *Line 105-110 (Definitions): The definition for "Non-alcohol consumption" is slightly awkward ("never drinking or drinking alcohol once per month or less"). Consider rephrasing to "Alcohol consumption was defined as drinking more than once per month."*

**Reply:**

Thank you very much for your helpful suggestion. We have revised the definition of alcohol consumption in the Methods section accordingly (page 7, lines 118-119).

“Alcohol consumption was defined as drinking more than once per month.”

**Comment 2*.*** *Line 127-128: "...analysed. The participating endoscopists directly evaluated..." Consider combining: "...recorded and analyzed by the participating endoscopists using standardized data collection sheets."*

**Reply:**

Thank you very much for your constructive comment. In response, we have revised the Methods section accordingly (page 7, lines 137-138).

“The morphology, location, and size of all the detected polyps were recorded and analysed by the participating endoscopists using standardized data collection sheets.”

**Comment 3*.*** *Line 156: "SPSS (version 23; SPSS Inc, Chicago, IL)" - It's more standard to use "IBM SPSS Statistics for Windows, Version 23.0."*

**Reply:**

We appreciate the reviewer’s suggestion and have updated the software description in the statistical analysis section accordingly (page 9, lines 169-170).

“Statistical analyses were performed with IBM SPSS Statistics for Windows, version 23.0.”

3. **Results**

**Comment 1*.*** *Critical Error in Table 1: There is a significant formatting/presentation error in the BMI rows. The numbers under "No colorectal neoplasia" and "Colorectal neoplasia" columns for BMI ≥23 and <23 do not align correctly with the headers and total N. The percentages (55.4%, 44.6%, 70.3%, 29.7%) seem to be swapped between the "No CRN" and "CRN" groups or are miscalculated. This must be corrected. The p-value (0.021) suggests a difference, but the displayed numbers are confusing.*

**Reply:**

Thank you very much for pointing out this error. We sincerely apologize for the typographical errors in the BMI rows of Table 1, which occurred during the final formatting of the manuscript. We have corrected these figures in the revised Table 1 and have thoroughly rechecked the whole table against our raw data. We would like to clarify that these were reporting mistakes only; therefore, the independent risk factors, odds ratios, and p-values of the multivariable analysis are accurate and remain unchanged.

**Comment 2*.*** *Table 1 Footnote: The footnote says p-values for age groups represent comparisons with the reference group (< 40 years). This is typically indicated in the table with superscripts (e.g., a, b, c). Add these superscripts to the p-value column for clarity.*

**Reply:**

We would like to thank you for your constructive comment. We have added superscripts (e.g., a, b, c) to the p-value column in Table 1 to clearly indicate that these values represent comparisons between each age group and the reference group (< 40 years). The footnote has also been revised to include these indicators.

**Table 1. Characteristics of participants with and without colorectal neoplasia**

| Characteristics | Total  (n, %)  N = 714 | No colorectal neoplasia (n, %)  N = 527 | Colorectal neoplasia  (n, %)  N = 187 | p-value |
| --- | --- | --- | --- | --- |
| Sex |  |  |  |  |
| Male | 290 (40.6) | 194 (36.8) | 96 (51.3) | 0.001 |
| Female | 424 (59.4) | 333 (63.2) | 91 (48.7) |  |
| Age |  |  |  |  |
| < 40 | 83 (11.6) | 72 (13.7) | 11 (5.9) |  |
| 40-49 | 221 (31.0) | 175 (33.2) | 46 (24.6) | 0.157 ^a^ |
| 50-59 | 238 (33.3) | 177 (33.6) | 61 (32.6) | 0.134 ^b^ |
| 60-69 | 146 (20.4) | 88 (16.7) | 58 (31.0) | 0.004 ^c^ |
| ≥ 70 | 26 (3.6) | 15 (2.8) | 11 (5.9) | 0.026 ^d^ |
| BMI (kg/m^2^) |  |  |  |  |
| ≥ 23 | 405 (56.7) | 274 (52.0) | 131 (70.1) | < 0.001 |
| < 23 | 309 (43.3) | 253 (48.0) | 56 (29.9) |  |
| Smoking |  |  |  |  |
| Yes | 105 (14.7) | 64 (12.1) | 41 (21.9) | 0.001 |
| No | 609 (85.3) | 463 (87.9) | 146 (78.1) |  |
| Alcohol consumption |  |  |  |  |
| Yes | 208 (29.1) | 132 (25.0) | 76 (40.6) | < 0.001 |
| No | 506 (70.9) | 395 (75.0) | 111 (59.4) |  |
| Family history of CRC |  |  |  |  |
| Yes | 60 (8.4) | 35 (6.6) | 25 (13.4) | 0.04 |
| No | 654 (91.6) | 492 (93.4) | 162 (86.6) |  |
| *OR: Odds ratio, CI: Confidence interval, BMI: Body mass index, CRC: Colorectal cancer*  *^a, b, c, d^ p-values represent comparisons of the 40-49, 50-59, 60-69, and* ≥ *70 years age groups, respectively, with the reference group (< 40 years)* | | | | |

**Comment 3*.*** *Lines 203-205: The rationale for using age ≥40 as a binary cut-off is very good and should be kept. It strengthens the analysis.*

**Reply:**

Thank you very much for this encouraging and supportive comment. We appreciate the reviewer’s assessment that the age ≥ 40 cut-off strengthens the analysis; therefore, we have retained this rationale in the revised manuscript.

4. **Discussion**

**Comment 1*.*** *Lines 236-240: The paragraph comparing findings to a study on IBS patients is slightly confusing. It states the high proportion of advanced CRN "may be attributed to the inclusion of symptomatic patients," which contrasts with your study's focus on asymptomatic individuals. This comparison may not be the most relevant. Consider focusing more on comparisons with other asymptomatic screening studies.*

**Reply:**

We sincerely thank the reviewer for this insightful comment. We acknowledge that comparisons with a study of symptomatic IBS patients may be less directly relevant to our asymptomatic screening population. In the revised manuscript, we have therefore prioritized comparisons with asymptomatic screening studies from the Asia-Pacific region (pages 13-14, lines 234-236 and pages 14, lines 237-240).

Given the absence of published data on CRN prevalence among asymptomatic individuals in Vietnam, we have referred to the IBS cohort solely as contextual national background information. As kindly suggested, we have clarified in the revised manuscript that this comparison does not represent a directly comparable screening population (page 14, lines 246-248).

“The prevalence of overall CRN and advanced CRN in asymptomatic subjects from Asia Pacific studies ranged from 19.2-36.0%, and from 1.7-15.4%, respectively [22-24].”

“Another prospective multinational multicenter survey of asymptomatic subjects over 16 years old in 11 Asian cities reported that the prevalence of CRN and advanced CRN were 18.5% and 4.5%, respectively [25].”

“Currently, no published data are available on CRN prevalence among asymptomatic individuals in Vietnam. The only available local data derived from a cohort of patients with irritable bowel syndrome, with rates of 9.1% and 6.9%, respectively [26].”

**Comment 2*.*** *Limitations Section (Lines 294-300): Acknowledges key limitations appropriately. Consider adding that the single-center design, while a limitation, also ensured standardized, high-quality colonoscopy procedures, which is a strength for internal validity.*

**Reply:**

Thank you very much for your helpful suggestion. We totally agree that while the single-center design limits generalizability, it offers procedural consistency. We have incorporated this point into the revised manuscript to strengthen the internal validity of our study (page 17, lines 331-333).

“Nonetheless, the single-center setting allowed for standardized colonoscopy procedures and centralized histopathological evaluation, which may enhance internal consistency.”

**Response to Reviewer 2**

1. **Title**

**Comment 1:** *Scope mismatch / definitional ambiguity ("colorectal neoplasia): The title refers to the lesions that involve the spectrum of CRC on a broad scale, and the manuscript has defined CRN as including adenomas/SSL/CRC and subsequently has reported the advanced CRN separately. There needs to be more specific information (e.g., adenoma/serrated lesions and cancer) or an unequivocated definition in the title/abstract. This is important as it can mislead readers and indexers (to think that the results are cancer or precancer) thereby affecting interpretability and comparability.*

**Reply:**

We sincerely thank the reviewer for this insightful comment regarding the potential for definitional ambiguity of the term colorectal neoplasia (CRN). As you kindly suggested, we have added an unequivocal definition of CRN in the abstract of the revised manuscript to encompass the broad clinical spectrum of lesions, ranging from precancerous precursors to invasive carcinoma (page 2, lines 25-26). We have also ensured that the distinction between overall CRN and advanced CRN is consistently maintained throughout the manuscript to improve clarity and interpretability.

“CRN was defined as the presence of adenoma, sessile serrated lesions, or colorectal cancer.”

**Comment 2:** *Asymptomatic" not operationalized in title: Title claims asymptomatic screening population, but "asymptomatic" is defined by absence of lower GI + alarm symptoms and includes self-paid voluntary checkups (selection)."This also implies that this is an average risk screening population; most especially if it's a self-selected cohort, external validity is limited.*

**Reply:**

Thank you very much for your valuable comment. We agree that a self-selected, self-paid cohort may limit the external validity and generalizability of the findings compared to a population-based average-risk group. To address this, we have updated the title to "Prevalence and risk factors for colorectal neoplasia in asymptomatic Vietnamese adults undergoing self-funded screening colonoscopy" to accurately reflect the voluntary, self-paid status of the participants.

**Comment 3:** *The short title repeats the long title with little shortening. short titles should enhance discoverability and clarity on the layout of journal pages/metadata.*

**Reply:**

We appreciate the reviewer’s suggestion to improve the short title for better clarity and discoverability. We have revised the short title to be more concise while retaining the study's core focus, as you kindly suggested.

"Colorectal neoplasia in asymptomatic Vietnamese adults"

2. **Abstract**

**Comment 1:** *Study design & sampling frame not sufficiently explicit: Abstract says "cross-sectional" and "voluntary screening colonoscopy," but does not flag this is a single-center, self-paid, self-selected cohort. This information matters because selection bias can lead to overestimating prevalence and cause distortions in the estimation of risk factors; and readers need this upfront.*

**Reply:**

Thank you very much for your kind suggestion. We agree that explicitly identifying the cohort as a single-center, self-selected, and self-paid group in the Abstract is essential for readers to accurately interpret the prevalence and risk factors. Consequently, we have revised the Abstract to include this information (page 2, lines 23-25).

"This was a prospective, cross-sectional, single-center study. Participants were consecutively recruited from asymptomatic individuals who were self-selected to undergo self-funded screening colonoscopy."

**Comment 2:** *The choices of predictors in the risk factor model are not sufficiently transparent: The abstract refers to age (approximately 40), BMI (approximately 23), and family history as the independent predictors, but does not state the covariates that were considered, as well as the approaches to control the potential confounding factors (e.g., sex, smoking, alcohol consumption).*

**Reply:**

We thank the reviewer for this important comment. We have revised the Abstract to clearly specify that age, sex, BMI, family history of colorectal cancer, smoking status, and alcohol consumption were included in the multivariable logistic regression model to adjust for potential confounding (page 2, lines 29-31).

“Multivariable logistic regression was performed to identify independent risk factors for CRN, adjusting for age, sex, BMI, family history of colorectal cancer, smoking status, and alcohol consumption.”

**Comment 3:** *Definitions of outcomes not mentioned: CRN and advanced CRN prevalence are provided, but there is no abstract summary of their definition (adenoma/SSL/CRC; advanced criteria). Outcome definition propels prevalence magnitude and cross study comparisons.*

**Reply:**

Thank you very much for your valuable comment on the need for clear definitions of CRN and advanced CRN. To address this, we have incorporated a summary of the criteria for CRN and advanced CRN into the Abstract of the revised manuscript (page 2, lines 25-28).

“CRN was defined as the presence of adenoma, sessile serrated lesions, or colorectal cancer. Advanced CRN included adenoma ≥ 1 cm, with villous features or high-grade dysplasia; sessile serrated lesion ≥ 1 cm or with dysplasia; traditional serrated adenoma; or colorectal cancer."

3. **Keywords**

**Comment**: *Keywords are too generic and do not include important indexing terms: Current list does not include "screening colonoscopy," "adenoma," "advanced neoplasia," "serrated lesions," "asymptomatic," and "cross-sectional." Discoverability and appropriate indexing in the databases will be minimized, reducing reach and the chances of citation*

**Reply:**

Thank you very much for this valuable suggestion. In the revised manuscript, we have updated the keyword section to include more specific and relevant indexing terms, including “screening colonoscopy,” “adenoma,” “advanced neoplasia,” “serrated lesions,” “asymptomatic,” and “cross-sectional” (page 3, lines 42-43).

4. **Introduction**

**Comment 1.** *Rationale and knowledge gap are not well specified- Gap is stated ("limited data in asymptomatic") but not sharpened into a testable, novel contribution: The intro repeats that Vietnam lacks screening infrastructure and that asymptomatic data are limited, but does not specify what is uniquely unknown (e.g., age-stratified prevalence including <40, advanced CRN proportion, or risk-factor performance in a self-paid cohort). It is important for the question to be well defined so the reader can assess whether or not the methods and results actually address the question.*

**Reply:**

We appreciate the reviewer's invaluable comment and have revised the introduction section accordingly (page 5, lines 85-89).

“Previous research on CRN in Vietnam has predominantly focused on symptomatic patients, leaving a substantial gap in evidence regarding the age-stratified prevalence—particularly among adults under 40 years—and the risk factors of CRN in asymptomatic Vietnamese individuals.”

**Comment 2.** *Important construct ("asymptomatic") is not introduced with sufficient precision- The intro presents CRC as "largely asymptomatic until advanced" and calls for screening "even in asymptomatic individuals", but fails to preview the manuscript's operational definition of asymptomatic (no lower GI + no alarm symptoms) and the implications of voluntary/self-paid selection. This is important because the significance of "asymptomatic" has a strong influence on generalizability and risk interpretation.*

**Reply:**

Thank you very much for your insightful comment. In response, we have revised the manuscript to clearly define the term “asymptomatic” and to explicitly state the voluntary, self-paid characteristics of our study population (page 4, lines 62-64; page 6, lines 101-105; and page 17, lines 325-331).

“Therefore, CRC screening for early detection plays a critical role, including among asymptomatic individuals, defined as those without lower gastrointestinal symptoms or alarm features.”

“The eligibility criteria for this study were: age ≥18 years and asymptomatic status, defined as the absence of lower intestinal symptoms (including recent changes in bowel habits, abdominal pain, or hematochezia) and the absence of alarm features, such as anemia or unintentional weight loss exceeding 5% of body weight within 6-12 months.”

“First, this was a single-center study involving individuals who elected to undergo self-funded screening colonoscopy. This self-selected recruitment framework may introduce selection bias, as participants may differ from the general population in terms of health awareness, socioeconomic status, and underlying risk profile. Consequently, the estimated prevalence and observed associations should be interpreted with caution, and the findings may not be fully generalizable to the broader average-risk Vietnamese population.”

**Comment 3.** *Internal logic for the age threshold is not consistent. The introduction claims that there is an increase in early-onset colorectal cancer (CRC) and cites age groups (40, 45, 50); however, no explanation is given in the introduction for including individuals aged 18 and older until the Methods section. Without an explicit a priori rationale, inclusion of very young adults can appear to be a post hoc inclusion and may serve to dilute screening-relevant inference.*

**Reply:**

We sincerely thank the reviewer for this important and thoughtful comment. We have revised the Introduction to clarify a priori rationale for including individuals aged ≥ 18 years. Specifically, we now state that no nationally endorsed colorectal cancer screening guideline has been formally implemented in Vietnam, and that screening remains largely opportunistic and self-funded, with colonoscopy performed across a broad adult population in routine practice. Accordingly, inclusion of adults aged ≥ 18 years was predefined to reflect the actual screening population. The revised text is provided below (pages 5-6, lines 89-95).

“To date, no nationally endorsed CRC screening guideline has been formally implemented in Vietnam. Screening is largely opportunistic and self-funded, with colonoscopy performed across a broad adult population in routine practice. Robust data from such settings are needed to better inform evidence-based screening policies. Consequently, this study aimed to determine the prevalence of CRN in asymptomatic Vietnamese adults undergoing self-funded screening colonoscopy and to identify significant risk factors associated with these lesions.”

5. **Methods**

**Comment 1.** *Study design & setting: Selection bias not handled/mitigated- The research is introduced as a prospective cross-sectional research on voluntary, self-paid screening colonoscopy participants in one private clinic. This is a recruitment frame with a high likelihood of selection bias (health-seeking, greater SES, risk-aware people), and which has the potential to overstate prevalence estimates and mislead risk factor associations unless carefully mitigated.*

**Reply:**

We sincerely thank the reviewer for this important comment. We have revised the Methods section to more clearly describe the recruitment framework, emphasizing that participants were consecutively enrolled asymptomatic adults who elected to undergo self-funded screening colonoscopy as part of routine health examinations at a single center. This clarification was added to transparently reflect the self-selected nature of the cohort (page 6, lines 98-101).

“This was a prospective, cross-sectional, single-center study. Participants were consecutively recruited from asymptomatic adults who elected to undergo self-funded screening colonoscopy as part of routine health examinations at Dai Phuoc Polyclinic, Ho Chi Minh City, Vietnam, from 10/04/2022 to 31/03/2023.”

In addition, we have strengthened the Limitations section to explicitly acknowledge the potential for selection bias arising from this self-selected, self-funded recruitment framework and to clarify that both prevalence estimates and identified risk associations should be interpreted with caution regarding generalizability to the broader Vietnamese population (page 17, lines 325-331).

“First, this was a single-center study involving individuals who elected to undergo self-funded screening colonoscopy. This self-selected recruitment framework may introduce selection bias, as participants may differ from the general population in terms of health awareness, socioeconomic status, and underlying risk profile. Consequently, the estimated prevalence and observed associations should be interpreted with caution, and the findings may not be fully generalizable to the broader average-risk Vietnamese population.”

**Comment 2.** "*Asymptomatic" definition & ascertainment are under specified. “Asymptomatic” is characterized by the lack of distinctive lower intestinal symptoms and alarm symptoms but Methods fails to indicate how symptoms/anemia/weight loss were evaluated (structured questionnaire vs chart review vs labs), by whom, and at what time in relation to colonoscopy.This is significant because misclassification, especially marginally symptomatic or inadequately screened people, can distort prevalence and relationships.*

**Reply:**

We sincerely thank the reviewer for pointing out the need to clarify the ascertainment of asymptomatic cases. We have revised the Methods section to clarify how asymptomatic status was ascertained. (page 6, lines 105-111).

“Asymptomatic status was determined prior to colonoscopy through standardized pre-procedural clinical evaluation conducted by experienced gastroenterologists. Lower gastrointestinal symptoms and alarm features were assessed using structured clinical interviews and physical examination. All participants underwent routine pre-procedural laboratory testing, including complete blood count, and hemoglobin levels were reviewed to identify anemia.”

**Comment 3.** *Eligibility/exclusion choices may cause systematic changes in prevalence estimates- Excluding patients with inadequate bowel prep (Boston score thresholds), incomplete colonoscopy, and withdrawal time <6 minutes appropriate for quality control but Methods don't say if the excluded patients were different from the included patients (no comparison table) and how many were excluded because of what reason (some of this appears later in Results) Exclusions associated with patient factors (age/comorbidity/constipation) can introduce bias in the estimates of prevalence/risk factors.*

**Reply:**

Thank you very much for your thoughtful comment. We agree that exclusion criteria may introduce bias, particularly if the number of excluded participants is substantial. However, as detailed in our study flow diagram (Figure 1), the number of participants excluded for quality-control reasons was small (12/735). Given this very small proportion of exclusions, their impact on overall prevalence estimates and risk factor associations is likely limited. We have addressed this point in the Results and Discussion sections of the revised manuscript (page 9, lines 184-187 and page 14, lines 257-259).

“Among 735 eligible cases, 3 were excluded due to incomplete colonoscopy, 6 due to inadequate bowel preparation with a total score of < 6 or a segment score of < 2 according to the Boston Bowel Preparation Scale, and 3 due to withdrawal time < 6 minutes, resulting in a cecal intubation rate of 99.6%.”

“In our study, the number of participants excluded for quality-control reasons was small (12/735). Given this very small proportion of exclusions, their impact on overall prevalence estimates and risk factor associations is likely limited.”

**Comment 4.** *Exposure (risk factor) measurement is too gross and incomplete - Smoking is defined as “currently smoking or ever smoked,” and alcohol as “never/≤1× per month” vs others, with no quantity/duration, no pack-years, no standard-drink metric, and no dietary/physical activity/diabetes/dyslipidemia capture (acknowledged only in Discussion, not planned in Methods). This matters because crude exposure measurement increases residual confounding and can wash out true associations or create spurious ones.*

**Reply:**

Thank you very much for your insightful comment. We acknowledge that smoking and alcohol consumption were categorized using simplified definitions. In addition, other metabolic and lifestyle factors such as diabetes, dyslipidemia, physical activity, and dietary patterns were not systematically captured. We recognize that these limitations may contribute to residual confounding and reduced precision in estimating risk associations. This has now been explicitly acknowledged and elaborated in the limitation section of the revised manuscript, where we emphasize the need for future studies with more comprehensive and quantitative exposure assessment (page 18, lines 336-344).

“Third, some factors potentially associated with CRN, such as diabetes mellitus, dyslipidemia, physical inactivity, and dietary factors including high red meat intake and low fiber consumption, were not considered in our study. Furthermore, smoking and alcohol consumption were categorized using simplified definitions and were not evaluated quantitatively. These limitations may introduce residual confounding and reduce the precision of risk estimates. Future studies incorporating more detailed exposure assessment and broader metabolic profiling are warranted to better delineate these associations.”

**Comment 5.** *Outcomes are defined, but key procedural/ histology safeguards missing- CRN/advanced CRN definitions are clear, however, Methods do not state if pathologists were blinded to clinical risk factors/endoscopic impression, whether there was dual reading or how disagreements were resolved. Lack of blinding/standardization can cause classification bias especially for serrated lesion/dysplasia calls.*

**Reply:**

We appreciate the reviewer’s emphasis on the importance of histopathological safeguards. Accordingly, we have updated the Methods section to clarify the pathology protocol (page 8, lines 154-159).

“Histopathological evaluation of all resected specimens was performed by expert gastrointestinal pathologists at the Department of Histology-Embryology and Pathology, University of Medicine and Pharmacy at Ho Chi Minh City. Pathologists were blinded to the patients' risk factor profiles. In cases of diagnostic uncertainty, slides were independently reviewed by a second senior pathologist, and discrepancies were resolved by consensus to minimize inter-observer variability.”

6. **Results**

**Comment 1.** *Prevalence reporting is not contextual in terms of uncertainty and age standard- The Results reflect CRN and an advanced CRN prevalence of 26.2% and 9.0%, respectively, without the reporting of 95% confidence intervals or age-standardised estimates, despite a significantly age-skewed sample (88.4% aged 40 years and above), In the absence of standardisation and certainty, the reported prevalence may be deceptive, and difficult to compare between different populations.*

**Reply:**

We sincerely thank the reviewer for the critical appraisal regarding the reporting of CRN and advanced CRN prevalence estimates. To address this, we have added two-sided 95% confidence intervals (CIs) for all prevalence estimates, calculated using the Clopper–Pearson exact method, in the revised manuscript (page 9, lines 177-178). Furthermore, we have performed age-standardization using the 2024 Vietnam Intercensal Population and Housing Survey as the reference population to ensure cross-population comparability (page 11, lines 200-205).

“The 95% confidence intervals (CIs) for prevalence rates were calculated using the Clopper-Pearson exact method.”

“Among the 714 asymptomatic subjects, 187 were diagnosed with CRN, yielding a crude prevalence of 26.2% (95% CI: 23.0%-29.6%) and an age-standardized prevalence of 21.3% based on the 2024 Vietnam Intercensal Population and Housing Survey [20]. Advanced CRN was detected in 64 patients, corresponding to a crude prevalence of 9.0% (95% CI: 7.0%-11.3%) and an age-standardized prevalence of 6.4%, of whom 2 patients (0.3%, 95% CI: 0.03%-1.01%) had adenocarcinoma.”

**Comment 2.** *Endoscopic characterization vs pathology discordance is flagged but not resolved- The manuscript points out a discrepancy between 15.3% of lesions that were NICE type 1 but "all were histologically confirmed as CRN." This suggests a possible problem of misclassification or quality of documentation, which can lead to lack of confidence in endoscopic evaluation and characterisation of the lesion.*

**Reply:**

We sincerely thank the reviewer for pointing out the discordance between endoscopic NICE classification and histology. Although 15.3% of lesions were classified endoscopically as NICE type I, all were confirmed as CRN on histopathology, which served as the reference standard. Such discrepancies between endoscopic appearance and histopathology have been reported in clinical practice, particularly for small lesions [27]. In our cohort, 53.8% of CRN were ≤ 5 mm, where optical differentiation between diminutive hyperplastic, serrated, and low-grade neoplastic lesions can be challenging. We have clarified this point in the revised manuscript (pages 14-15, lines 259-264).

“In addition, although 15.3% of lesions were endoscopically classified as NICE type I, all were histologically confirmed as CRN. There may be discrepancies between endoscopic appearance and pathological diagnosis, particularly in small lesions where optical differentiation between diminutive hyperplastic, serrated, and low-grade neoplastic lesions can be challenging [27].”

Regarding procedural quality, colonoscopy standards were rigorously maintained in our study. All participating endoscopists had adenoma detection rates exceeding 30% and had performed more than 3,000 colonoscopies over the last five years, reflecting adherence to established quality benchmarks. We have revised the Methods section to explicitly state this point (page 7, lines 131-134).

“All participating endoscopists had adenoma detection rates of more than 30% and had performed at least 3,000 colonoscopic procedures over the last five years, indicating adherence to established quality standards.”

*References:*

*27. Ishii T, Nishizawa T, Watanabe H, Sano M, Fujimoto A, Takahashi Y, et al. Efficacy of additional tissue sections for diminutive colorectal adenomas pathologically diagnosed as normal mucosa: a retrospective, cross-sectional study in Japan. Clin Endosc. 2025;58(4):577-85. Epub 2025/08/01. doi: 10.5946/ce.2024.265. PubMed PMID: 40746142; PubMed Central PMCID: PMCPMC12314616.*

**Comment 3.** *Results occasionally include interpretation that belongs in Discussion- The Results section speculates on reasons for null associations (e.g. "limited number" and "biological differences"). Results should be descriptive; interpretation here can look like narrative steering too and weakens objectivity.*

**Reply:**

Thank you very much for your helpful suggestion. We agree that interpretative statements should not be included in the Results section. Accordingly, we have revised the manuscript to remove speculative explanations from the Results and have relocated these interpretations to the Discussion section (pages 14-15, lines 257-264 and page 17, lines 318-324). The Results section now presents purely descriptive findings.

“In our study, the number of participants excluded for quality-control reasons was small (12/735). Given this very small proportion of exclusions, their impact on overall prevalence estimates and risk factor associations is likely limited. In addition, although 15.3% of lesions were endoscopically classified as NICE type I, all were histologically confirmed as CRN. There may be discrepancies between endoscopic appearance and pathological diagnosis, particularly in small lesions where optical differentiation between diminutive hyperplastic, serrated, and low-grade neoplastic lesions can be challenging [27].”

“While BMI ≥ 23 kg/m² and a first-degree relative with CRC were identified as independent risk factors for overall CRN, these associations did not reach statistical significance for advanced CRN. This lack of significance is likely attributable to the limited number of advanced CRN cases, which could potentially affect statistical power. Moreover, biological differences in the progression from non-advanced to advanced neoplasia may contribute to this finding, particularly in the context of early detection through screening colonoscopy.”

7. **Discussion**

**Comment 1.** *The generalizability is exaggerated with regard to self-selected, self-paid cohort. Discussion interprets prevalence and risk factors to be informative of wider screening prioritization and national level context although its sample is limited to one private clinic and to individuals who had voluntarily paid to undergo colonoscopy. This is very significant as selection bias can materially change the prevalence and predictor strength. overgeneralizing to mislead policy/clinical implications.*

**Reply:**

We sincerely thank the reviewer for this important and thoughtful comment. We agree that the self-selected, self-funded nature of our cohort limits the generalizability of the findings and may influence both prevalence estimates and risk factor associations.

In the revised Discussion, we have carefully moderated the wording to avoid overgeneralization and removed statements implying national-level screening prioritization. The findings are now explicitly framed as applicable primarily to a self-selected screening population at a single private center (page 17, lines 314-317). In addition, as detailed in our response to the previous comment, we have strengthened the Limitations section to further emphasize the potential impact of selection bias and to caution against extrapolating these findings to the broader Vietnamese population (page 17, lines 325-331).

“Therefore, our findings describe risk patterns among asymptomatic Vietnamese individuals undergoing self-funded screening colonoscopy in a private clinical setting and may contribute to understanding risk distribution within similar screening populations.”

“First, this was a single-center study involving individuals who elected to undergo self-funded screening colonoscopy. This self-selected recruitment framework may introduce selection bias, as participants may differ from the general population in terms of health awareness, socioeconomic status, and underlying risk profile. Consequently, the estimated prevalence and identified risk associations should be interpreted with caution, and the findings may not be fully generalizable to the broader average-risk Vietnamese population.”

**Comment 2.** *"Asymptomatic" framing runs a risk of conflating screening populations- The Discussion repeatedly uses "asymptomatic individuals" as if this is synonymous with average-risk screening, however inclusion criteria and recruitment likely represent a health-seeking, risk-aware subset (and ascertainment of symptoms is not completely standardized). If "asymptomatic" is misinterpreted, the conclusions on when/whom to screen may be incorrectly applied.*

**Reply:**

We sincerely thank the reviewer for highlighting the potential risk of conflating “asymptomatic” individuals with an average-risk screening population. In response to this concern, we have carefully and systematically revised the Discussion and Conclusion sections to avoid any wording that could imply such equivalence. Specifically:

1. We consistently refer to the study population as a “screening-attending” or “self-funded screening” cohort rather than implying a general average-risk population (page 13; lines 229-231; page 14, lines 242-245; and page 14, lines 248-251).
2. We have removed or softened statements that could be interpreted as direct recommendations regarding national screening age or target populations (page 18, lines 349-355).
3. Interpretations related to screening age are now framed explicitly as hypothesis-generating and require confirmation from large-scale, population-based studies before informing future screening strategies (page 15, lines 276-281 and page 18, lines 349-355).
4. The Conclusion has been revised to clearly specify that the findings apply to asymptomatic Vietnamese adults undergoing self-funded screening colonoscopy, thereby limiting the scope of inference (page 18, lines 356-359).

“Our results in asymptomatic individuals ≥ 18 years who underwent self-funded screening colonoscopy in a private clinical setting indicated that the prevalence of CRN and advanced CRN were 26.2% and 9%, respectively.”

“Overall, the prevalence of CRN and advanced CRN in our study was higher than that reported in previous studies conducted in the Asia Pacific region; however, differences in study design, population characteristics, and screening context may partly explain these variations.”

“The high proportion of advanced CRN in this study may be attributed to the inclusion of symptomatic patients undergoing colonoscopy, rather than individuals undergoing voluntary screening colonoscopy without symptoms.”

“Therefore, these data suggest a potential burden of CRC in younger adults within screening-attending populations. Taken together, these findings raise the hypothesis that the optimal age threshold for CRC screening in Vietnam may differ from that currently recommended in the Asia-Pacific region [3]. However, this question requires confirmation through large-scale, population-based studies before informing national policy.”

“In this screening-attending population, our data highlight a non-negligible burden of CRN among individuals aged ≥ 40 years. Taken together with prior Vietnamese reports suggesting a considerable proportion of early-onset CRC, these findings raise the hypothesis that the appropriate age for initiating CRC screening in Vietnam warrants further investigation. Because Vietnam currently lacks an established national CRC screening program, confirmation from large-scale, population-based studies is essential before these observations could inform the development of future screening strategies.”

“In conclusion, CRN was prevalent among asymptomatic Vietnamese adults undergoing self-funded screening colonoscopy. Age ≥ 40 years, overweight status, and family history of CRC were independently associated with CRN in this screening-attending population.”

**Comment 3.** *Causal or directive language extends beyond the limitations imposed on cross-sectional data - Assertions that certain criteria detected "ought to be considered to prioritise subjects" go beyond association and approach prescriptive screening recommendations. PLOS ONE expects conclusions to equal design limits; cross sectional associations do not make case for causality, or optimal triage rules.*

**Reply:**

We sincerely thank the reviewer for this constructive and important comment. In response, we have carefully revised the Discussion and Conclusion sections to ensure full alignment with the study design. Specifically, directive or prescriptive expressions such as “ought to be considered to prioritise subjects” and references to a “risk-stratified approach” have been removed. All statements have been reframed to emphasize observed associations rather than causal inference or screening directives. Furthermore, the entire Discussion and Conclusion have been thoroughly reviewed and edited to eliminate any language that could be interpreted as extending beyond the limitations inherent to cross-sectional data. In addition, we have further strengthened the Limitations section to explicitly acknowledge the inherent constraints of the study design (page 18, lines 347-348).

“Fifth, owing to the cross-sectional design of this study, temporal relationships cannot be established, and therefore causal inferences cannot be made.”

**Comment 4.** *Limitations are enumerated, important ones underemphasized- The paper has listed some limitations (single center; exclusions; missing risk factors), but failed to anticipate the most serious threats: self-paid volunteer sampling, possible inaccurate classification of symptoms, and instability of models in the case of advanced CRN (wide CIs). Framing Incomplete limitation may cause conclusions to seem stronger than they should be, leading to high rejection or revision risk.*

**Reply:**

We sincerely thank the reviewer for this important comment and fully agree with the concern raised. In the revised manuscript, we have substantially strengthened the Limitations section to explicitly address the key methodological threats highlighted. Specifically, we now clearly emphasize (1) the potential selection bias related to self-paid volunteer sampling and its impact on generalizability (page 17, lines 325-331), (2) the possibility of symptom misclassification despite structured clinical assessment (pages 17-18, lines 333-336), and (3) the instability of multivariable estimates for advanced CRN due to the limited number of cases and wide confidence intervals (page 18, lines 344-346). We believe these revisions ensure a more balanced interpretation of the findings and reduce the risk of overstatement.

“First, this was a single-center study involving individuals who elected to undergo self-funded screening colonoscopy. This self-selected recruitment framework may introduce selection bias, as participants may differ from the general population in terms of health awareness, socioeconomic status, and underlying risk profile. Consequently, the estimated prevalence and observed associations should be interpreted with caution, and the findings may not be fully generalizable to the broader average-risk Vietnamese population.”

“Second, although asymptomatic status was determined through structured clinical assessment by experienced gastroenterologists, some degree of symptom misclassification cannot be entirely excluded, which may have influenced prevalence estimates.”

“Fourth, the number of advanced CRN cases was relatively limited, leading to wider confidence intervals and potential instability of multivariable estimates. Therefore, associations involving advanced CRN should be interpreted cautiously.”

Once again, we would like to thank you for your time, and we greatly appreciate your insights that helped us refine the manuscript.

With the warmest regards,

On behalf of the authors

Duc Quach, MD; PhD

Department of Internal Medicine and Gastro-Hepato Integrated Research Team (GHIRT-002.TCM2025), School of Medicine, University of Medicine and Pharmacy at Ho Chi Minh City, Vietnam

217 Hong Bang Street, Cho Lon Ward, Ho Chi Minh City, Vietnam

Tel: +84-91-808-025

Email: [drquachtd@ump.edu.vn](mailto:drquachtd@ump.edu.vn)
