# Supplementary material for: Prevalence and risk factors for colorectal neoplasia in a self-selected Vietnamese screening cohort undergoing self-funded colonoscopy
Source: PLoS One. 2026 Jul 13;21(7):e0352998. doi: 10.1371/journal.pone.0352998 (PMC13362143; doi:10.1371/journal.pone.0352998)
Supplement: Attachment — Submitted filename: point-to-ponit reply- R2.docx [file pone.0352998.s008.docx]

April 21, 2026

Dear Editor,

We would like to thank you very much for your kindness in allowing us to revise our manuscript. We also thank the reviewers for their constructive comments. The manuscript has been revised accordingly, and the revised sections in the resubmitted manuscript have been highlighted in blue. We would like to respond to your comments as follows.

**Response to Reviewer 1**

**Comment 1. Generalizability and the "Screening-Attending" Population**

*Remaining Concern: While the term "screening-attending" is more accurate, the Discussion and Conclusion could still be misconstrued by a casual reader. The findings are highly valuable for describing this specific cohort and for generating hypotheses, but they should not be interpreted as directly applicable to the "average-risk Vietnamese population" at large. The authors have done a good job of inserting cautionary language, but the final conclusion could be sharpened to reinforce this point. The statement "CRN was prevalent among asymptomatic Vietnamese adults undergoing self-funded screening colonoscopy" is precise and should be the central takeaway.*

**Reply:**

We thank the reviewer for this thoughtful comment. We agree that, despite our previous revisions, the findings could still be interpreted as being applicable to the general Vietnamese population. To address this, we have revised the Conclusion to more clearly state that our results reflect a self-selected, screening-attending population, and should not be directly generalized to the average-risk population (page 19, lines 360-361).

“In conclusion, CRN was prevalent among asymptomatic Vietnamese adults undergoing self-funded screening colonoscopy. Age ≥ 40 years, overweight status, and family history of CRC were independently associated with CRN in this population. These findings should be interpreted with caution and may not be directly generalizable to the average-risk Vietnamese population.”

**Comment 2. Inference on Screening Age Threshold:**

*Remaining Concern: The discussion of the age threshold (lines 276-281) is well-balanced. However, it is important to reiterate that the finding of a significant risk at age ≥ 40 in this cohort does not, by itself, provide evidence for the efficacy or cost-effectiveness of starting screening at that age in the general population. The authors acknowledge the need for population-based studies, which is sufficient. No further action is needed, as the current phrasing is appropriate.*

**Reply:**

We agree with the reviewer’s point. The observed association at age ≥40 years in this cohort should not be interpreted as evidence for screening effectiveness or policy change. As already revised in the previous version, we have framed this finding as hypothesis-generating and emphasized the need for population-based studies. We have kept the current wording, as it already reflects this limitation.

**Comment 2. Handling of the NICE Type I Discordance:**

*Remaining Concern: While the explanation is plausible, the manuscript could briefly mention that this discordance, while not affecting the final pathology-based diagnosis, highlights a limitation of real-time optical diagnosis in this setting. The current text in the Discussion (lines 259-264) adequately frames this as a known challenge.*

**Reply:**

We thank the reviewer for this constructive comment. We have added a sentence in the Discussion to clarify that this discordance reflects a limitation of real-time optical diagnosis, particularly in small lesions (page 15, lines 264-265).

“There may be discrepancies between endoscopic appearance and pathological diagnosis, particularly in small lesions where optical differentiation between diminutive hyperplastic, serrated, and low-grade neoplastic lesions can be challenging [27]. This also highlights a limitation of real-time optical diagnosis in routine clinical practice.”

**Minor Comments**

**Reply:**

We thank the reviewer for the positive feedback on the revised manuscript. We are glad that the previous concerns regarding the abstract, introduction, methods, results, and discussion have been adequately addressed. No further changes were made in response to these points.

**Response to Reviewer 2**

**Comment.** *Most areas of concern from the title, abstract, introduction, method, result and discussion have been addressed, and others were presented as limitations.*

**Reply:**

We thank the reviewer for the careful review and for noting that the major concerns have been addressed. We appreciate the positive assessment of the revised manuscript.

Once again, we would like to thank you for your time, and we greatly appreciate your insights that helped us refine the manuscript.

With the warmest regards,

On behalf of the authors

Duc Quach, MD; PhD

Department of Internal Medicine and Gastro-Hepato Integrated Research Team (GHIRT-002.TCM2025), School of Medicine, University of Medicine and Pharmacy at Ho Chi Minh City, Vietnam

217 Hong Bang Street, Cho Lon Ward, Ho Chi Minh City, Vietnam

Tel: +84-91-808-025

Email: [drquachtd@ump.edu.vn](mailto:drquachtd@ump.edu.vn)
