# Supplementary material for: Prevalence and risk factors for colorectal neoplasia in a self-selected Vietnamese screening cohort undergoing self-funded colonoscopy
Source: PLoS One. 2026 Jul 13;21(7):e0352998. doi: 10.1371/journal.pone.0352998 (PMC13362143; doi:10.1371/journal.pone.0352998)
Supplement: Attachment — Submitted filename: Response - Plos One - R4.docx [file pone.0352998.s010.docx]

May 24, 2026

Dear Editor,

We would like to thank you very much for your kindness in allowing us to revise our manuscript. We also thank the reviewers for their constructive comments. The manuscript has been revised accordingly, and the revised sections in the resubmitted manuscript have been highlighted in blue. We would like to respond to your comments as follows.

**Response to Reviewer 1**

**Comment 1. Severe selection bias undermines generalizability (unchanged from prior review).**

*Critical issue: The prevalence estimates (26.2% overall CRN, 9.0% advanced CRN) cannot be extrapolated to the general population. The authors correctly note that the study was "not designed to provide population-level prevalence estimates," yet the abstract, results, and discussion repeatedly imply broader relevance. For a high-impact journal, a single-center, self-selected cohort cannot support meaningful prevalence estimates – even as a “hypothesis-generating” study, the bias is too large to justify any conclusion about the Vietnamese population.
Required action:*

*Revise the title and abstract to explicitly state that findings are from a self-selected screening-attending cohort in a private clinical setting, not representative of asymptomatic Vietnamese adults.*

*Remove or substantially tone down statements implying generalizability (e.g., "CRN was prevalent in asymptomatic Vietnamese adults" should become "CRN was prevalent in this self-selected screening cohort").*

*Add a dedicated paragraph in the Discussion quantifying the expected direction and magnitude of selection bias.*

**Reply:**

We thank the Reviewer for the valuable comment regarding selection bias and overgeneralizability. Accordingly, we have: (1) revised the Title, Abstract, Introduction, and Discussion to clearly state that this was a self-selected, screening-attending cohort recruited in a private clinical setting; (2) replaced generalized statements “CRN was prevalent in asymptomatic Vietnamese adults” with more accurate wording “CRN was prevalent in this self-selected screening cohort”; and (3) added a dedicated paragraph in the Discussion quantifying the expected direction and magnitude of selection bias.

**Title**

“Prevalence and risk factors for colorectal neoplasia in a self-selected Vietnamese screening cohort undergoing self-funded colonoscopy”

**Abstract** (page 2, line 34; page 3, lines 42-43)

“In this screening-attending cohort, the prevalence of overall CRN and advanced CRN were 26.2% and 9.0%, respectively.”

“CRN was prevalent in this self-selected screening-attending cohort in a private clinical setting”.

**Introduction** (page 5, lines 73-74)

“Consequently, identifying the prevalence and associated risk factors for CRN may contribute preliminary evidence relevant to future CRC screening strategies in Vietnam.”

**Discussion** (page 19, lines 359-367; page 20, lines 396-397; page 21, lines 403-404)

“This study had several limitations. First, it was a single-center study involving individuals who voluntarily underwent self-funded screening colonoscopy in a private clinical setting, which may have introduced selection bias. Participants were likely more health-conscious and had better healthcare access than the general Vietnamese population, potentially resulting in higher prevalence estimates than those in the broader average-risk population. Participants were likely more health-conscious and had better healthcare access than the general Vietnamese population, potentially resulting in higher prevalence estimates than those in the broader average-risk population.”

“In this screening-attending population, our data suggests a notable burden of CRN within this screening-attending cohort.”

“In conclusion, CRN was prevalent in this self-selected screening-attending cohort undergoing self-funded colonoscopy.”

**Comment 2. Post-hoc age dichotomization is a serious methodological flaw**

*Re-analyze the data with age as a continuous variable (e.g., per 10-year increment) and report adjusted odds ratios.*

*If dichotomization is retained, provide a pre-specified analysis plan or external validation of the 40-year cutoff from an independent dataset.*

*Alternatively, present both continuous and dichotomized models in supplemental materials and clearly state that the dichotomized analysis is exploratory.*

**Reply:**

We thank the reviewer for this important methodological comment. We agree that modeling age as a continuous variable is statistically more appropriate than relying solely on dichotomized thresholds. Accordingly, we re-analyzed the data using age as a continuous variable expressed per 10-year increment in the primary multivariable logistic regression models. These revised analyses have been incorporated into the Methods, Results, Table 3, and Discusssion of the revised manuscript.

We retained the age threshold analysis of ≥ 40 years as an exploratory secondary analysis because previous Vietnamese and Asia-Pacific studies have suggested a relatively younger age distribution of CRN in this region. The manuscript has been revised to clarify that this cutoff was exploratory and was not intended to define an optimal screening age.

**Methods** (page 9, lines 184-188)

“In the primary multivariable logistic regression analysis, age was modeled as a continuous variable expressed per 10-year increment. An exploratory secondary analysis using an age cutoff of 40 years was also performed because previous Vietnamese and Asia-Pacific studies have suggested a relatively younger age distribution of CRN in this region [4, 10].”

**Results** (page 13, lines 225-229; page 13, lines 233-237)

“In the primary multivariable analysis, increasing age per 10-year increment (odds ratio [OR]: 1.76; 95% CI: 1.47-2.11; p < 0.001), BMI ≥ 23 kg/m² (OR: 1.70; 95% CI: 1.16-2.50; p = 0.006), alcohol consumption (OR: 1.83; 95% CI: 1.10-3.04; p = 0.020), and family history of CRC (OR: 2.43; 95% CI: 1.36-4.37; p = 0.003) were independently associated with overall CRN (Table 3).”

**Table 3: Multivariable logistic regression analysis of factors associated with colorectal neoplasia**

| Risk factors | Overall colorectal neoplasia | | |
| --- | --- | --- | --- |
|  | OR | 95% CI | p value |
| Male | 1.16 | 0.71-1.91 | 0.551 |
| Age (per 10-year increase) | 1.76 | 1.47- 2.11 | < 0.001 |
| BMI ≥ 23 kg/m^2^ | 1.70 | 1.16-2.50 | 0.006 |
| Smoking | 1.24 | 0.72-2.11 | 0.440 |
| Alcohol consumption | 1.83 | 1.10-3.04 | 0.020 |
| Family history of CRC | 2.43 | 1.36-4.37 | 0.003 |

*OR: odds ratio, CI: confidence interval, BMI: Body mass index, CRC: Colorectal cancer*

“In the exploratory secondary analysis using an age threshold of ≥40 years, participants aged ≥40 years showed higher odds of overall CRN (OR: 2.79; 95% CI: 1.40-5.56; p = 0.004). BMI ≥23 kg/m2 (OR: 1.92; 95% CI: 1.32-2.79; p = 0.001) and family history of CRC (OR: 2.29; 95% CI: 1.29-4.05; p = 0.005) were also associated with overall CRN.”

**Supplementary Table 2: Exploratory multivariable logistic regression analysis of factors associated with colorectal neoplasia and advanced colorectal neoplasia**

| Risk factors | Overall colorectal neoplasia | | | Advanced colorectal neoplasia | | |
| --- | --- | --- | --- | --- | --- | --- |
|  | OR | 95% CI | p value | OR | 95% CI | p value |
| Male | 1.15 | 0.71-1.86 | 0.575 | 1.44 | 0.70-2.97 | 0.322 |
| Age ≥ 40 | 2.79 | 1.40- 5.56 | 0.004 | 5.20 | 1.22-22.11 | 0.025 |
| BMI ≥ 23 | 1.92 | 1.32-2.79 | 0.001 | 1.62 | 0.91-2.88 | 0.102 |
| Smoking | 1.32 | 0.78-2.23 | 0.307 | 0.75 | 0.35-1.61 | 0.461 |
| Alcohol consumption | 1.47 | 0.91-2.39 | 0.117 | 1.58 | 0.77-3.23 | 0.212 |
| Family history of CRC | 2.29 | 1.29-4.05 | 0.005 | 2.01 | 0.92-4.40 | 0.082 |

*OR: odds ratio, CI: confidence interval, BMI: Body mass index, CRC: Colorectal cancer*

**Discussion** (page 14, lines 292-313)

“In the primary multivariable analysis, increasing age modeled as a continuous variable per 10-year increment remained independently associated with both overall CRN and advanced CRN. BMI ≥ 23 kg/m², alcohol consumption, and family history of CRC were also independent risk factors for overall CRN. These findings are consistent with those from a large prospective multinational study in asymptomatic Asian populations, which identified advanced age, male sex, and family history of CRC as key risk factors for CRN [25]. Previous studies have also indicated that increasing age is an important risk factor for the development of CRN and CRC [22, 24]. In Vietnam, several studies have suggested a relatively younger age distribution of CRN and CRC compared with that reported in some other countries. A retrospective study conducted on 2,525 outpatients with advanced CRN revealed that the rate of advanced CRN in the group under 50 years of age was 23.4%, whereas that in the group < 40 years of age was 8.2% [13]. Another prospective study reported a significantly high proportion of early-onset CRC (i.e., < 50 years of age) of 28%, of which 11% of patients were under 40 years of age [4]. Therefore, these data suggest a potential burden of CRC in younger adults within screening-attending populations. Based on these prior observations, we additionally performed an exploratory secondary analysis using an age threshold of ≥ 40 years. In this exploratory model, individuals aged ≥40 years had significantly increased odds of both overall CRN and advanced CRN. These observations highlight the need for further investigation into age-specific risk stratification in this context. However, this observation should be interpreted as hypothesis-generating, and confirmation from large-scale, population-based studies is required before informing national policy.”

**Comment 3. Inadequate control for confounding**

*Calculate E-values for the main findings (age, BMI, family history) to quantify the minimum strength of association that an unmeasured confounder would need to have with both exposure and outcome to explain away the observed effects.*

*Explicitly state that causal inferences cannot be drawn and that the reported associations may be confounded.*

**Reply:**

We thank the reviewer for the valuable comment. We acknowledge that residual confounding from unmeasured variables remains possible because of the observational and self-selected nature of the cohort.

To further evaluate the robustness of the observed associations, we calculated E-values for the main findings in the primary multivariable analysis. The E-values were 1.98 for age per 10-year increment, 1.93 for BMI ≥ 23 kg/m², 2.04 for alcohol consumption, and 2.49 for family history of CRC (Supplementary Table 3). These findings suggest that unmeasured confounding of at least moderate magnitude would be required to fully explain away the observed associations.

We have revised the Discussion to explicitly state that causal inferences cannot be drawn from this cross-sectional observational study and that the reported associations should be interpreted cautiously because residual confounding may persist despite multivariable adjustment (page 20, lines 381-389; page 20, lines 394-395).

“Third, some factors potentially associated with CRN, such as diabetes mellitus, dyslipidemia, physical inactivity, and dietary factors including high red meat intake and low fiber consumption, were not considered in our study. Furthermore, smoking and alcohol consumption were categorized using simplified definitions and were not evaluated quantitatively. These limitations may introduce residual confounding, and the observed associations should therefore be interpreted cautiously. E-value analyses indicated that unmeasured confounding of at least moderate magnitude would be required to fully explain away the observed associations (Supplementary Table 3).”

“Fifth, owing to the cross-sectional design of this study, temporal relationships cannot be established, and therefore causal inferences cannot be made.”

**Supplementary Table 3. E-values for significant factors associated with colorectal neoplasia in the primary multivariable analysis**

| Risk factors | Adjusted Odds Ratio (95% CI)* | E-value for point estimate | E-value for lower bound of 95% CI |
| --- | --- | --- | --- |
| Age (per 10-year increment) | 1.76 (1.47 – 2.11) | 1.98 | 1.72 |
| BMI ≥ 23 kg/m² | 1.70 (1.16 – 2.50) | 1.93 | 1.37 |
| Alcohol consumption | 1.83 (1.10 – 3.04) | 2.04 | 1.28 |
| Family history of CRC | 2.43 (1.36 – 4.37) | 2.49 | 1.61 |

*OR: odds ratio, CI: confidence interval, BMI: Body mass index, CRC: Colorectal cancer*.

E-value was calculated with the following formula for colorectal neoplasia prevalence > 15%:

**Comment 4. Underpowered analysis for advanced CRN**

*Move the advanced CRN analysis to supplemental materials with a clear disclaimer that it is exploratory and underpowered.*

*Remove statements in the abstract and results that suggest any conclusion about advanced CRN beyond "age may be associated, but estimates are imprecise."*

**Reply:**

We thank the reviewer for this helpful comment. We agree that the number of advanced CRN cases was relatively limited, resulting in wider confidence intervals and potentially unstable estimates. Accordingly, the multivariable analysis for advanced CRN has been moved to Supplementary Materials. We also checked the Abstract and revised the Results to clarify that advanced CRN analysis should be considered exploratory (page 13, lines 229-232; page 13, lines 237-240).

“For advanced CRN, increasing age may be associated with the outcome in the exploratory multivariable analysis; however, the estimates were imprecise, with relatively wide confidence intervals because of the limited number of advanced CRN cases (Supplementary Table 1).”

“Similarly, participants aged ≥ 40 years showed a possible increase in the odds of advanced CRN in the exploratory analysis, although the estimates were imprecise because of the limited number of events (OR: 5.20; 95% CI: 1.22-22.11; p = 0.025) (Supplementary Table 2).”

**Supplementary Table 1: Exploratory multivariable logistic regression analysis of factors associated with advanced colorectal neoplasia**

| Risk factors | Advanced colorectal neoplasia | | |
| --- | --- | --- | --- |
|  | OR | 95% CI | p value |
| Male | 1.42 | 0.67-2.98 | 0.360 |
| Age (per 10-year increase) | 1.91 | 1.46-2.51 | < 0.001 |
| BMI ≥ 23 kg/m^2^ | 1.43 | 0.79-2.56 | 0.234 |
| Smoking | 0.71 | 0.33-1.55 | 0.394 |
| Alcohol consumption | 2.00 | 0.94-4.26 | 0.071 |
| Family history of CRC | 2.06 | 0.93-4.58 | 0.077 |

*OR: odds ratio, CI: confidence interval, BMI: Body mass index, CRC: Colorectal cancer*

**Comment 5. Unvalidated definition of “asymptomatic”**

*Provide the actual symptom questionnaire or structured interview form as a supplemental file.*

*Report the proportion of participants who endorsed any symptom (even if considered minor) and compare their CRN prevalence to truly asymptomatic participants as a sensitivity analysis.*

*Acknowledge that the lack of a validated instrument is a major limitation.*

**Reply:**

We thank the reviewer for this thoughtful comment. Asymptomatic status in our study was determined through routine clinical interviews rather than a formally validated diagnostic instrument. No formal standardized questionnaire was used during routine clinical assessment; therefore, no structured symptom questionnaire is available as supplementary material. During the pre-procedural evaluation, experienced gastroenterologists utilized a consistent clinical framework to assess lower gastrointestinal symptoms. Participants reporting gastrointestinal symptoms, including mild or intermittent complaints, were excluded as part of the study eligibility criteria. The final dataset did not intentionally include mildly symptomatic individuals; therefore, a comparative sensitivity analysis could not be performed. We have explicitly acknowledged the lack of a validated instrument and the potential for symptom misclassification and recall bias in the revised manuscript (pages 19-20, lines 376-381).

“Second, although asymptomatic status was determined through structured clinical assessment by experienced gastroenterologists—as is typical in routine screening practice—it was not based on a formally validated instrument. Therefore, mild or intermittent gastrointestinal symptoms may have been underrecognized, potentially resulting in some degree of symptom misclassification and recall bias, which should be considered when interpreting the observed prevalence estimates.”

**Comment 6. NICE type I discordance highlights quality issue**

*Provide a stratified analysis of NICE type I discordance by lesion size (≤5 mm vs. >5 mm) and location.*

*Report the inter-endoscopist variation in NICE classification accuracy.*

*Discuss whether any lesions classified as NICE type I were left in situ (i.e., not resected) – if so, the true prevalence of CRN may be underestimated.*

**Reply:**

We thank the reviewer for this important comment. In response, we performed an additional analysis of NICE type I discordance by lesion size and location. NICE type I discordance was substantially more common in diminutive lesions: 42/168 lesions ≤ 5 mm (25.0%) versus 7/144 lesions > 5 mm (4.9%; p < 0.001). By location, discordance rates were 22/137 (16.1%) in the proximal colon, 22/151 (14.6%) in the distal colon, and 5/24 (20.8%) in the rectum (p = 0.727).

In our study, all lesions were resected and histologically examined; therefore, no NICE type I lesions were left unresected. In addition, no significant inter-endoscopist variation in NICE classification accuracy was observed among the participating endoscopists. These findings have been added to the Discussion section of the revised manuscript (pages 15-16, lines 274-285).

“There may be discrepancies between endoscopic appearance and pathological diagnosis, particularly in small lesions where optical differentiation between diminutive hyperplastic, serrated, and low-grade neoplastic lesions can be challenging [27]. In our study, NICE type I discordance was largely confined to lesions ≤ 5 mm and was not significantly associated with lesion location. These findings highlight the limitations of real-time optical diagnosis in routine clinical practice, particularly for diminutive lesions. However, all colonoscopies were performed by experienced endoscopists with established quality indicators (adenoma detection rate >30%), and no significant inter-endoscopist variation in NICE classification accuracy was observed. Importantly, all lesions were resected and histologically examined, which may mitigate the impact of optical misclassification on study outcomes.”

**Comment 7. Single-Center Design with 5 Endoscopists – Lack of External Validity**

*Add a clear statement that external validation in multi-center, population-based samples is required before any screening policy recommendations can be derived from these data.*

**Reply:**

We thank the reviewer for this valuable comment and agree that the single-center design may limit the external generalizability of our findings. Accordingly, we have revised the Discussion to explicitly state that external validation in large-scale, multicenter, population-based studies across diverse healthcare settings in Vietnam is required before any screening policy recommendations can be derived from these data (page 19, lines 370-375).

“Therefore, the findings should be interpreted cautiously and validated in large-scale, multicenter, population-based studies before being generalized to national CRC screening strategies or used to inform screening policy recommendations.”

Once again, we would like to thank you for your time, and we greatly appreciate your insights that helped us refine the manuscript.

With the warmest regards,

On behalf of the authors

Duc Quach, MD; PhD

Department of Internal Medicine and Gastro-Hepato Integrated Research Team (GHIRT-002.TCM2025), School of Medicine, University of Medicine and Pharmacy at Ho Chi Minh City, Vietnam

217 Hong Bang Street, Cho Lon Ward, Ho Chi Minh City, Vietnam

Tel: +84-91-808-025

Email: [drquachtd@ump.edu.vn](mailto:drquachtd@ump.edu.vn)
