# Supplementary material for: Prevalence and risk factors for colorectal neoplasia in a self-selected Vietnamese screening cohort undergoing self-funded colonoscopy
Source: PLoS One. 2026 Jul 13;21(7):e0352998. doi: 10.1371/journal.pone.0352998 (PMC13362143; doi:10.1371/journal.pone.0352998)
Supplement: Peer review history

# Peer review: Decision Letter 0

**DOI:** 10.1371/journal.pone.0352998.r001

**Authors:** Chih-Wei Tseng

19 Feb 2026

PONE-D-25-66438

Prevalence and risk factors for colorectal neoplasia in asymptomatic Vietnamese undergoing screening colonoscopy

PLOS One

Dear Dr. Quach,

Thank you for submitting your manuscript to PLOS ONE. After careful consideration, we feel that it has merit but does not fully meet PLOS ONE’s publication criteria as it currently stands. Therefore, we invite you to submit a revised version of the manuscript that addresses the points raised during the review process.

Please submit your revised manuscript by Apr 05 2026 11:59PM. If you will need more time than this to complete your revisions, please reply to this message or contact the journal office at plosone@plos.org. When you're ready to submit your revision, log on to https://www.editorialmanager.com/pone/ and select the 'Submissions Needing Revision' folder to locate your manuscript file.

Please include the following items when submitting your revised manuscript:

- A letter that responds to each point raised by the academic editor and reviewer(s). You should upload this letter as a separate file labeled 'Response to Reviewers'.
- A marked-up copy of your manuscript that highlights changes made to the original version. You should upload this as a separate file labeled 'Revised Manuscript with Track Changes'.
- An unmarked version of your revised paper without tracked changes. You should upload this as a separate file labeled 'Manuscript'.

If you would like to make changes to your financial disclosure, please include your updated statement in your cover letter. Guidelines for resubmitting your figure files are available below the reviewer comments at the end of this letter.

If applicable, we recommend that you deposit your laboratory protocols in protocols.io to enhance the reproducibility of your results. Protocols.io assigns your protocol its own identifier (DOI) so that it can be cited independently in the future. For instructions see: https://journals.plos.org/plosone/s/submission-guidelines#loc-laboratory-protocols. Additionally, PLOS ONE offers an option for publishing peer-reviewed Lab Protocol articles, which describe protocols hosted on protocols.io. Read more information on sharing protocols at https://plos.org/protocols?utm_medium=editorial-email&utm_source=authorletters&utm_campaign=protocols.

We look forward to receiving your revised manuscript.

Kind regards,

Chih-Wei Tseng

Academic Editor

PLOS One

Journal Requirements:

When submitting your revision, we need you to address these additional requirements.

1. Please ensure that your manuscript meets PLOS ONE's style requirements, including those for file naming. The PLOS ONE style templates can be found at

https://journals.plos.org/plosone/s/file?id=wjVg/PLOSOne_formatting_sample_main_body.pdf and

https://journals.plos.org/plosone/s/file?id=ba62/PLOSOne_formatting_sample_title_authors_affiliations.pdf

2. Please include a complete copy of PLOS’ questionnaire on inclusivity in global research in your revised manuscript. Our policy for research in this area aims to improve transparency in the reporting of research performed outside of researchers’ own country or community. The policy applies to researchers who have travelled to a different country to conduct research, research with Indigenous populations or their lands, and research on cultural artefacts. The questionnaire can also be requested at the journal’s discretion for any other submissions, even if these conditions are not met.  Please find more information on the policy and a link to download a blank copy of the questionnaire here: https://journals.plos.org/plosone/s/best-practices-in-research-reporting. Please upload a completed version of your questionnaire as Supporting Information when you resubmit your manuscript

3. Please upload a copy of Supporting Information S1 Fig, S2 Fig, S1 Table, S2 Table, S3 Table, S1 File. STROBE Checklist which you refer to in your text on page 24.

4. If the reviewer comments include a recommendation to cite specific previously published works, please review and evaluate these publications to determine whether they are relevant and should be cited. There is no requirement to cite these works unless the editor has indicated otherwise.

[Note: HTML markup is below. Please do not edit.]

Reviewers' comments:

Reviewer's Responses to Questions

**Comments to the Author**

1. Is the manuscript technically sound, and do the data support the conclusions?

The manuscript must describe a technically sound piece of scientific research with data that supports the conclusions. Experiments must have been conducted rigorously, with appropriate controls, replication, and sample sizes. The conclusions must be drawn appropriately based on the data presented.

Reviewer #1: Partly

Reviewer #2: Partly

**********

2. Has the statistical analysis been performed appropriately and rigorously?

Reviewer #1: Yes

Reviewer #2: I Don't Know

**********

3. Have the authors made all data underlying the findings in their manuscript fully available?

The PLOS Data policy requires authors to make all data underlying the findings described in their manuscript fully available without restriction, with rare exception (please refer to the Data Availability Statement in the manuscript PDF file). The data should be provided as part of the manuscript or its supporting information, or deposited to a public repository. For example, in addition to summary statistics, the data points behind means, medians and variance measures should be available. If there are restrictions on publicly sharing data—e.g. participant privacy or use of data from a third party—those must be specified.

Reviewer #1: Yes

Reviewer #2: Yes

**********

4. Is the manuscript presented in an intelligible fashion and written in standard English?

PLOS ONE does not copyedit accepted manuscripts, so the language in submitted articles must be clear, correct, and unambiguous. Any typographical or grammatical errors should be corrected at revision, so please note any specific errors here.

Reviewer #1: Yes

Reviewer #2: Yes

**********

5. Review Comments to the Author

Please use the space provided to explain your answers to the questions above. You may also include additional comments for the author, including concerns about dual publication, research ethics, or publication ethics. (Please upload your review as an attachment if it exceeds 20,000 characters)

Reviewer #1: Section-by-Section Appraisal & Suggestions:

Title, Abstract, and Keywords:

Excellent. Clear, concise, and accurately reflect the study.

Introduction:

Well-structured. Establishes the global/regional burden of CRC, the problem of early-onset cancer, and the specific lack of data/policy in Vietnam. Clearly states the study's aim.

Suggestion: Consider adding a sentence explicitly mentioning the lack of data in asymptomatic Vietnamese individuals to further justify the study's focus.

Materials and Methods:

Generally excellent. Ethics, participant recruitment, definitions, and procedures are well-described.

Minor Issues:

Line 105-110 (Definitions): The definition for "Non-alcohol consumption" is slightly awkward ("never drinking or drinking alcohol once per month or less"). Consider rephrasing to "Alcohol consumption was defined as drinking more than once per month."

Line 127-128: "...analysed. The participating endoscopists directly evaluated..." Consider combining: "...recorded and analyzed by the participating endoscopists using standardized data collection sheets."

Line 156: "SPSS (version 23; SPSS Inc, Chicago, IL)" - It's more standard to use "IBM SPSS Statistics for Windows, Version 23.0."

Results:

Clear presentation of participant flow, demographics, and prevalence data.

Critical Error in Table 1: There is a significant formatting/presentation error in the BMI rows. The numbers under "No colorectal neoplasia" and "Colorectal neoplasia" columns for BMI ≥23 and <23 do not align correctly with the headers and total N. The percentages (55.4%, 44.6%, 70.3%, 29.7%) seem to be swapped between the "No CRN" and "CRN" groups or are miscalculated. This must be corrected. The p-value (0.021) suggests a difference, but the displayed numbers are confusing.

Table 1 Footnote: The footnote says p-values for age groups represent comparisons with the reference group (< 40 years). This is typically indicated in the table with superscripts (e.g., a, b, c). Add these superscripts to the p-value column for clarity.

Lines 203-205: The rationale for using age ≥40 as a binary cut-off is very good and should be kept. It strengthens the analysis.

Discussion:

Overall, strong. Effectively compares findings to regional studies, explains potential reasons for higher prevalence, and discusses each risk factor in depth.

Lines 236-240: The paragraph comparing findings to a study on IBS patients is slightly confusing. It states the high proportion of advanced CRN "may be attributed to the inclusion of symptomatic patients," which contrasts with your study's focus on asymptomatic individuals. This comparison may not be the most relevant. Consider focusing more on comparisons with other asymptomatic screening studies.

Lines 272-279: The handling of the conflicting Vietnamese case-control study is excellent – pointing out key methodological differences (asymptomatic vs. unspecified, control group without colonoscopy).

Limitations Section (Lines 294-300): Acknowledges key limitations appropriately. Consider adding that the single-center design, while a limitation, also ensured standardized, high-quality colonoscopy procedures, which is a strength for internal validity.

Conclusion:

Accurate and concise. Appropriately summarizes key findings.

References & Supporting Information:

References appear comprehensive and relevant.

Check Formatting: Ensure all references conform strictly to PLOS ONE's citation style (e.g., journal abbreviations, author lists, etc.).

Supporting information (checklist, figures, tables) is appropriate.

Reviewer #2: Prevalence and risk factors for colorectal neoplasia in asymptomatic Vietnamese undergoing screening colonoscopy .

Major issues with the various subsections

Title, Abstract, Keywords

1)Title

•Scope mismatch / definitional ambiguity ("colorectal neoplasia): The title refers to the lesions that involve the spectrum of CRC on a broad scale, and the manuscript has defined CRN as including adenomas/SSL/CRC and subsequently has reported the advanced CRN separately. There needs to be more specific information (e.g., adenoma/serrated lesions and cancer) or an unequivocated definition in the title/abstract. This is important as it can mislead readers and indexers (to think that the results are cancer or precancer) thereby affecting interpretability and comparability.

•"Asymptomatic" not operationalized in title: Title claims asymptomatic screening population, but "asymptomatic" is defined by absence of lower GI + alarm symptoms and includes self-paid voluntary checkups (selection)."This also implies that this is an average risk screening population; most especially if it's a self-selected cohort, external validity is limited.

•The short title repeats the long title with little shortening. short titles should enhance discoverability and clarity on the layout of journal pages/metadata.

2) Abstract

•Study design & sampling frame not sufficiently explicit: Abstract says "cross-sectional" and "voluntary screening colonoscopy," but does not flag this is a single-center, self-paid, self-selected cohort. This information matters because selection bias can lead to overestimating prevalence and cause distortions in the estimation of risk factors; and readers need this upfront.

•The choices of predictors in the risk factor model are not sufficiently transparent: The abstract refers to age (approximately 40), BMI (approximately 23), and family history as the independent predictors, but does not state the covariates that were considered, as well as the approaches to control the potential confounding factors (e.g., sex, smoking, alcohol consumption).

•Definitions of outcomes not mentioned: CRN and advanced CRN prevalence are provided, but there is no abstract summary of their definition (adenoma/SSL/CRC; advanced criteria). Outcome definition propels prevalence magnitude and cross study comparisons.

3) Keywords

•Keywords are too generic and do not include important indexing terms: Current list does not include "screening colonoscopy," "adenoma," "advanced neoplasia," "serrated lesions," "asymptomatic," and "cross-sectional." Discoverability and appropriate indexing in the databases will be minimized, reducing reach and the chances of citation.

Introduction

•Rationale and knowledge gap are not well specified- Gap is stated ("limited data in asymptomatic") but not sharpened into a testable, novel contribution: The intro repeats that Vietnam lacks screening infrastructure and that asymptomatic data are limited, but does not specify what is uniquely unknown (e.g., age-stratified prevalence including <40, advanced CRN proportion, or risk-factor performance in a self-paid cohort). It is important for the question to be well defined so the reader can assess whether or not the methods and results actually address the question.

•Important construct ("asymptomatic") is not introduced with sufficient precision- The intro presents CRC as "largely asymptomatic until advanced" and calls for screening "even in asymptomatic individuals", but fails to preview the manuscript's operational definition of asymptomatic (no lower GI + no alarm symptoms) and the implications of voluntary/self-paid selection. This is important because the significance of "asymptomatic" has a strong influence on generalizability and risk interpretation.

•Internal logic for the age threshold is not consistent. The introduction claims that there is an increase in early-onset colorectal cancer (CRC) and cites age groups (40, 45, 50); however, no explanation is given in the introduction for including individuals aged 18 and older until the Methods section. Without an explicit a priori rationale, inclusion of very young adults can appear to be a post hoc inclusion and may serve to dilute screening-relevant inference.

Methods --

•Study design & setting Selection bias not handled/mitigated- The research is introduced as a prospective cross-sectional research on voluntary, self-paid screening colonoscopy participants in one private clinic. This is a recruitment frame with a high likelihood of selection bias (health-seeking, greater SES, risk-aware people), and which has the potential to overstate prevalence estimates and mislead risk factor associations unless carefully mitigated.

•"Asymptomatic" definition & ascertainment are under specified. “Asymptomatic” is characterized by the lack of distinctive lower intestinal symptoms and alarm symptoms but Methods fails to indicate how symptoms/anemia/weight loss were evaluated (structured questionnaire vs chart review vs labs), by whom, and at what time in relation to colonoscopy.This is significant because misclassification, especially marginally symptomatic or inadequately screened people, can distort prevalence and relationships.

•Eligibility/exclusion choices may cause systematic changes in prevalence estimates- Excluding patients with inadequate bowel prep (Boston score thresholds), incomplete colonoscopy, and withdrawal time <6 minutes appropriate for quality control but Methods don't say if the excluded patients were different from the included patients (no comparison table) and how many were excluded because of what reason (some of this appears later in Results) Exclusions associated with patient factors (age/comorbidity/constipation) can introduce bias in the estimates of prevalence/risk factors.

•Exposure (risk factor) measurement is too gross and incomplete - Smoking is defined as “currently smoking or ever smoked,” and alcohol as “never/≤1× per month” vs others, with no quantity/duration, no pack-years, no standard-drink metric, and no dietary/physical activity/diabetes/dyslipidemia capture (acknowledged only in Discussion, not planned in Methods). This matters because crude exposure measurement increases residual confounding and can wash out true associations or create spurious ones.

•Outcomes are defined, but key procedural/ histology safeguards missing- CRN/advanced CRN definitions are clear, however, Methods do not state if pathologists were blinded to clinical risk factors/endoscopic impression, whether there was dual reading or how disagreements were resolved. Lack of blinding/standardization can cause classification bias especially for serrated lesion/dysplasia calls.

Results

•Prevalence reporting is not contextual in terms of uncertainty and age standard- The Results reflect CRN and an advanced CRN prevalence of 26.2% and 9.0%, respectively, without the reporting of 95% confidence intervals or age-standardised estimates, despite a significantly age-skewed sample (88.4% aged 40 years and above), In the absence of standardisation and certainty, the reported prevalence may be deceptive, and difficult to compare between different populations.

•Endoscopic characterization vs pathology discordance is flagged but not resolved- The manuscript points out a discrepancy between 15.3% of lesions that were NICE type 1 but "all were histologically confirmed as CRN." This suggests a possible problem of misclassification or quality of documentation, which can lead to lack of confidence in endoscopic evaluation and characterisation of the lesion.

•Results occasionally include interpretation that belongs in Discussion- The Results section speculates on reasons for null associations (e.g. "limited number" and "biological differences"). Results should be descriptive; interpretation here can look like narrative steering too and weakens objectivity.

Discussion

•The generalizability is exaggerated with regard to self-selected, self-paid cohort- Discussion interprets prevalence and risk factors to be informative of wider screening prioritization and national level context although its sample is limited to one private clinic and to individuals who had voluntarily paid to undergo colonoscopy. This is very significant as selection bias can materially change the prevalence and predictor strength. overgeneralizing to mislead policy/clinical implications.

•"Asymptomatic" framing runs a risk of conflating screening populations- The Discussion repeatedly uses "asymptomatic individuals" as if this is synonymous with average-risk screening, however inclusion criteria and recruitment likely represent a health-seeking, risk-aware subset (and ascertainment of symptoms is not completely standardized). if "asymptomatic" is misinterpreted, the conclusions on when/whom to screen may be incorrectly applied.

•Causal or directive language extends beyond the limitations imposed on cross-sectional data - Assertions that certain criteria detected "ought to be considered to prioritise subjects" go beyond association and approach prescriptive screening recommendations. PLOS ONE expects conclusions to equal design limits; cross sectional associations do not make case for causality, or optimal triage rules.

•Limitations are enumerated, important ones underemphasized- The paper has listed some limitations (single center; exclusions; missing risk factors), but failed to anticipate the most serious threats: self-paid volunteer sampling, possible inaccurate classification of symptoms, and instability of models in the case of advanced CRN (wide CIs). Framing Incomplete limitation may cause conclusions to seem stronger than they should be, leading to high rejection or revision risk.

**********

6. PLOS authors have the option to publish the peer review history of their article (what does this mean?). If published, this will include your full peer review and any attached files.

If you choose “no”, your identity will remain anonymous but your review may still be made public.

**Do you want your identity to be public for this peer review?**  For information about this choice, including consent withdrawal, please see our Privacy Policy.

Reviewer #1: No

Reviewer #2: **Yes:**  PROF ANYANWU G EMEKA

**********

[NOTE: If reviewer comments were submitted as an attachment file, they will be attached to this email and accessible via the submission site. Please log into your account, locate the manuscript record, and check for the action link "View Attachments". If this link does not appear, there are no attachment files.]

To ensure your figures meet our technical requirements, please review our figure guidelines: https://journals.plos.org/plosone/s/figures

You may also use PLOS’s free figure tool, NAAS, to help you prepare publication quality figures: https://journals.plos.org/plosone/s/figures#loc-tools-for-figure-preparation.

NAAS will assess whether your figures meet our technical requirements by comparing each figure against our figure specifications.

# Author comment: Author response to Decision Letter 1

**DOI:** 10.1371/journal.pone.0352998.r002

9 Mar 2026

March 03, 2026

Dear Editor,

We would like to thank you very much for your kindness in allowing us to revise our manuscript. We also thank the reviewers for their constructive comments. The manuscript has been revised accordingly, and the revised sections in the resubmitted manuscript have been highlighted in blue. We would like to respond to your comments as follows.

Response to Reviewer 1

1. Introduction

Comment. Consider adding a sentence explicitly mentioning the lack of data in asymptomatic Vietnamese individuals to further justify the study's focus.

Reply:

We sincerely thank you for your valuable suggestion. We have revised the Introduction section accordingly to explicitly highlight the lack of data in asymptomatic Vietnamese individuals (page 5, lines 85-89).

“Previous research on CRN in Vietnam has predominantly focused on symptomatic patients, leaving a substantial gap in evidence regarding the age-stratified prevalence—particularly among adults under 40 years—and the risk factors of CRN in asymptomatic Vietnamese individuals.”

2. Materials and Methods

Comment 1. Line 105-110 (Definitions): The definition for "Non-alcohol consumption" is slightly awkward ("never drinking or drinking alcohol once per month or less"). Consider rephrasing to "Alcohol consumption was defined as drinking more than once per month."

Reply:

Thank you very much for your helpful suggestion. We have revised the definition of alcohol consumption in the Methods section accordingly (page 7, lines 118-119).

“Alcohol consumption was defined as drinking more than once per month.”

Comment 2. Line 127-128: "...analysed. The participating endoscopists directly evaluated..." Consider combining: "...recorded and analyzed by the participating endoscopists using standardized data collection sheets."

Reply:

Thank you very much for your constructive comment. In response, we have revised the Methods section accordingly (page 7, lines 137-138).

“The morphology, location, and size of all the detected polyps were recorded and analysed by the participating endoscopists using standardized data collection sheets.”

Comment 3. Line 156: "SPSS (version 23; SPSS Inc, Chicago, IL)" - It's more standard to use "IBM SPSS Statistics for Windows, Version 23.0."

Reply:

We appreciate the reviewer’s suggestion and have updated the software description in the statistical analysis section accordingly (page 9, lines 169-170).

“Statistical analyses were performed with IBM SPSS Statistics for Windows, version 23.0.”

3. Results

Comment 1. Critical Error in Table 1: There is a significant formatting/presentation error in the BMI rows. The numbers under "No colorectal neoplasia" and "Colorectal neoplasia" columns for BMI ≥23 and <23 do not align correctly with the headers and total N. The percentages (55.4%, 44.6%, 70.3%, 29.7%) seem to be swapped between the "No CRN" and "CRN" groups or are miscalculated. This must be corrected. The p-value (0.021) suggests a difference, but the displayed numbers are confusing.

Reply:

Thank you very much for pointing out this error. We sincerely apologize for the typographical errors in the BMI rows of Table 1, which occurred during the final formatting of the manuscript. We have corrected these figures in the revised Table 1 and have thoroughly rechecked the whole table against our raw data. We would like to clarify that these were reporting mistakes only; therefore, the independent risk factors, odds ratios, and p-values of the multivariable analysis are accurate and remain unchanged.

Comment 2. Table 1 Footnote: The footnote says p-values for age groups represent comparisons with the reference group (< 40 years). This is typically indicated in the table with superscripts (e.g., a, b, c). Add these superscripts to the p-value column for clarity.

Reply:

We would like to thank you for your constructive comment. We have added superscripts (e.g., a, b, c) to the p-value column in Table 1 to clearly indicate that these values represent comparisons between each age group and the reference group (< 40 years). The footnote has also been revised to include these indicators.

Table 1. Characteristics of participants with and without colorectal neoplasia

Characteristics Total

(n, %)

N = 714 No colorectal neoplasia (n, %)

N = 527 Colorectal neoplasia

(n, %)

N = 187 p-value

Sex

Male 290 (40.6) 194 (36.8) 96 (51.3) 0.001

Female 424 (59.4) 333 (63.2) 91 (48.7)

Age

< 40 83 (11.6) 72 (13.7) 11 (5.9)

40-49 221 (31.0) 175 (33.2) 46 (24.6) 0.157 a

50-59 238 (33.3) 177 (33.6) 61 (32.6) 0.134 b

60-69 146 (20.4) 88 (16.7) 58 (31.0) 0.004 c

≥ 70 26 (3.6) 15 (2.8) 11 (5.9) 0.026 d

BMI (kg/m2)

≥ 23 405 (56.7) 274 (52.0) 131 (70.1) < 0.001

< 23 309 (43.3) 253 (48.0) 56 (29.9)

Smoking

Yes 105 (14.7) 64 (12.1) 41 (21.9) 0.001

No 609 (85.3) 463 (87.9) 146 (78.1)

Alcohol consumption

Yes 208 (29.1) 132 (25.0) 76 (40.6) < 0.001

No 506 (70.9) 395 (75.0) 111 (59.4)

Family history of CRC

Yes 60 (8.4) 35 (6.6) 25 (13.4) 0.04

No 654 (91.6) 492 (93.4) 162 (86.6)

OR: Odds ratio, CI: Confidence interval, BMI: Body mass index, CRC: Colorectal cancer

a, b, c, d p-values represent comparisons of the 40-49, 50-59, 60-69, and ≥ 70 years age groups, respectively, with the reference group (< 40 years)

Comment 3. Lines 203-205: The rationale for using age ≥40 as a binary cut-off is very good and should be kept. It strengthens the analysis.

Reply:

Thank you very much for this encouraging and supportive comment. We appreciate the reviewer’s assessment that the age ≥ 40 cut-off strengthens the analysis; therefore, we have retained this rationale in the revised manuscript.

4. Discussion

Comment 1. Lines 236-240: The paragraph comparing findings to a study on IBS patients is slightly confusing. It states the high proportion of advanced CRN "may be attributed to the inclusion of symptomatic patients," which contrasts with your study's focus on asymptomatic individuals. This comparison may not be the most relevant. Consider focusing more on comparisons with other asymptomatic screening studies.

Reply:

We sincerely thank the reviewer for this insightful comment. We acknowledge that comparisons with a study of symptomatic IBS patients may be less directly relevant to our asymptomatic screening population. In the revised manuscript, we have therefore prioritized comparisons with asymptomatic screening studies from the Asia-Pacific region (pages 13-14, lines 234-236 and pages 14, lines 237-240).

Given the absence of published data on CRN prevalence among asymptomatic individuals in Vietnam, we have referred to the IBS cohort solely as contextual national background information. As kindly suggested, we have clarified in the revised manuscript that this comparison does not represent a directly comparable screening population (page 14, lines 246-248).

“The prevalence of overall CRN and advanced CRN in asymptomatic subjects from Asia Pacific studies ranged from 19.2-36.0%, and from 1.7-15.4%, respectively [22-24].”

“Another prospective multinational multicenter survey of asymptomatic subjects over 16 years old in 11 Asian cities reported that the prevalence of CRN and advanced CRN were 18.5% and 4.5%, respectively [25].”

“Currently, no published data are available on CRN prevalence among asymptomatic individuals in Vietnam. The only available local data derived from a cohort of patients with irritable bowel syndrome, with rates of 9.1% and 6.9%, respectively [26].”

Comment 2. Limitations Section (Lines 294-300): Acknowledges key limitations appropriately. Consider adding that the single-center design, while a limitation, also ensured standardized, high-quality colonoscopy procedures, which is a strength for internal validity.

Reply:

Thank you very much for your helpful suggestion. We totally agree that while the single-center design limits generalizability, it offers procedural consistency. We have incorporated this point into the revised manuscript to strengthen the internal validity of our study (page 17, lines 331-333).

“Nonetheless, the single-center setting allowed for standardized colonoscopy procedures and centralized histopathological evaluation, which may enhance internal consistency.”

Response to Reviewer 2

1. Title

Comment 1: Scope mismatch / definitional ambiguity ("colorectal neoplasia): The title refers to the lesions that involve the spectrum of CRC on a broad scale, and the manuscript has defined CRN as including adenomas/SSL/CRC and subsequently has reported the advanced CRN separately. There needs to be more specific information (e.g., adenoma/serrated lesions and cancer) or an unequivocated definition in the title/abstract. This is important as it can mislead readers and indexers (to think that the results are cancer or precancer) thereby affecting interpretability and comparability.

Reply:

We sincerely thank the reviewer for this insightful comment regarding the potential for definitional ambiguity of the term colorectal neoplasia (CRN). As you kindly suggested, we have added an unequivocal definition of CRN in the abstract of the revised manuscript to encompass the broad clinical spectrum of lesions, ranging from precancerous precursors to invasive carcinoma (page 2, lines 25-26). We have also ensured that the distinction between overall CRN and advanced CRN is consistently maintained throughout the manuscript to improve clarity and interpretability.

“CRN was defined as the presence of adenoma, sessile serrated lesions, or colorectal cancer.”

Comment 2: Asymptomatic" not operationalized in title: Title claims asymptomatic screening population, but "asymptomatic" is defined by absence of lower GI + alarm symptoms and includes self-paid voluntary checkups (selection)."This also implies that this is an average risk screening population; most especially if it's a self-selected cohort, external validity is limited.

Reply:

Thank you very much for your valuable comment. We agree that a self-selected, self-paid cohort may limit the external validity and generalizability of the findings compared to a population-based average-risk group. To address this, we have updated the title to "Prevalence and risk factors for colorectal neoplasia in asymptomatic Vietnamese adults undergoing self-funded screening colonoscopy" to accurately reflect the voluntary, self-paid status of the participants.

Comment 3: The short title repeats the long title with little shortening. short titles should enhance discoverability and clarity on the layout of journal pages/metadata.

Reply:

We appreciate the reviewer’s suggestion to improve the short title for better clarity and discoverability. We have revised the short title to be more concise while retaining the study's core focus, as you kindly suggested.

"Colorectal neoplasia in asymptomatic Vietnamese adults"

2. Abstract

Comment 1: Study design & sampling frame not sufficiently explicit: Abstract says "cross-sectional" and "voluntary screening colonoscopy," but does not flag this is a single-center, self-paid, self-selected cohort. This information matters because selection bias can lead to overestimating prevalence and cause distortions in the estimation of risk factors; and readers need this upfront.

Reply:

Thank you very much for your kind suggestion. We agree that explicitly identifying the cohort as a single-center, self-selected, and self-paid group in the Abstract is essential for readers to accurately interpret the prevalence and risk factors. Consequently, we have revised the Abstract to include this information (page 2, lines 23-25).

"This was a prospective, cross-sectional, single-center study. Participants were consecutively recruited from asymptomatic individuals who were self-selected to undergo self-funded screening colonoscopy."

Comment 2: The choices of predictors in the risk factor model are not sufficiently transparent: The abstract refers to age (approximately 40), BMI (approximately 23), and family history as the independent predictors, but does not state the covariates that were considered, as well as the approaches to control the potential confounding factors (e.g., sex, smoking, alcohol consumption).

Reply:

We thank the reviewer for this important comment. We have revised the Abstract to clearly specify that age, sex, BMI, family history of colorectal cancer, smoking status, and alcohol consumption were included in the multivariable logistic regression model to adjust for potential confounding (page 2, lines 29-31).

“Multivariable logistic regression was performed to identify independent risk factors for CRN, adjusting for age, sex, BMI, family history of colorectal cancer, smoking status, and alcohol consumption.”

Comment 3: Definitions of outcomes not mentioned: CRN and advanced CRN prevalence are provided, but there is no abstract summary of their definition (adenoma/SSL/CRC; advanced criteria). Outcome definition propels prevalence magnitude and cross study comparisons.

Reply:

Thank you very much for your valuable comment on the need for clear definitions of CRN and advanced CRN. To address this, we have incorporated a summary of the criteria for CRN and advanced CRN into the Abstract of the revised manuscript (page 2, lines 25-28).

“CRN was defined as the presence of adenoma, sessile serrated lesions, or colorectal cancer. Advanced CRN included adenoma ≥ 1 cm, with villous features or high-grade dysplasia; sessile serrated lesion ≥ 1 cm or with dysplasia; traditional serrated adenoma; or colorectal cancer."

3. Keywords

Comment: Keywords are too generic and do not include important indexing terms: Current list does not include "screening colonoscopy," "adenoma," "advanced neoplasia," "serrated lesions," "asymptomatic," and "cross-sectional." Discoverability and appropriate indexing in the databases will be minimized, reducing reach and the chances of citation

Reply:

Thank you very much for this valuable suggestion. In the revised manuscript, we have updated the keyword section to include more specific and relevant indexing terms, including “screening colonoscopy,” “adenoma,” “advanced neoplasia,” “serrated lesions,” “asymptomatic,” and “cross-sectional” (page 3, lines 42-43).

4. Introduction

Comment 1. Rationale and knowledge gap are not well specified- Gap is stated ("limited data in asymptomatic") but not sharpened into a testable, novel contribution: The intro repeats that Vietnam lacks screening infrastructure and that asymptomatic data are limited, but does not specify what is uniquely unknown (e.g., age-stratified prevalence including <40, advanced CRN proportion, or risk-factor performance in a self-paid cohort). It is important for the question to be well defined so the reader can assess whether or not the methods and results actually address the question.

Reply:

We appreciate the reviewer's invaluable comment and have revised the introduction section accordingly (page 5, lines 85-89).

“Previous research on CRN in Vietnam has predominantly focused on symptomatic patients, leaving a substantial gap in evidence regarding the age-stratified prevalence—particularly among adults under 40 years—and the risk factors of CRN in asymptomatic Vietnamese individuals.”

Comment 2. Important construct ("asymptomatic") is not introduced with sufficient precision- The intro presents CRC as "largely asymptomatic until advanced" and calls for screening "even in asymptomatic individuals", but fails to preview the manuscript's operational definition of asymptomatic (no lower GI + no alarm symptoms) and the implications of voluntary/self-paid selection. This is important because the significance of "asymptomatic" has a strong influence on generalizability and risk interpretation.

Reply:

Thank you very much for your insightful comment. In response, we have revised the manuscript to clearly define the term “asymptomatic” and to explicitly state the voluntary, self-paid characteristics of our study population (page 4, lines 62-64; page 6, lines 101-105; and page 17, lines 325-331).

“Therefore, CRC screening for early detection plays a critical role, including among asymptomatic individuals, defined as those without lower gastrointestinal symptoms or alarm features.”

“The eligibility criteria for this study were: age ≥18 years

Attachment Submitted filename: point-to-ponit reply.docx

# Peer review: Decision Letter 1

**DOI:** 10.1371/journal.pone.0352998.r003

**Authors:** Chih-Wei Tseng

20 Apr 2026

<div>PONE-D-25-66438R1

Prevalence and risk factors for colorectal neoplasia in asymptomatic Vietnamese undergoing self-funded screening colonoscopy

PLOS One

Dear Dr. Quach,

Thank you for submitting your manuscript to PLOS ONE. After careful consideration, we feel that it has merit but does not fully meet PLOS ONE’s publication criteria as it currently stands. Therefore, we invite you to submit a revised version of the manuscript that addresses the points raised during the review process.

Please submit your revised manuscript by Jun 04 2026 11:59PM. If you will need more time than this to complete your revisions, please reply to this message or contact the journal office at plosone@plos.org. When you're ready to submit your revision, log on to https://www.editorialmanager.com/pone/ and select the 'Submissions Needing Revision' folder to locate your manuscript file.

Please include the following items when submitting your revised manuscript:

- A letter that responds to each point raised by the academic editor and reviewer(s). You should upload this letter as a separate file labeled 'Response to Reviewers'.
- A marked-up copy of your manuscript that highlights changes made to the original version. You should upload this as a separate file labeled 'Revised Manuscript with Track Changes'.
- An unmarked version of your revised paper without tracked changes. You should upload this as a separate file labeled 'Manuscript'.

If you would like to make changes to your financial disclosure, please include your updated statement in your cover letter. Guidelines for resubmitting your figure files are available below the reviewer comments at the end of this letter.

If applicable, we recommend that you deposit your laboratory protocols in protocols.io to enhance the reproducibility of your results. Protocols.io assigns your protocol its own identifier (DOI) so that it can be cited independently in the future. For instructions see: https://journals.plos.org/plosone/s/submission-guidelines#loc-laboratory-protocols. Additionally, PLOS ONE offers an option for publishing peer-reviewed Lab Protocol articles, which describe protocols hosted on protocols.io. Read more information on sharing protocols at https://plos.org/protocols?utm_medium=editorial-email&utm_source=authorletters&utm_campaign=protocols.

As the corresponding author, your ORCID iD is verified in the submission system and will appear in the published article. PLOS supports the use of ORCID, and we encourage all coauthors to register for an ORCID iD and use it as well. Please encourage your coauthors to verify their ORCID iD within the submission system before final acceptance, as unverified ORCID iDs will not appear in the published article. *Only*  the individual author can complete the verification step; PLOS staff *cannot*  verify ORCID iDs on behalf of authors.

We look forward to receiving your revised manuscript.

Kind regards,

Chih-Wei Tseng

Academic Editor

PLOS One

Journal Requirements:

1. If the reviewer comments include a recommendation to cite specific previously published works, please review and evaluate these publications to determine whether they are relevant and should be cited. There is no requirement to cite these works unless the editor has indicated otherwise.

2. Please review your reference list to ensure that it is complete and correct. If you have cited papers that have been retracted, please include the rationale for doing so in the manuscript text, or remove these references and replace them with relevant current references. Any changes to the reference list should be mentioned in the rebuttal letter that accompanies your revised manuscript. If you need to cite a retracted article, indicate the article’s retracted status in the References list and also include a citation and full reference for the retraction notice.

[Note: HTML markup is below. Please do not edit.]

Reviewers' comments:

Reviewer's Responses to Questions

**Comments to the Author**

1. If the authors have adequately addressed your comments raised in a previous round of review and you feel that this manuscript is now acceptable for publication, you may indicate that here to bypass the “Comments to the Author” section, enter your conflict of interest statement in the “Confidential to Editor” section, and submit your "Accept" recommendation.

Reviewer #1: All comments have been addressed

Reviewer #2: All comments have been addressed

**********

2. Is the manuscript technically sound, and do the data support the conclusions?

The manuscript must describe a technically sound piece of scientific research with data that supports the conclusions. Experiments must have been conducted rigorously, with appropriate controls, replication, and sample sizes. The conclusions must be drawn appropriately based on the data presented.

Reviewer #1: Yes

Reviewer #2: Yes

**********

3. Has the statistical analysis been performed appropriately and rigorously?

Reviewer #1: Yes

Reviewer #2: Yes

**********

4. Have the authors made all data underlying the findings in their manuscript fully available?

The PLOS Data policy requires authors to make all data underlying the findings described in their manuscript fully available without restriction, with rare exception (please refer to the Data Availability Statement in the manuscript PDF file). The data should be provided as part of the manuscript or its supporting information, or deposited to a public repository. For example, in addition to summary statistics, the data points behind means, medians and variance measures should be available. If there are restrictions on publicly sharing data—e.g. participant privacy or use of data from a third party—those must be specified.

Reviewer #1: Yes

Reviewer #2: Yes

**********

5. Is the manuscript presented in an intelligible fashion and written in standard English?

PLOS ONE does not copyedit accepted manuscripts, so the language in submitted articles must be clear, correct, and unambiguous. Any typographical or grammatical errors should be corrected at revision, so please note any specific errors here.

Reviewer #1: Yes

Reviewer #2: Yes

**********

6. Review Comments to the Author

Please use the space provided to explain your answers to the questions above. You may also include additional comments for the author, including concerns about dual publication, research ethics, or publication ethics. (Please upload your review as an attachment if it exceeds 20,000 characters)

Reviewer #1: Generalizability and the "Screening-Attending" Population:

The authors have commendably re-framed their population as "screening-attending" or "self-funded screening" throughout the manuscript and have strengthened the limitations section to explicitly address selection bias. This is a major improvement.

Remaining Concern: While the term "screening-attending" is more accurate, the Discussion and Conclusion could still be misconstrued by a casual reader. The findings are highly valuable for describing this specific cohort and for generating hypotheses, but they should not be interpreted as directly applicable to the "average-risk Vietnamese population" at large. The authors have done a good job of inserting cautionary language, but the final conclusion could be sharpened to reinforce this point. The statement "CRN was prevalent among asymptomatic Vietnamese adults undergoing self-funded screening colonoscopy" is precise and should be the central takeaway.

Inference on Screening Age Threshold:

The authors have correctly softened their language around the optimal screening age, moving from directive statements to hypothesis-generating ones ("raise the hypothesis," "warrants further investigation"). This is appropriate and responsible.

Remaining Concern: The discussion of the age threshold (lines 276-281) is well-balanced. However, it is important to reiterate that the finding of a significant risk at age ≥40 in this cohort does not, by itself, provide evidence for the efficacy or cost-effectiveness of starting screening at that age in the general population. The authors acknowledge the need for population-based studies, which is sufficient. No further action is needed, as the current phrasing is appropriate.

Handling of the NICE Type I Discordance:

The authors' response to the discordance between endoscopic NICE I classification and histological CRN is reasonable, citing the challenge of small lesions. The addition of the reference [27] supports this.

Remaining Concern: While the explanation is plausible, the manuscript could briefly mention that this discordance, while not affecting the final pathology-based diagnosis, highlights a limitation of real-time optical diagnosis in this setting. The current text in the Discussion (lines 259-264) adequately frames this as a known challenge.

Minor Comments

Abstract - Conclusions: The abstract conclusion ("Advanced age, overweight, and family history of colorectal cancer were independent factors associated with CRN.") is accurate and well-stated. It avoids overgeneralization.

Introduction (Lines 89-95): The rationale for including adults aged ≥18 years is now much clearer and well-justified by the opportunistic screening context in Vietnam. This effectively addresses the previous concern about a post-hoc inclusion.

Methods - Exposure Measurement (Lines 336-344): The authors have significantly strengthened the limitations section by explicitly acknowledging the crude categorization of smoking and alcohol and the lack of data on diet, physical activity, and metabolic factors. This transparency is excellent and crucial for interpreting the risk estimates.

Results - Table 1: The corrected Table 1 is now accurate. The use of superscripts (a, b, c, d) for age group p-values is a clear and helpful addition.

Discussion - Advanced CRN Associations (Lines 318-324): The authors provide a balanced discussion on why BMI and family history were not significant for advanced CRN, citing both statistical power and potential biological differences. This is a fair and nuanced interpretation.

Use of AI: The declaration of AI use for language editing is transparent and follows best practices.

Reviewer #2: Most areas of concern from the title, abstract, introduction, method, result and discussion have been addressed, and others were presented as limitations.

**********

7. PLOS authors have the option to publish the peer review history of their article (what does this mean?). If published, this will include your full peer review and any attached files.

If you choose “no”, your identity will remain anonymous but your review may still be made public.

**Do you want your identity to be public for this peer review?**  For information about this choice, including consent withdrawal, please see our Privacy Policy.

Reviewer #1: No

Reviewer #2: **Yes:**  PROF ANYANWU GODSON EMEKA

**********

[NOTE: If reviewer comments were submitted as an attachment file, they will be attached to this email and accessible via the submission site. Please log into your account, locate the manuscript record, and check for the action link "View Attachments". If this link does not appear, there are no attachment files.]

To ensure your figures meet our technical requirements, please review our figure guidelines: https://journals.plos.org/plosone/s/figures

You may also use PLOS’s free figure tool, NAAS, to help you prepare publication quality figures: https://journals.plos.org/plosone/s/figures#loc-tools-for-figure-preparation.

NAAS will assess whether your figures meet our technical requirements by comparing each figure against our figure specifications.

# Author comment: Author response to Decision Letter 2

**DOI:** 10.1371/journal.pone.0352998.r004

21 Apr 2026

April 21, 2026

Dear Editor,

We would like to thank you very much for your kindness in allowing us to revise our manuscript. We also thank the reviewers for their constructive comments. The manuscript has been revised accordingly, and the revised sections in the resubmitted manuscript have been highlighted in blue. We would like to respond to your comments as follows.

Response to Reviewer 1

Comment 1. Generalizability and the "Screening-Attending" Population

Remaining Concern: While the term "screening-attending" is more accurate, the Discussion and Conclusion could still be misconstrued by a casual reader. The findings are highly valuable for describing this specific cohort and for generating hypotheses, but they should not be interpreted as directly applicable to the "average-risk Vietnamese population" at large. The authors have done a good job of inserting cautionary language, but the final conclusion could be sharpened to reinforce this point. The statement "CRN was prevalent among asymptomatic Vietnamese adults undergoing self-funded screening colonoscopy" is precise and should be the central takeaway.

Reply:

We thank the reviewer for this thoughtful comment. We agree that, despite our previous revisions, the findings could still be interpreted as being applicable to the general Vietnamese population. To address this, we have revised the Conclusion to more clearly state that our results reflect a self-selected, screening-attending population, and should not be directly generalized to the average-risk population (page 19, lines 360-361).

“In conclusion, CRN was prevalent among asymptomatic Vietnamese adults undergoing self-funded screening colonoscopy. Age ≥ 40 years, overweight status, and family history of CRC were independently associated with CRN in this population. These findings should be interpreted with caution and may not be directly generalizable to the average-risk Vietnamese population.”

Comment 2. Inference on Screening Age Threshold:

Remaining Concern: The discussion of the age threshold (lines 276-281) is well-balanced. However, it is important to reiterate that the finding of a significant risk at age ≥ 40 in this cohort does not, by itself, provide evidence for the efficacy or cost-effectiveness of starting screening at that age in the general population. The authors acknowledge the need for population-based studies, which is sufficient. No further action is needed, as the current phrasing is appropriate.

Reply:

We agree with the reviewer’s point. The observed association at age ≥40 years in this cohort should not be interpreted as evidence for screening effectiveness or policy change. As already revised in the previous version, we have framed this finding as hypothesis-generating and emphasized the need for population-based studies. We have kept the current wording, as it already reflects this limitation.

Comment 2. Handling of the NICE Type I Discordance:

Remaining Concern: While the explanation is plausible, the manuscript could briefly mention that this discordance, while not affecting the final pathology-based diagnosis, highlights a limitation of real-time optical diagnosis in this setting. The current text in the Discussion (lines 259-264) adequately frames this as a known challenge.

Reply:

We thank the reviewer for this constructive comment. We have added a sentence in the Discussion to clarify that this discordance reflects a limitation of real-time optical diagnosis, particularly in small lesions (page 15, lines 264-265).

“There may be discrepancies between endoscopic appearance and pathological diagnosis, particularly in small lesions where optical differentiation between diminutive hyperplastic, serrated, and low-grade neoplastic lesions can be challenging [27]. This also highlights a limitation of real-time optical diagnosis in routine clinical practice.”

Minor Comments

Reply:

We thank the reviewer for the positive feedback on the revised manuscript. We are glad that the previous concerns regarding the abstract, introduction, methods, results, and discussion have been adequately addressed. No further changes were made in response to these points.

Response to Reviewer 2

Comment. Most areas of concern from the title, abstract, introduction, method, result and discussion have been addressed, and others were presented as limitations.

Reply:

We thank the reviewer for the careful review and for noting that the major concerns have been addressed. We appreciate the positive assessment of the revised manuscript.

Once again, we would like to thank you for your time, and we greatly appreciate your insights that helped us refine the manuscript.

With the warmest regards,

On behalf of the authors

Duc Quach, MD; PhD

Department of Internal Medicine and Gastro-Hepato Integrated Research Team (GHIRT-002.TCM2025), School of Medicine, University of Medicine and Pharmacy at Ho Chi Minh City, Vietnam

217 Hong Bang Street, Cho Lon Ward, Ho Chi Minh City, Vietnam

Tel: +84-91-808-025

Email: drquachtd@ump.edu.vn

Attachment Submitted filename: point-to-ponit reply- R2.docx

# Peer review: Decision Letter 2

**DOI:** 10.1371/journal.pone.0352998.r005

**Authors:** Chih-Wei Tseng

1 May 2026

PONE-D-25-66438R2

Prevalence and risk factors for colorectal neoplasia in asymptomatic Vietnamese undergoing self-funded screening colonoscopy

PLOS One

Dear Dr. Quach,

Thank you for submitting your manuscript to PLOS ONE. After careful consideration, we feel that it has merit but does not fully meet PLOS ONE’s publication criteria as it currently stands. Therefore, we invite you to submit a revised version of the manuscript that addresses the points raised during the review process.

Please submit your revised manuscript by Jun 15 2026 11:59PM. If you will need more time than this to complete your revisions, please reply to this message or contact the journal office at plosone@plos.org. When you're ready to submit your revision, log on to https://www.editorialmanager.com/pone/ and select the 'Submissions Needing Revision' folder to locate your manuscript file.

Please include the following items when submitting your revised manuscript:

- A letter that responds to each point raised by the academic editor and reviewer(s). You should upload this letter as a separate file labeled 'Response to Reviewers'.
- A marked-up copy of your manuscript that highlights changes made to the original version. You should upload this as a separate file labeled 'Revised Manuscript with Track Changes'.
- An unmarked version of your revised paper without tracked changes. You should upload this as a separate file labeled 'Manuscript'.

If you would like to make changes to your financial disclosure, please include your updated statement in your cover letter. Guidelines for resubmitting your figure files are available below the reviewer comments at the end of this letter.

If applicable, we recommend that you deposit your laboratory protocols in protocols.io to enhance the reproducibility of your results. Protocols.io assigns your protocol its own identifier (DOI) so that it can be cited independently in the future. For instructions see: https://journals.plos.org/plosone/s/submission-guidelines#loc-laboratory-protocols. Additionally, PLOS ONE offers an option for publishing peer-reviewed Lab Protocol articles, which describe protocols hosted on protocols.io. Read more information on sharing protocols at https://plos.org/protocols?utm_medium=editorial-email&utm_source=authorletters&utm_campaign=protocols.

As the corresponding author, your ORCID iD is verified in the submission system and will appear in the published article. PLOS supports the use of ORCID, and we encourage all coauthors to register for an ORCID iD and use it as well. Please encourage your coauthors to verify their ORCID iD within the submission system before final acceptance, as unverified ORCID iDs will not appear in the published article. *Only*  the individual author can complete the verification step; PLOS staff *cannot*  verify ORCID iDs on behalf of authors.

We look forward to receiving your revised manuscript.

Kind regards,

Chih-Wei Tseng

Academic Editor

PLOS One

Journal Requirements:

1. If the reviewer comments include a recommendation to cite specific previously published works, please review and evaluate these publications to determine whether they are relevant and should be cited. There is no requirement to cite these works unless the editor has indicated otherwise.

2. Please review your reference list to ensure that it is complete and correct. If you have cited papers that have been retracted, please include the rationale for doing so in the manuscript text, or remove these references and replace them with relevant current references. Any changes to the reference list should be mentioned in the rebuttal letter that accompanies your revised manuscript. If you need to cite a retracted article, indicate the article’s retracted status in the References list and also include a citation and full reference for the retraction notice.

[Note: HTML markup is below. Please do not edit.]

Reviewers' comments:

Reviewer's Responses to Questions

**Comments to the Author**

1. If the authors have adequately addressed your comments raised in a previous round of review and you feel that this manuscript is now acceptable for publication, you may indicate that here to bypass the “Comments to the Author” section, enter your conflict of interest statement in the “Confidential to Editor” section, and submit your "Accept" recommendation.

Reviewer #1: All comments have been addressed

**********

2. Is the manuscript technically sound, and do the data support the conclusions?

The manuscript must describe a technically sound piece of scientific research with data that supports the conclusions. Experiments must have been conducted rigorously, with appropriate controls, replication, and sample sizes. The conclusions must be drawn appropriately based on the data presented.

Reviewer #1: Partly

**********

3. Has the statistical analysis been performed appropriately and rigorously?

Reviewer #1: No

**********

4. Have the authors made all data underlying the findings in their manuscript fully available?

The PLOS Data policy requires authors to make all data underlying the findings described in their manuscript fully available without restriction, with rare exception (please refer to the Data Availability Statement in the manuscript PDF file). The data should be provided as part of the manuscript or its supporting information, or deposited to a public repository. For example, in addition to summary statistics, the data points behind means, medians and variance measures should be available. If there are restrictions on publicly sharing data—e.g. participant privacy or use of data from a third party—those must be specified.

Reviewer #1: Yes

**********

5. Is the manuscript presented in an intelligible fashion and written in standard English?

PLOS ONE does not copyedit accepted manuscripts, so the language in submitted articles must be clear, correct, and unambiguous. Any typographical or grammatical errors should be corrected at revision, so please note any specific errors here.

Reviewer #1: Yes

**********

6. Review Comments to the Author

Please use the space provided to explain your answers to the questions above. You may also include additional comments for the author, including concerns about dual publication, research ethics, or publication ethics. (Please upload your review as an attachment if it exceeds 20,000 characters)

Reviewer #1: 2. Severe selection bias undermines generalizability (unchanged from prior review)

The study recruits from a single private polyclinic in Ho Chi Minh City, where participants pay out-of-pocket for colonoscopy. These individuals are likely wealthier, more health-conscious, and have different risk profiles than the general Vietnamese population. The authors have added cautionary language to the conclusion, but this does not fix the fundamental design limitation. For a high-impact journal, a single-center, self-selected cohort cannot support meaningful prevalence estimates for the broader population – even as a “hypothesis-generating” study, the bias is too large.

3. Post-hoc age dichotomization is a serious methodological flaw

The authors dichotomized age at ≥40 years for multivariable analysis without pre-specification, then used the data to justify this cutoff. This is a classic example of data-driven dichotomization that inflates Type I error and yields unstable estimates. The correct approach would have been to model age flexibly (splines, fractional polynomials) or use clinically justified cutoffs from external literature. The authors’ stated reason – avoiding collinearity and preserving power – does not excuse the post-hoc decision.

4. Inadequate control for confounding

The multivariable model adjusts for only 6 variables (age, sex, BMI, family history, smoking, alcohol). Numerous known CRN risk factors are omitted: diabetes, physical inactivity, red/processed meat intake, NSAID use, dietary fiber, and socioeconomic status. Given the self-selected nature of the cohort, residual confounding is likely substantial. The authors do not provide any sensitivity analyses (e.g., E-values, propensity score approaches) to assess robustness.

5. Underpowered analysis for advanced CRN

Only 64 participants had advanced CRN. The multivariable model for this outcome includes 6 predictors, yielding an events-per-variable ratio of ~10.7 – at the absolute minimum acceptable threshold. The resulting confidence intervals are very wide (e.g., age OR 5.20, 95% CI 1.22–22.11), and the null findings for BMI and family history should not be interpreted as evidence of no association. The authors acknowledge this limitation, but the analysis remains exploratory and should not be presented as definitive.

6. Unvalidated definition of “asymptomatic”

Asymptomatic status was determined by unstructured clinical interviews, not by validated instruments (e.g., Rome criteria). No information is provided on inter-rater reliability or the specific questions asked. Recall bias is likely, and some participants may have had mild or intermittent symptoms that were not captured. The prevalence estimate could be meaningfully affected by misclassification.

7. NICE type I discordance highlights quality issue

The authors note that 15.3% of lesions were endoscopically classified as NICE type I (suggesting hyperplastic) but were histologically confirmed as neoplasia. This discordance rate is higher than reported in high-quality optical diagnosis studies. It raises questions about the endoscopists’ real-time diagnostic accuracy and whether all participants received equivalent quality examination. The authors add a sentence acknowledging this as a limitation, but the high discordance should have triggered a more thorough quality control review.

**********

7. PLOS authors have the option to publish the peer review history of their article (what does this mean?). If published, this will include your full peer review and any attached files.

If you choose “no”, your identity will remain anonymous but your review may still be made public.

**Do you want your identity to be public for this peer review?**  For information about this choice, including consent withdrawal, please see our Privacy Policy.

Reviewer #1: No

**********

[NOTE: If reviewer comments were submitted as an attachment file, they will be attached to this email and accessible via the submission site. Please log into your account, locate the manuscript record, and check for the action link "View Attachments". If this link does not appear, there are no attachment files.]

To ensure your figures meet our technical requirements, please review our figure guidelines: https://journals.plos.org/plosone/s/figures

You may also use PLOS’s free figure tool, NAAS, to help you prepare publication quality figures: https://journals.plos.org/plosone/s/figures#loc-tools-for-figure-preparation.

NAAS will assess whether your figures meet our technical requirements by comparing each figure against our figure specifications.

# Author comment: Author response to Decision Letter 3

**DOI:** 10.1371/journal.pone.0352998.r006

3 May 2026

May 03, 2026

Dear Editor,

We would like to thank you very much for your kindness in allowing us to revise our manuscript. We also thank the reviewers for their constructive comments. The manuscript has been revised accordingly, and the revised sections in the resubmitted manuscript have been highlighted in blue. We would like to respond to your comments as follows.

Response to Reviewer 1

Comment 1. Severe selection bias undermines generalizability (unchanged from prior review).

The study recruits from a single private polyclinic in Ho Chi Minh City, where participants pay out-of-pocket for colonoscopy. These individuals are likely wealthier, more health-conscious, and have different risk profiles than the general Vietnamese population. The authors have added cautionary language to the conclusion, but this does not fix the fundamental design limitation. For a high-impact journal, a single-center, self-selected cohort cannot support meaningful prevalence estimates for the broader population – even as a “hypothesis-generating” study, the bias is too large.

Reply:

Thank you very much for your comment. We fully agree that our study population represents a self-selected, screening-attending cohort from a private clinical setting, and therefore differs from the general Vietnamese population in terms of socioeconomic and health-seeking characteristics. However, we respectfully note that our primary objective was not to estimate population-level prevalence, but rather to describe the burden and associated risk factors of colorectal neoplasia within this increasingly common real-world screening context in Vietnam, where organized population-based screening programs are not yet established.

We have further revised the Discussion to explicitly emphasize that our findings are specific to this screening-attending population and should not be generalized to the average-risk population (page 18, lines 343-346).

“Importantly, our study was not designed to provide population-level prevalence estimates, but rather to describe the burden and associated risk factors of CRN within a real-world screening-attending population, which is common in Vietnam in the absence of organized national screening programs.”

Comment 2. Post-hoc age dichotomization is a serious methodological flaw

The authors dichotomized age at ≥40 years for multivariable analysis without pre-specification, then used the data to justify this cutoff. This is a classic example of data-driven dichotomization that inflates Type I error and yields unstable estimates. The correct approach would have been to model age flexibly (splines, fractional polynomials) or use clinically justified cutoffs from external literature. The authors’ stated reason – avoiding collinearity and preserving power – does not excuse the post-hoc decision.

Reply:

We thank the reviewer for this important methodological comment. We agree that dichotomization of continuous variables may introduce limitations, particularly if interpreted as data-driven. We would like to clarify, however, that the selection of an age threshold of ≥40 years in our analysis was informed by prior evidence and regional clinical context, rather than being derived solely from our dataset. Specifically, previous studies in Vietnam have reported a substantial proportion of early-onset colorectal cancer, suggesting that colorectal neoplasia may occur at a younger age compared with Western populations [4]. In addition, screening practices in some Asian countries, such as Japan, begin at age 40 [10]. Furthermore, a large prospective colonoscopy study in asymptomatic Korean adults—representing a screening-attending population similar to our study setting—demonstrated that colorectal neoplasia occurs across a broad age range, including individuals younger than 50 years [28]. Taken together, these findings provide a clinical and epidemiological rationale for exploring a lower age threshold in this context, rather than relying exclusively on conventional cutoffs derived from Western populations.

To address the reviewer’s concern, we have revised the Methods and Discussion sections to clarify this rationale and have incorporated supporting evidence from studies in Vietnam, Japan, and Korea (pages 12-13, lines 217-221; page 15, lines 270-275; and page 16, lines 289-293).

“We categorized age using a cutoff of ≥ 40 years for multivariable analysis. This threshold was selected based on prior evidence suggesting an earlier onset of CRN in Asian populations, including data from Vietnam [4], as well as regional screening practices and guidelines [10]. This approach also facilitated a simplified and interpretable model.”

“Evidence from Vietnam has demonstrated a substantial proportion of early-onset RC [4], while screening practices in some Asian countries, such as Japan, begin at age 40 [10]. In addition, a large prospective colonoscopy study in asymptomatic Korean adults—reflecting a screening-attending population—has shown that CRN may occur before the age of 50 [28]. Together, these findings support the rationale for exploring a lower age threshold in this context.”

“Therefore, these data suggest a potential burden of CRC in younger adults within screening-attending populations. These observations highlight the need for further investigation into age-specific risk stratification in this context. However, this observation should be interpreted as hypothesis-generating, and confirmation from large-scale, population-based studies is required before informing national policy.”

References:

4. Quach DT, Nguyen OT. Clinical, endoscopic and pathogical characteristics of early-onset colorectal cancer in Vietnamese. Asian Pac J Cancer Prev. 2012;13(5):1767-70. Epub 2012/08/21. doi: 10.7314/apjcp.2012.13.5.1767. PubMed PMID: 22901119.

10. Saito Y, Oka S, Kawamura T, Shimoda R, Sekiguchi M, Tamai N, et al. Colonoscopy screening and surveillance guidelines. Dig Endosc. 2021;33(4):486-519. Epub 2021/03/14. doi: 10.1111/den.13972. PubMed PMID: 33713493.

28. Park HW, Byeon JS, Yang SK, Kim HS, Kim WH, Kim TI, et al. Colorectal Neoplasm in Asymptomatic Average-risk Koreans: The KASID Prospective Multicenter Colonoscopy Survey. Gut Liver. 2009;3(1):35-40. Epub 2009/03/01. doi: 10.5009/gnl.2009.3.1.35. PubMed PMID: 20479899; PubMed Central PMCID: PMCPMC2871563.

Comment 3. Inadequate control for confounding

The multivariable model adjusts for only 6 variables (age, sex, BMI, family history, smoking, alcohol). Numerous known CRN risk factors are omitted: diabetes, physical inactivity, red/processed meat intake, NSAID use, dietary fiber, and socioeconomic status. Given the self-selected nature of the cohort, residual confounding is likely substantial. The authors do not provide any sensitivity analyses (e.g., E-values, propensity score approaches) to assess robustness.

Reply:

We thank the reviewer for this important comment. We agree that residual confounding cannot be completely excluded in our study, given its observational design and the limited availability of certain variables such as dietary factors, physical activity, metabolic comorbidities (e.g., diabetes), and socioeconomic status. However, we included key established risk factors that have been consistently reported in the literature and were feasible to collect in routine clinical practice. Notably, a large prospective multinational study in asymptomatic Asian populations identified advancing age, male sex, and family history of colorectal cancer as major risk factors for colorectal neoplasia, which are consistent with the variables included in our model [25].

To address the reviewer’s concern, we have expanded the Discussion and Limitation section to more explicitly acknowledge the potential impact of unmeasured confounders and to clarify that the observed associations should be interpreted with caution. We agree that future studies incorporating more comprehensive exposure assessment and additional lifestyle and metabolic variables would be valuable to further refine risk estimation (page 15, lines 277-280 and page 19, lines 360-362).

“These findings are consistent with those from a large prospective multinational study in asymptomatic Asian populations, which identified advanced age, male sex, and family history of CRC as key risk factors for CRN [25].”

“Therefore, the observed associations should be interpreted with caution, as unmeasured confounding factors may have influenced the results.”

References:

25. Byeon JS, Yang SK, Kim TI, Kim WH, Lau JY, Leung WK, et al. Colorectal neoplasm in asymptomatic Asians: a prospective multinational multicenter colonoscopy survey. Gastrointest Endosc. 2007;65(7):1015-22. Epub 2007/05/29. doi: 10.1016/j.gie.2006.12.065. PubMed PMID: 17531636.

Comment 4. Underpowered analysis for advanced CRN

Only 64 participants had advanced CRN. The multivariable model for this outcome includes 6 predictors, yielding an events-per-variable ratio of ~10.7 – at the absolute minimum acceptable threshold. The resulting confidence intervals are very wide (e.g., age OR 5.20, 95% CI 1.22–22.11), and the null findings for BMI and family history should not be interpreted as evidence of no association. The authors acknowledge this limitation, but the analysis remains exploratory and should not be presented as definitive.

Reply:

Thank you very much for your comment. We agree that the number of advanced CRN cases was relatively limited, which may affect the precision of multivariable estimates. We have emphasized in the revised manuscript that analyses related to advanced CRN should be considered exploratory and that the absence of statistical significance should not be interpreted as evidence of no association (pages 17-18, lines 334-336).

“Accordingly, the absence of statistical significance for certain variables should not be interpreted as evidence of no association, but rather as a reflection of limited statistical power.”

Comment 5. Unvalidated definition of “asymptomatic”

Asymptomatic status was determined by unstructured clinical interviews, not by validated instruments (e.g., Rome criteria). No information is provided on inter-rater reliability or the specific questions asked. Recall bias is likely, and some participants may have had mild or intermittent symptoms that were not captured. The prevalence estimate could be meaningfully affected by misclassification.

Reply:

Thank you very much for your comment. We acknowledge that asymptomatic status was not defined using a formally validated instrument. This approach reflects routine clinical practice in screening settings, where symptom assessment is typically conducted through physician-led interviews. We have addressed the potential for misclassification in the limitation section of the revised manuscript (page 18, lines 351-355).

“Second, although asymptomatic status was determined through structured clinical assessment by experienced gastroenterologists—as is typical in routine screening practice—it was not based on a formally validated instrument, which may introduce some degree of symptom misclassification and potentially influence prevalence estimates.”

Comment 6. NICE type I discordance highlights quality issue

The authors note that 15.3% of lesions were endoscopically classified as NICE type I (suggesting hyperplastic) but were histologically confirmed as neoplasia. This discordance rate is higher than reported in high-quality optical diagnosis studies. It raises questions about the endoscopists’ real-time diagnostic accuracy and whether all participants received equivalent quality examination. The authors add a sentence acknowledging this as a limitation, but the high discordance should have triggered a more thorough quality control review.

Reply:

We thank you for this important observation. We agree that the observed discordance between NICE classification and histopathological findings may raise concerns regarding real-time optical diagnosis. However, this level of discordance is consistent with known limitations of optical diagnosis, particularly for small or subtle lesions in routine clinical practice. Importantly, all colonoscopies in our study were performed by experienced endoscopists who met established quality indicators, including an adenoma detection rate exceeding 30%, suggesting that overall examination quality was maintained. In addition, lesions suspected to be non-neoplastic were generally resected and subjected to histopathological evaluation, which may reduce the potential impact of optical misclassification on study outcomes.

To address the reviewer’s concern, we have expanded the Discussion to clarify that this discordance likely reflects inherent limitations of optical diagnosis rather than a systematic quality issue, and that the use of histopathological confirmation mitigates its potential impact on study outcomes (page 15, lines 265-269).

“However, all colonoscopies were performed by experienced endoscopists with established quality indicators (adenoma detection rate >30%), suggesting that overall examination quality was maintained. In addition, lesions suspected to be non-neoplastic were generally resected and histologically examined, which may mitigate the impact of optical misclassification on study outcomes.”

Once again, we would like to thank you for your time, and we greatly appreciate your insights that helped us refine the manuscript.

With the warmest regards,

On behalf of the authors

Duc Quach, MD; PhD

Department of Internal Medicine and Gastro-Hepato Integrated Research Team (GHIRT-002.TCM2025), School of Medicine, University of Medicine and Pharmacy at Ho Chi Minh City, Vietnam

217 Hong Bang Street, Cho Lon Ward, Ho Chi Minh City, Vietnam

Tel: +84-91-808-025

Email: drquachtd@ump.edu.vn

Attachment Submitted filename: Response - Plos One - R3 FINAL.docx

# Peer review: Decision Letter 3

**DOI:** 10.1371/journal.pone.0352998.r007

**Authors:** Chih-Wei Tseng

15 May 2026

PONE-D-25-66438R3

Prevalence and risk factors for colorectal neoplasia in asymptomatic Vietnamese undergoing self-funded screening colonoscopy

PLOS One

Dear Dr. Quach,

Thank you for submitting your manuscript to PLOS ONE. After careful consideration, we feel that it has merit but does not fully meet PLOS ONE’s publication criteria as it currently stands. Therefore, we invite you to submit a revised version of the manuscript that addresses the points raised during the review process.

Please submit your revised manuscript by Jun 29 2026 11:59PM. If you will need more time than this to complete your revisions, please reply to this message or contact the journal office at plosone@plos.org. When you're ready to submit your revision, log on to https://www.editorialmanager.com/pone/ and select the 'Submissions Needing Revision' folder to locate your manuscript file.

Please include the following items when submitting your revised manuscript:

- A letter that responds to each point raised by the academic editor and reviewer(s). You should upload this letter as a separate file labeled 'Response to Reviewers'.
- A marked-up copy of your manuscript that highlights changes made to the original version. You should upload this as a separate file labeled 'Revised Manuscript with Track Changes'.
- An unmarked version of your revised paper without tracked changes. You should upload this as a separate file labeled 'Manuscript'.

If you would like to make changes to your financial disclosure, please include your updated statement in your cover letter. Guidelines for resubmitting your figure files are available below the reviewer comments at the end of this letter.

If applicable, we recommend that you deposit your laboratory protocols in protocols.io to enhance the reproducibility of your results. Protocols.io assigns your protocol its own identifier (DOI) so that it can be cited independently in the future. For instructions see: https://journals.plos.org/plosone/s/submission-guidelines#loc-laboratory-protocols. Additionally, PLOS ONE offers an option for publishing peer-reviewed Lab Protocol articles, which describe protocols hosted on protocols.io. Read more information on sharing protocols at https://plos.org/protocols?utm_medium=editorial-email&utm_source=authorletters&utm_campaign=protocols.

As the corresponding author, your ORCID iD is verified in the submission system and will appear in the published article. PLOS supports the use of ORCID, and we encourage all coauthors to register for an ORCID iD and use it as well. Please encourage your coauthors to verify their ORCID iD within the submission system before final acceptance, as unverified ORCID iDs will not appear in the published article. *Only*  the individual author can complete the verification step; PLOS staff *cannot*  verify ORCID iDs on behalf of authors.

We look forward to receiving your revised manuscript.

Kind regards,

Chih-Wei Tseng

Academic Editor

PLOS One

Journal Requirements:

1. If the reviewer comments include a recommendation to cite specific previously published works, please review and evaluate these publications to determine whether they are relevant and should be cited. There is no requirement to cite these works unless the editor has indicated otherwise.

2. Please review your reference list to ensure that it is complete and correct. If you have cited papers that have been retracted, please include the rationale for doing so in the manuscript text, or remove these references and replace them with relevant current references. Any changes to the reference list should be mentioned in the rebuttal letter that accompanies your revised manuscript. If you need to cite a retracted article, indicate the article’s retracted status in the References list and also include a citation and full reference for the retraction notice.

[Note: HTML markup is below. Please do not edit.]

Reviewer's Responses to Questions

**Comments to the Author**

1. If the authors have adequately addressed your comments raised in a previous round of review and you feel that this manuscript is now acceptable for publication, you may indicate that here to bypass the “Comments to the Author” section, enter your conflict of interest statement in the “Confidential to Editor” section, and submit your "Accept" recommendation.

Reviewer #1: All comments have been addressed

**********

2. Is the manuscript technically sound, and do the data support the conclusions?

The manuscript must describe a technically sound piece of scientific research with data that supports the conclusions. Experiments must have been conducted rigorously, with appropriate controls, replication, and sample sizes. The conclusions must be drawn appropriately based on the data presented.

Reviewer #1: Yes

**********

3. Has the statistical analysis been performed appropriately and rigorously?

Reviewer #1: Yes

**********

4. Have the authors made all data underlying the findings in their manuscript fully available?

The PLOS Data policy requires authors to make all data underlying the findings described in their manuscript fully available without restriction, with rare exception (please refer to the Data Availability Statement in the manuscript PDF file). The data should be provided as part of the manuscript or its supporting information, or deposited to a public repository. For example, in addition to summary statistics, the data points behind means, medians and variance measures should be available. If there are restrictions on publicly sharing data—e.g. participant privacy or use of data from a third party—those must be specified.

Reviewer #1: Yes

**********

5. Is the manuscript presented in an intelligible fashion and written in standard English?

PLOS ONE does not copyedit accepted manuscripts, so the language in submitted articles must be clear, correct, and unambiguous. Any typographical or grammatical errors should be corrected at revision, so please note any specific errors here.

Reviewer #1: Yes

**********

6. Review Comments to the Author

Please use the space provided to explain your answers to the questions above. You may also include additional comments for the author, including concerns about dual publication, research ethics, or publication ethics. (Please upload your review as an attachment if it exceeds 20,000 characters)

Reviewer #1: 1. Severe Selection Bias – Findings Not Generalizable

The study recruits from a single private polyclinic in Ho Chi Minh City where participants pay out-of-pocket for colonoscopy. These individuals are inherently wealthier, more health-conscious, and likely have different risk profiles (e.g., lower smoking rates, higher health literacy, different dietary patterns) than the general Vietnamese population. The authors acknowledge this limitation but continue to frame the findings as relevant to "asymptomatic Vietnamese adults" – which is misleading.

Critical issue: The prevalence estimates (26.2% overall CRN, 9.0% advanced CRN) cannot be extrapolated to the general population. The authors correctly note that the study was "not designed to provide population-level prevalence estimates," yet the abstract, results, and discussion repeatedly imply broader relevance. For a high-impact journal, a single-center, self-selected cohort cannot support meaningful prevalence estimates – even as a “hypothesis-generating” study, the bias is too large to justify any conclusion about the Vietnamese population.

Required action:

Revise the title and abstract to explicitly state that findings are from a self-selected screening-attending cohort in a private clinical setting, not representative of asymptomatic Vietnamese adults.

Remove or substantially tone down statements implying generalizability (e.g., "CRN was prevalent in asymptomatic Vietnamese adults" should become "CRN was prevalent in this self-selected screening cohort").

Add a dedicated paragraph in the Discussion quantifying the expected direction and magnitude of selection bias.

2. Post-hoc Age Dichotomization – Methodologically Flawed

The authors dichotomized age at ≥40 years for multivariable analysis, then used the data to support this cutoff. This is data-driven dichotomization that inflates Type I error, reduces statistical power, and yields unstable estimates. The authors claim the cutoff was "informed by prior evidence," but the references cited (Quach 2012, Saito 2021, Park 2009) do not provide a validated threshold of 40 years for screening in Vietnam. Moreover, the authors did not pre-specify this cutoff in a protocol; they derived it from their own data (note that 88.4% of participants were ≥40 years, making the comparison highly unbalanced).

The correct approach would have been to model age as a continuous variable (using restricted cubic splines or fractional polynomials) or to use clinically justified cutoffs from external guidelines (e.g., 45 or 50 years). The authors' justification – "avoiding collinearity and preserving power" – is not acceptable.

Required action:

Re-analyze the data with age as a continuous variable (e.g., per 10-year increment) and report adjusted odds ratios.

If dichotomization is retained, provide a pre-specified analysis plan or external validation of the 40-year cutoff from an independent dataset.

Alternatively, present both continuous and dichotomized models in supplemental materials and clearly state that the dichotomized analysis is exploratory.

3. Inadequate Control for Confounding – Residual Bias Likely

The multivariable model adjusts for only six variables (age, sex, BMI, family history, smoking, alcohol). Numerous established CRN risk factors are omitted:

Diabetes mellitus / insulin resistance

Physical inactivity

Red/processed meat intake

Dietary fiber consumption

NSAID/aspirin use

Socioeconomic status (critical given the self-selected, wealthy cohort)

Previous screening history

The authors acknowledge this limitation but provide no sensitivity analyses (e.g., E-values, propensity score calibration, or negative control outcomes) to assess robustness to unmeasured confounding. Given the observational design and the self-selected nature of the cohort, residual confounding is likely substantial.

Required action:

Calculate E-values for the main findings (age, BMI, family history) to quantify the minimum strength of association that an unmeasured confounder would need to have with both exposure and outcome to explain away the observed effects.

Explicitly state that causal inferences cannot be drawn and that the reported associations may be confounded.

4. Underpowered Analysis for Advanced CRN – Findings Should Not Be Presented as Definitive

Only 64 participants had advanced CRN. The multivariable model includes 6 predictors, yielding an events-per-variable ratio of ~10.7 – at the absolute minimum acceptable threshold (some methodologists recommend ≥15-20). The resulting confidence intervals are extremely wide (e.g., age OR 5.20, 95% CI 1.22–22.11), and the null findings for BMI (OR 1.62, 95% CI 0.91–2.88) and family history (OR 2.01, 95% CI 0.92–4.40) should not be interpreted as evidence of no association. The authors acknowledge the limited statistical power, yet they present these results in Table 3 alongside the primary analysis without sufficient warning.

Required action:

Move the advanced CRN analysis to supplemental materials with a clear disclaimer that it is exploratory and underpowered.

Remove statements in the abstract and results that suggest any conclusion about advanced CRN beyond "age may be associated, but estimates are imprecise."

5. Unvalidated Definition of "Asymptomatic" – Risk of Misclassification

Asymptomatic status was determined by unstructured clinical interviews, not validated instruments (e.g., Rome IV criteria, structured symptom questionnaires). No information is provided on inter-rater reliability, the specific questions asked, or whether symptom assessment was blinded to colonoscopy findings. Recall bias is likely. Some participants may have had mild or intermittent symptoms (e.g., occasional abdominal discomfort) that were not captured, potentially misclassifying symptomatic individuals as asymptomatic and biasing prevalence estimates upward.

Required action:

Provide the actual symptom questionnaire or structured interview form as a supplemental file.

Report the proportion of participants who endorsed any symptom (even if considered minor) and compare their CRN prevalence to truly asymptomatic participants as a sensitivity analysis.

Acknowledge that the lack of a validated instrument is a major limitation.

6. NICE Type I Discordance Raises Quality Concerns – Inadequately Addressed

The authors report that 15.3% of lesions were endoscopically classified as NICE type I (suggesting hyperplastic) but histologically confirmed as neoplasia. This discordance rate is substantially higher than reported in high-quality optical diagnosis studies (where negative predictive value for adenoma is typically >90% for NICE type I). The authors attribute this to "known limitations of optical diagnosis" and note that endoscopists had ADRs >30%. However, an ADR >30% does not guarantee accurate optical characterization. The high discordance raises legitimate concerns about:

Real-time diagnostic accuracy of the participating endoscopists

Whether all lesions were adequately examined (e.g., adequate bowel preparation, withdrawal time, imaging quality)

Potential selection bias in which lesions were resected

Required action:

Provide a stratified analysis of NICE type I discordance by lesion size (≤5 mm vs. >5 mm) and location.

Report the inter-endoscopist variation in NICE classification accuracy.

Discuss whether any lesions classified as NICE type I were left in situ (i.e., not resected) – if so, the true prevalence of CRN may be underestimated.

7. Single-Center Design with 5 Endoscopists – Lack of External Validity

All colonoscopies were performed by 5 endoscopists at a single private facility. The results may reflect local practice patterns, lesion recognition skills, and patient demographics that are not representative of other regions in Vietnam (e.g., rural areas, public hospitals). The authors acknowledge this, but the discussion still overgeneralizes.

Required action:

Add a clear statement that external validation in multi-center, population-based samples is required before any screening policy recommendations can be derived from these data.

**********

7. PLOS authors have the option to publish the peer review history of their article (what does this mean?). If published, this will include your full peer review and any attached files.

If you choose “no”, your identity will remain anonymous but your review may still be made public.

**Do you want your identity to be public for this peer review?**  For information about this choice, including consent withdrawal, please see our Privacy Policy.

Reviewer #1: No

**********

[NOTE: If reviewer comments were submitted as an attachment file, they will be attached to this email and accessible via the submission site. Please log into your account, locate the manuscript record, and check for the action link "View Attachments". If this link does not appear, there are no attachment files.]

To ensure your figures meet our technical requirements, please review our figure guidelines: https://journals.plos.org/plosone/s/figures

You may also use PLOS’s free figure tool, NAAS, to help you prepare publication quality figures: https://journals.plos.org/plosone/s/figures#loc-tools-for-figure-preparation.

NAAS will assess whether your figures meet our technical requirements by comparing each figure against our figure specifications.

# Author comment: Author response to Decision Letter 4

**DOI:** 10.1371/journal.pone.0352998.r008

25 May 2026

May 24, 2026

Dear Editor,

We would like to thank you very much for your kindness in allowing us to revise our manuscript. We also thank the reviewers for their constructive comments. The manuscript has been revised accordingly, and the revised sections in the resubmitted manuscript have been highlighted in blue. We would like to respond to your comments as follows.

Response to Reviewer 1

Comment 1. Severe selection bias undermines generalizability (unchanged from prior review).

Critical issue: The prevalence estimates (26.2% overall CRN, 9.0% advanced CRN) cannot be extrapolated to the general population. The authors correctly note that the study was "not designed to provide population-level prevalence estimates," yet the abstract, results, and discussion repeatedly imply broader relevance. For a high-impact journal, a single-center, self-selected cohort cannot support meaningful prevalence estimates – even as a “hypothesis-generating” study, the bias is too large to justify any conclusion about the Vietnamese population.

Required action:

Revise the title and abstract to explicitly state that findings are from a self-selected screening-attending cohort in a private clinical setting, not representative of asymptomatic Vietnamese adults.

Remove or substantially tone down statements implying generalizability (e.g., "CRN was prevalent in asymptomatic Vietnamese adults" should become "CRN was prevalent in this self-selected screening cohort").

Add a dedicated paragraph in the Discussion quantifying the expected direction and magnitude of selection bias.

Reply:

We thank the Reviewer for the valuable comment regarding selection bias and overgeneralizability. Accordingly, we have: (1) revised the Title, Abstract, Introduction, and Discussion to clearly state that this was a self-selected, screening-attending cohort recruited in a private clinical setting; (2) replaced generalized statements “CRN was prevalent in asymptomatic Vietnamese adults” with more accurate wording “CRN was prevalent in this self-selected screening cohort”; and (3) added a dedicated paragraph in the Discussion quantifying the expected direction and magnitude of selection bias.

Title

“Prevalence and risk factors for colorectal neoplasia in a self-selected Vietnamese screening cohort undergoing self-funded colonoscopy”

Abstract (page 2, line 34; page 3, lines 42-43)

“In this screening-attending cohort, the prevalence of overall CRN and advanced CRN were 26.2% and 9.0%, respectively.”

“CRN was prevalent in this self-selected screening-attending cohort in a private clinical setting”.

Introduction (page 5, lines 73-74)

“Consequently, identifying the prevalence and associated risk factors for CRN may contribute preliminary evidence relevant to future CRC screening strategies in Vietnam.”

Discussion (page 19, lines 359-367; page 20, lines 396-397; page 21, lines 403-404)

“This study had several limitations. First, it was a single-center study involving individuals who voluntarily underwent self-funded screening colonoscopy in a private clinical setting, which may have introduced selection bias. Participants were likely more health-conscious and had better healthcare access than the general Vietnamese population, potentially resulting in higher prevalence estimates than those in the broader average-risk population. Participants were likely more health-conscious and had better healthcare access than the general Vietnamese population, potentially resulting in higher prevalence estimates than those in the broader average-risk population.”

“In this screening-attending population, our data suggests a notable burden of CRN within this screening-attending cohort.”

“In conclusion, CRN was prevalent in this self-selected screening-attending cohort undergoing self-funded colonoscopy.”

Comment 2. Post-hoc age dichotomization is a serious methodological flaw

Re-analyze the data with age as a continuous variable (e.g., per 10-year increment) and report adjusted odds ratios.

If dichotomization is retained, provide a pre-specified analysis plan or external validation of the 40-year cutoff from an independent dataset.

Alternatively, present both continuous and dichotomized models in supplemental materials and clearly state that the dichotomized analysis is exploratory.

Reply:

We thank the reviewer for this important methodological comment. We agree that modeling age as a continuous variable is statistically more appropriate than relying solely on dichotomized thresholds. Accordingly, we re-analyzed the data using age as a continuous variable expressed per 10-year increment in the primary multivariable logistic regression models. These revised analyses have been incorporated into the Methods, Results, Table 3, and Discusssion of the revised manuscript.

We retained the age threshold analysis of ≥ 40 years as an exploratory secondary analysis because previous Vietnamese and Asia-Pacific studies have suggested a relatively younger age distribution of CRN in this region. The manuscript has been revised to clarify that this cutoff was exploratory and was not intended to define an optimal screening age.

Methods (page 9, lines 184-188)

“In the primary multivariable logistic regression analysis, age was modeled as a continuous variable expressed per 10-year increment. An exploratory secondary analysis using an age cutoff of 40 years was also performed because previous Vietnamese and Asia-Pacific studies have suggested a relatively younger age distribution of CRN in this region [4, 10].”

Results (page 13, lines 225-229; page 13, lines 233-237)

“In the primary multivariable analysis, increasing age per 10-year increment (odds ratio [OR]: 1.76; 95% CI: 1.47-2.11; p < 0.001), BMI ≥ 23 kg/m² (OR: 1.70; 95% CI: 1.16-2.50; p = 0.006), alcohol consumption (OR: 1.83; 95% CI: 1.10-3.04; p = 0.020), and family history of CRC (OR: 2.43; 95% CI: 1.36-4.37; p = 0.003) were independently associated with overall CRN (Table 3).”

Table 3: Multivariable logistic regression analysis of factors associated with colorectal neoplasia

Risk factors Overall colorectal neoplasia

OR 95% CI p value

Male 1.16 0.71-1.91 0.551

Age (per 10-year increase) 1.76 1.47- 2.11 < 0.001

BMI ≥ 23 kg/m2 1.70 1.16-2.50 0.006

Smoking 1.24 0.72-2.11 0.440

Alcohol consumption 1.83 1.10-3.04 0.020

Family history of CRC 2.43 1.36-4.37 0.003

OR: odds ratio, CI: confidence interval, BMI: Body mass index, CRC: Colorectal cancer

“In the exploratory secondary analysis using an age threshold of ≥40 years, participants aged ≥40 years showed higher odds of overall CRN (OR: 2.79; 95% CI: 1.40-5.56; p = 0.004). BMI ≥23 kg/m2 (OR: 1.92; 95% CI: 1.32-2.79; p = 0.001) and family history of CRC (OR: 2.29; 95% CI: 1.29-4.05; p = 0.005) were also associated with overall CRN.”

Supplementary Table 2: Exploratory multivariable logistic regression analysis of factors associated with colorectal neoplasia and advanced colorectal neoplasia

Risk factors Overall colorectal neoplasia Advanced colorectal neoplasia

OR 95% CI p value OR 95% CI p value

Male 1.15 0.71-1.86 0.575 1.44 0.70-2.97 0.322

Age ≥ 40 2.79 1.40- 5.56 0.004 5.20 1.22-22.11 0.025

BMI ≥ 23 1.92 1.32-2.79 0.001 1.62 0.91-2.88 0.102

Smoking 1.32 0.78-2.23 0.307 0.75 0.35-1.61 0.461

Alcohol consumption 1.47 0.91-2.39 0.117 1.58 0.77-3.23 0.212

Family history of CRC 2.29 1.29-4.05 0.005 2.01 0.92-4.40 0.082

OR: odds ratio, CI: confidence interval, BMI: Body mass index, CRC: Colorectal cancer

Discussion (page 14, lines 292-313)

“In the primary multivariable analysis, increasing age modeled as a continuous variable per 10-year increment remained independently associated with both overall CRN and advanced CRN. BMI ≥ 23 kg/m², alcohol consumption, and family history of CRC were also independent risk factors for overall CRN. These findings are consistent with those from a large prospective multinational study in asymptomatic Asian populations, which identified advanced age, male sex, and family history of CRC as key risk factors for CRN [25]. Previous studies have also indicated that increasing age is an important risk factor for the development of CRN and CRC [22, 24]. In Vietnam, several studies have suggested a relatively younger age distribution of CRN and CRC compared with that reported in some other countries. A retrospective study conducted on 2,525 outpatients with advanced CRN revealed that the rate of advanced CRN in the group under 50 years of age was 23.4%, whereas that in the group < 40 years of age was 8.2% [13]. Another prospective study reported a significantly high proportion of early-onset CRC (i.e., < 50 years of age) of 28%, of which 11% of patients were under 40 years of age [4]. Therefore, these data suggest a potential burden of CRC in younger adults within screening-attending populations. Based on these prior observations, we additionally performed an exploratory secondary analysis using an age threshold of ≥ 40 years. In this exploratory model, individuals aged ≥40 years had significantly increased odds of both overall CRN and advanced CRN. These observations highlight the need for further investigation into age-specific risk stratification in this context. However, this observation should be interpreted as hypothesis-generating, and confirmation from large-scale, population-based studies is required before informing national policy.”

Comment 3. Inadequate control for confounding

Calculate E-values for the main findings (age, BMI, family history) to quantify the minimum strength of association that an unmeasured confounder would need to have with both exposure and outcome to explain away the observed effects.

Explicitly state that causal inferences cannot be drawn and that the reported associations may be confounded.

Reply:

We thank the reviewer for the valuable comment. We acknowledge that residual confounding from unmeasured variables remains possible because of the observational and self-selected nature of the cohort.

To further evaluate the robustness of the observed associations, we calculated E-values for the main findings in the primary multivariable analysis. The E-values were 1.98 for age per 10-year increment, 1.93 for BMI ≥ 23 kg/m², 2.04 for alcohol consumption, and 2.49 for family history of CRC (Supplementary Table 3). These findings suggest that unmeasured confounding of at least moderate magnitude would be required to fully explain away the observed associations.

We have revised the Discussion to explicitly state that causal inferences cannot be drawn from this cross-sectional observational study and that the reported associations should be interpreted cautiously because residual confounding may persist despite multivariable adjustment (page 20, lines 381-389; page 20, lines 394-395).

“Third, some factors potentially associated with CRN, such as diabetes mellitus, dyslipidemia, physical inactivity, and dietary factors including high red meat intake and low fiber consumption, were not considered in our study. Furthermore, smoking and alcohol consumption were categorized using simplified definitions and were not evaluated quantitatively. These limitations may introduce residual confounding, and the observed associations should therefore be interpreted cautiously. E-value analyses indicated that unmeasured confounding of at least moderate magnitude would be required to fully explain away the observed associations (Supplementary Table 3).”

“Fifth, owing to the cross-sectional design of this study, temporal relationships cannot be established, and therefore causal inferences cannot be made.”

Supplementary Table 3. E-values for significant factors associated with colorectal neoplasia in the primary multivariable analysis

Risk factors Adjusted Odds Ratio (95% CI)* E-value for point estimate E-value for lower bound of 95% CI

Age (per 10-year increment) 1.76 (1.47 – 2.11) 1.98 1.72

BMI ≥ 23 kg/m² 1.70 (1.16 – 2.50) 1.93 1.37

Alcohol consumption 1.83 (1.10 – 3.04) 2.04 1.28

Family history of CRC 2.43 (1.36 – 4.37) 2.49 1.61

OR: odds ratio, CI: confidence interval, BMI: Body mass index, CRC: Colorectal cancer.

E-value was calculated with the following formula for colorectal neoplasia prevalence > 15%:

Comment 4. Underpowered analysis for advanced CRN

Move the advanced CRN analysis to supplemental materials with a clear disclaimer that it is exploratory and underpowered.

Remove statements in the abstract and results that suggest any conclusion about advanced CRN beyond "age may be associated, but estimates are imprecise."

Reply:

We thank the reviewer for this helpful comment. We agree that the number of advanced CRN cases was relatively limited, resulting in wider confidence intervals and potentially unstable estimates. Accordingly, the multivariable analysis for advanced CRN has been moved to Supplementary Materials. We also checked the Abstract and revised the Results to clarify that advanced CRN analysis should be considered exploratory (page 13, lines 229-232; page 13, lines 237-240).

“For advanced CRN, increasing age may be associated with the outcome in the exploratory multivariable analysis; however, the estimates were imprecise, with relatively wide confidence intervals because of the limited number of advanced CRN cases (Supplementary Table 1).”

“Similarly, participants aged ≥ 40 years showed a possible increase in the odds of advanced CRN in the exploratory analysis, although the estimates were imprecise because of the limited number of events (OR: 5.20; 95% CI: 1.22-22.11; p = 0.025) (Supplementary Table 2).”

Supplementary Table 1: Exploratory multivariable logistic regression analysis of factors associated with advanced colorectal neoplasia

Risk factors Advanced colorectal neoplasia

OR 95% CI p value

Male 1.42 0.67-2.98 0.360

Age (per 10-year increase) 1.91 1.46-2.51 < 0.001

BMI ≥ 23 kg/m2 1.43 0.79-2.56 0.234

Smoking 0.71 0.33-1.55 0.394

Alcohol consumption 2.00 0.94-4.26 0.071

Family history of CRC 2.06 0.93-4.58 0.077

OR: odds ratio, CI: confidence interval, BMI: Body mass index, CRC: Colorectal cancer

Comment 5. Unvalidated definition of “asymptomatic”

Provide the actual symptom questionnaire or structured interview form as a supplemental file.

Report the proportion of participants who endorsed any symptom (even if considered minor) and compare their CRN prevalence to truly asymptomatic participants as a sensitivity analysis.

Acknowledge that the lack of a validated instrument is a major limitation.

Reply:

We thank the reviewer for this thoughtful comment. Asymptomatic status in our study was determined through routine clinical interviews rather than a formally validated diagnostic instrument. No formal standardized questionnaire was used during routine clinical assessment; therefore, no structured symptom questionnaire is available as supplementary material. During the pre-procedural evaluation, experienced gastroenterologists utilized a consistent clinical framework to assess lower gastrointestinal symptoms. Participants reporting gastrointestinal symptoms, including mild or intermittent complaints, were excluded as part of the study eligibility criteria. The final dataset did not intentionally include mildly symptomatic individuals; therefore, a comparative sensitivity analysis could not be performed. We have explicitly acknowledged the lack of a validated instrument and the potential for symptom misclassification and recall bias in the revised manuscript (pages 19-20, lines 376-381).

“Second, although asymptomatic status was determined through structured clinical assessment by experienced gastroenterologists—as is typical in routine screening practice—it was not based on a formally validated instrument. Therefore, mild or intermittent gastrointestinal symptoms may have been underrecognized, potentially resulting in some degree of symptom misclassification and recall bias, which should be considered when interpreting the observed prevalence estimates.”

Comment 6. NICE type I discordance highlights quality issue

Provide a stratified analysis of NICE type I discordance by lesion size (≤5 mm vs. >5 mm) and location.

Report the inter-endoscopist variation in NICE classification accuracy.

Discuss whether any lesions classified as NICE type I were left in situ (i.e., not resected) –

Attachment Submitted filename: Response - Plos One - R4.docx

# Peer review: Decision Letter 4

**DOI:** 10.1371/journal.pone.0352998.r009

**Authors:** Chih-Wei Tseng

17 Jun 2026

Prevalence and risk factors for colorectal neoplasia in a self-selected Vietnamese screening cohort undergoing self-funded colonoscopy

PONE-D-25-66438R4

Dear Dr. Quach,

We’re pleased to inform you that your manuscript has been judged scientifically suitable for publication and will be formally accepted for publication once it meets all outstanding technical requirements.

Within one week, you’ll receive an e-mail detailing the required amendments. When these have been addressed, you’ll receive a formal acceptance letter and your manuscript will be scheduled for publication.

An invoice will be generated when your article is formally accepted. Please note, if your institution has a publishing partnership with PLOS and your article meets the relevant criteria, all or part of your publication costs will be covered. Please make sure your user information is up-to-date by logging into Editorial Manager at Editorial Manager® and clicking the ‘Update My Information' link at the top of the page. For questions related to billing, please contact billing support.

If your institution or institutions have a press office, please notify them about your upcoming paper to help maximize its impact. If they’ll be preparing press materials, please inform our press team as soon as possible -- no later than 48 hours after receiving the formal acceptance. Your manuscript will remain under strict press embargo until 2 pm Eastern Time on the date of publication. For more information, please contact onepress@plos.org.

Kind regards,

Chih-Wei Tseng

Academic Editor

PLOS One

Additional Editor Comments (optional):

Although the authors have addressed all comments, the reviewer still has some concerns regarding the article. However, I believe that the authors have made their best efforts to improve the manuscript and have appropriately acknowledged the issues that could not be fully resolved as study limitations. Therefore, I suggest that acceptance of this article may be considered.

Reviewers' comments:

Reviewer's Responses to Questions

**Comments to the Author**

1. If the authors have adequately addressed your comments raised in a previous round of review and you feel that this manuscript is now acceptable for publication, you may indicate that here to bypass the “Comments to the Author” section, enter your conflict of interest statement in the “Confidential to Editor” section, and submit your "Accept" recommendation.

Reviewer #1: All comments have been addressed

**********

2. Is the manuscript technically sound, and do the data support the conclusions?

The manuscript must describe a technically sound piece of scientific research with data that supports the conclusions. Experiments must have been conducted rigorously, with appropriate controls, replication, and sample sizes. The conclusions must be drawn appropriately based on the data presented.

Reviewer #1: Partly

**********

3. Has the statistical analysis been performed appropriately and rigorously?

Reviewer #1: No

**********

4. Have the authors made all data underlying the findings in their manuscript fully available?

The PLOS Data policy requires authors to make all data underlying the findings described in their manuscript fully available without restriction, with rare exception (please refer to the Data Availability Statement in the manuscript PDF file). The data should be provided as part of the manuscript or its supporting information, or deposited to a public repository. For example, in addition to summary statistics, the data points behind means, medians and variance measures should be available. If there are restrictions on publicly sharing data—e.g. participant privacy or use of data from a third party—those must be specified.

Reviewer #1: Yes

**********

5. Is the manuscript presented in an intelligible fashion and written in standard English?

PLOS ONE does not copyedit accepted manuscripts, so the language in submitted articles must be clear, correct, and unambiguous. Any typographical or grammatical errors should be corrected at revision, so please note any specific errors here.

Reviewer #1: Yes

**********

6. Review Comments to the Author

Please use the space provided to explain your answers to the questions above. You may also include additional comments for the author, including concerns about dual publication, research ethics, or publication ethics. (Please upload your review as an attachment if it exceeds 20,000 characters)

Reviewer #1: 1. Selection bias is fatal to generalizability (unresolvable)

The problem: Self-selected, self-funded, single-center cohort from a private clinic. Participants were "likely more health-conscious and had better healthcare access" – the authors admit this but cannot quantify or correct for it.

Why this matters for high-impact journals:

Prevalence estimates (26.2% CRN, 9.0% advanced CRN) cannot inform screening policy or population risk

The authors' own E-values (1.98-2.49) show that moderate unmeasured confounding could explain all associations

The Vietnam population census standardization (age-standardized prevalence 21.3%) is statistically correct but clinically meaningless when the source cohort is non-representative

What the authors did: Added caveats and a limitations paragraph. This is insufficient for high-impact publication where actionable conclusions are expected.

What would be needed: A population-based sampling frame. Without it, this remains a hypothesis-generating descriptive study of a niche cohort.

2. The "asymptomatic" definition is unvalidated and potentially circular

The problem: No validated instrument was used. Asymptomatic status was determined by "structured clinical assessment" – but the authors admit "no formal standardized questionnaire was used" and "no structured symptom questionnaire is available."

Critical gap: The authors could not perform the requested sensitivity analysis comparing "truly asymptomatic" to "mildly symptomatic" because they did not collect granular symptom data. This means we cannot rule out that some participants had subtle symptoms that prompted testing – a classic indication bias.

What the authors did: Acknowledged the limitation. This is honest but does not fix the problem.

High-impact standard: Symptom assessment requires validated instruments (e.g., Rome criteria, GSRS) with inter-rater reliability data.

3. Advanced CRN analysis is underpowered despite being moved to supplements

The problem: Only 64 advanced CRN cases. The authors moved this analysis to supplements with disclaimers, but the abstract still reports 9.0% advanced CRN prevalence as a primary finding.

The inconsistency: If the analysis is too underpowered for meaningful inference, why is the prevalence still featured prominently? The abstract should state clearly that advanced CRN estimates are imprecise (95% CI: 7.0%-11.3% is wide but not enormous – the real issue is the risk factor analysis, not the prevalence estimate itself).

What the authors did: Moved multivariable analysis for advanced CRN to supplements. This is appropriate, but the prevalence itself is still presented as a key finding.

What remains problematic: The exploratory age ≥40 years OR for advanced CRN is 5.20 with CI 1.22-22.11 – this is statistically significant but based on very few events. The authors appropriately call this "hypothesis-generating," but high-impact journals typically require larger event numbers for such claims.

4. The 40-year age threshold remains post-hoc and exploratory (appropriately labeled, but still problematic)

The problem: No pre-specified analysis plan. The authors now correctly label this as "exploratory secondary analysis," but the Introduction and Discussion still heavily imply clinical relevance of the 40-year threshold, citing Japanese screening guidelines starting at age 40.

The logical leap: Japan has population-based data supporting age 40 screening. Vietnam does not. The authors are using their exploratory finding to suggest "a potential burden of CRC in younger adults" – but without a representative sample, this is speculative.

What the authors did: Moved the dichotomized analysis to supplements, labeled it exploratory, and modeled age continuously in the primary analysis. This is methodologically correct.

What remains: The Discussion (lines 304-312) states: "these data suggest a potential burden of CRC in younger adults within screening-attending populations" – but the data only show this in their specific self-selected cohort. The leap to "younger adults" generally is unsupported.

Minor but Notable Issues

5. NICE classification discordance analysis is post-hoc and underpowered for inter-endoscopist variation

The authors added a stratified analysis showing discordance was higher in lesions ≤5 mm (25.0% vs. 4.9%). However, they state "no significant inter-endoscopist variation in NICE classification accuracy was observed" – but no statistical test or data are provided to support this claim. How many endoscopists? What were their individual discordance rates? Was there a formal kappa statistic?

6. Alcohol consumption definition is overly broad

"Drinking more than once per month" is a very low threshold that likely captures many very light drinkers. This may dilute the true effect of moderate/heavy drinking. The authors acknowledge this limitation.

7. Family history definition lacks specificity

"At least one affected first-degree relative at any age" – no distinction by number of relatives, age of onset, or whether the relative actually had screening-detected vs. symptomatic CRC. This is a common limitation but worth noting.

8. E-value interpretation is over-optimistic

The authors state unmeasured confounding "of at least moderate magnitude would be required" to explain away associations. However, E-values of ~2.0 mean that an unmeasured confounder associated with both the exposure and outcome by OR of ~2.0 could fully explain the effect. In observational epidemiology, such confounders are common (e.g., socioeconomic status, health-seeking behavior, diet). The authors do not discuss this adequately.

**********

7. PLOS authors have the option to publish the peer review history of their article (what does this mean?). If published, this will include your full peer review and any attached files.

If you choose “no”, your identity will remain anonymous but your review may still be made public.

**Do you want your identity to be public for this peer review?**  For information about this choice, including consent withdrawal, please see our Privacy Policy.

Reviewer #1: No

**********

# Editor report: Acceptance letter

**DOI:** 10.1371/journal.pone.0352998.r010

**Authors:** Chih-Wei Tseng

PONE-D-25-66438R4

PLOS One

Dear Dr. Quach,

I'm pleased to inform you that your manuscript has been deemed suitable for publication in PLOS One. Congratulations! Your manuscript is now being handed over to our production team.

At this stage, our production department will prepare your paper for publication. This includes ensuring the following:

* All references, tables, and figures are properly cited

* All relevant supporting information is included in the manuscript submission,

* There are no issues that prevent the paper from being properly typeset

You will receive further instructions from the production team, including instructions on how to review your proof when it is ready. Please keep in mind that we are working through a large volume of accepted articles, so please give us a few days to review your paper and let you know the next and final steps.

Lastly, if your institution or institutions have a press office, please let them know about your upcoming paper now to help maximize its impact. If they'll be preparing press materials, please inform our press team within the next 48 hours. Your manuscript will remain under strict press embargo until 2 pm Eastern Time on the date of publication. For more information, please contact onepress@plos.org.

You will receive an invoice from PLOS for your publication fee after your manuscript has reached the completed accept phase. If you receive an email requesting payment before acceptance or for any other service, this may be a phishing scheme. Learn how to identify phishing emails and protect your accounts at https://explore.plos.org/phishing.

If we can help with anything else, please email us at customercare@plos.org.

Thank you for submitting your work to PLOS ONE and supporting open access.

Kind regards,

PLOS ONE Editorial Office Staff

on behalf of

Dr. Chih-Wei Tseng

Academic Editor

PLOS One
